# Klotho‐Derived Peptide 1 Protects against Acute Kidney Injury by Directly Targeting Mitochondrial ATAD3A

**DOI:** 10.1002/advs.77214

**Published:** 2026-08-25

**Authors:** Xiaoyao Zhang, Shihui Lin, Tianyu Wu, Zhixin Zhang, Hong Zhou, Xue Hong, Youhua Liu

**Affiliations:** ^1^ State Key Laboratory of Multi‐organ Injury Prevention and Treatment National Clinical Research Center for Kidney and Urological Diseases Division of Nephrology Nanfang Hospital Southern Medical University Guangzhou China; ^2^ Guangdong Provincial Key Laboratory of Renal Failure Research Guangdong Provincial Institute of Nephrology Guangzhou China

**Keywords:** AKI, apoptosis, ATAD3A, Klotho, KP1, mitochondrial dysfunction

## Abstract

Acute kidney injury (AKI) is a clinical syndrome associated with severe morbidity and high mortality, for which there are no currently effective therapies. Aging, a state associated with Klotho protein decline, is an independent risk factor for AKI development and progression. Here, we report that Klotho‐derived peptide 1 (KP1), a small peptide that recapitulates the renoprotective potential of Klotho, effectively protects against AKI in mouse models induced by either cisplatin or ischemia‐reperfusion injury. KP1 treatment improved kidney function, ameliorated structural damage, inhibited tubular cell apoptosis, and preserved mitochondrial integrity in both models. Mechanistically, KP1 entered kidney proximal tubular epithelial cells via endocytosis, directly targeted the mitochondrial protein ATPase family AAA domain‐containing protein 3A (ATAD3A), and prevented its degradation, and preserved its function. By interacting with the hypoxia inducible gene 1 (HIG1) domain family member 2A (HIGD2A) and maintaining its expression and function within the mitochondria, ATAD3A prevented cytochrome c release and inhibited caspase activation following injury, thereby alleviating renal tubular cell apoptosis. Collectively, these studies demonstrate that KP1 is a promising therapeutic agent for AKI by directly targeting and preserving mitochondrial integrity. Our findings also lay the groundwork for developing novel therapeutic strategies to treat diseases associated with mitochondrial dysfunction.

## Graphical abstract

This study uncovers that KP1, a Klotho‐derived peptide, protects the kidney against acute kidney injury, a clinical syndrome with high morbidity and mortality. KP1 enters tubular epithelial cells via endocytosis, directly interacts with the mitochondrial ATAD3A/HIGD2A complex, thereby preventing cytochrome c release, and caspases activation, and apoptosis. KP1 could be a promising therapeutic agent for AKI.

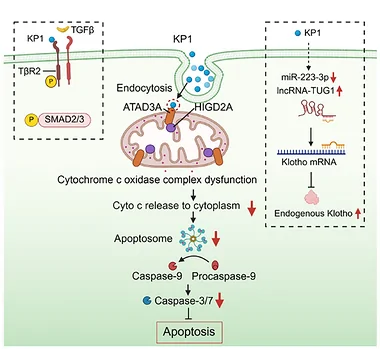

## 1 Introduction

Acute kidney injury (AKI) is a clinical syndrome characterized by a sudden decline in renal function, typically diagnosed based on elevated serum creatinine and/or oliguria [1, 2]. Afflicting 10%–50% of hospitalized patients, AKI serves as a major precipitant of multiple organ dysfunction syndrome and a significant risk factor for developing chronic kidney disease (CKD) [1]. Notably, aging is an independent and prominent risk factor for AKI [3], and elderly individuals exhibit a 3 to 8‐fold higher incidence compared to younger populations and face elevated risks of CKD progression and mortality [4]. Currently, there are no specific therapies proven to accelerate renal recovery in AKI. Clinical management for AKI patients at present remains focused on prevention, early detection, and mitigation of secondary injury.

The limitation of therapeutic options for AKI stems from our incomplete understanding of its pathogenic mechanisms, in which altered renal hemodynamics, inflammation, oxidative stress, and tubular cell death are the principal events. Notably, all of these features in AKI are directly or indirectly linked to, and converge on mitochondrial dysfunction [3, 5], which impairs cellular energy metabolism and signaling pathways, perpetuates renal inflammation, and drives programmed tubular cell death, collectively undermining renal structural and functional integrity. The causes of mitochondrial dysfunction in AKI are multifactorial [5], encompassing ultrastructural lesions such as cristae fragmentation and matrix swelling [6], as well as the loss of key architectural regulators including optic atrophy 1 (OPA1) and the sorting and assembly machinery 50 (Sam50) [7]. Functional impairments include respiratory chain dysfunction [8] and the leakage of mitochondrial constituents, such as mtDNA and cytochrome c [9], thereby triggering inflammation and apoptosis. In this context, developing a strategy to protect mitochondria from injury could represent a novel and effective approach to treat AKI patients [10].

The anti‐aging protein α‐Klotho, hereafter referred to as Klotho, is abundantly expressed in normal kidney tubular cells and exerts pleiotropic renoprotective effects by suppressing cellular senescence and apoptosis, attenuating oxidative stress, inducing autophagy, and alleviating kidney fibrosis and bone‐mineral dysregulation [11]. Klotho expression is inversely correlated with AKI development and progression, and supplementation with exogenous Klotho effectively inhibits tubular cell apoptosis and ameliorates kidney injury [12, 13]. Emerging evidence indicates that the renoprotective effects of Klotho are associated with its ability to preserve mitochondrial integrity and function [14]. For instance, Klotho has been shown to modulate redox homeostasis [15, 16], inhibit apoptosis [17], and enhance autophagic flux [18]. However, the mechanistic basis for Klotho‐mediated mitochondrial protection remains elusive.

Despite its therapeutic promise for AKI, Klotho replacement therapy is hampered by its large size, structural complexity, and high production cost. To overcome this hurdle, we identified Klotho‐derived peptide 1 (KP1), a novel 30‐amino acid peptide derived from human Klotho protein, which recapitulates its renoprotective potential. KP1 was discovered through systematic screening of a peptide library derived from Klotho protein, based on its ability to block TGF‐β signaling [19]. We found that KP1 binds to TGF‐β receptor II (TβR2), inhibits TGF‐β/Smad signaling, and suppresses the expression of profibrotic genes [19]. Furthermore, KP1 restores endogenous Klotho expression through post‐transcriptional regulation involving miR‐223‐3p and LncRNA‐TUG1 and mitigates cellular senescence in fibrotic kidneys [20]. However, whether KP1 regulates mitochondrial integrity in the context of AKI remains completely unknown.

In this study, we investigated the protective potential of KP1 in AKI, with a focus on its effects on mitochondrial dysfunction. We found that KP1 enters tubular cells via endocytosis and directly binds to mitochondrial ATPase family AAA‐domain‐containing protein 3A (ATAD3A), thereby stabilizing mitochondrial architecture to preserve renal function. Our findings not only establish KP1 as a promising therapeutic candidate for AKI, but also reveal a novel mitochondria‐targeted strategy for kidney protection.

## 2 Results

### 2.1 KP1 Protects Against both Nephrotoxic and Ischemic AKI in Mice

**FIGURE 1:**
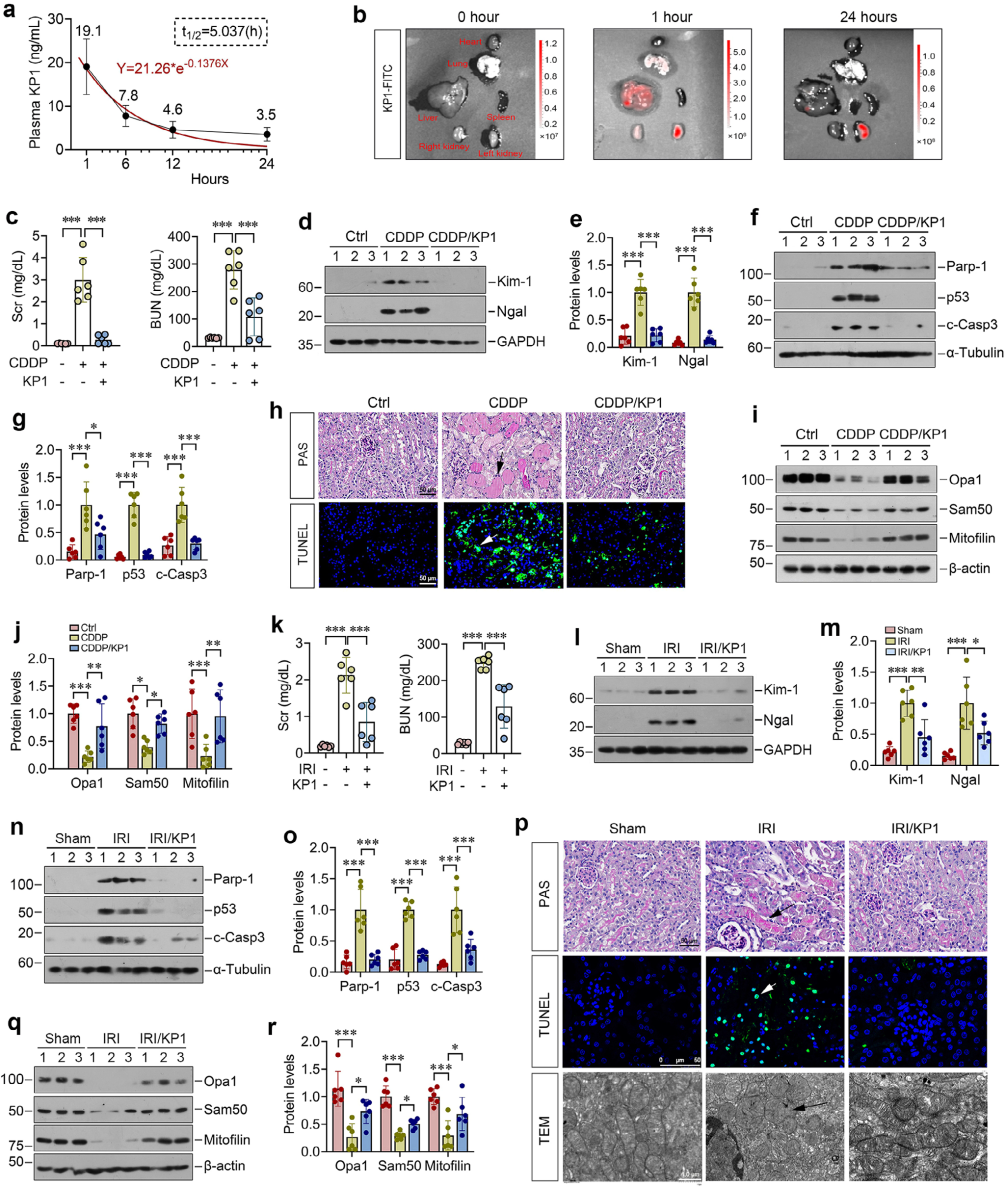
KP1 protects against both nephrotoxic and ischemic AKI in mice. (a) Pharmacokinetic profile of KP1 in mouse plasma. Plasma KP1 concentrations were determined by peptidomic mass spectrometry at 1, 6, 12, and 24 h after intravenous injection. The elimination half‐life (t_1/2_ = 5.037 h) was derived from the fitted exponential decay curve Y = 21.26*e^−0.1376X^. (b) Organ imaging showed that KP1 was preferentially accumulated in the injured kidney at 1 day after UIRI. Relative levels of FITC‐KP1 are shown in major organs (heart, lung, liver, spleen, right kidney, left kidney) at 0, 1, and 24 h after injection. (c) Graphic presentation shows the serum creatinine (Scr) and blood urea nitrogen (BUN) levels in CDDP mice after KP1 treatment. (d,e) Western blotting analysis for Kim‐1 and Ngal protein levels in CDDP mice after KP1 treatment. (f,g) Western blotting analysis for Parp‐1, p53 and c‐Casp3 protein levels in CDDP mice after KP1 treatment. (h) Representative Periodic acid‐Schiff (PAS) and TUNEL staining micrograghs in CDDP mice after KP1 treatment. Scale bar, 50 µm. (i) Representative Western blot analyses of Opa1, Sam50 and mitofilin protein levels in CDDP mice after KP1 treatment. (j) Quantitative data of Opa1, Sam50 and mitofilin protein levels in CDDP mice after KP1 treatment. (k) Graphic presentation shows the Scr and BUN levels in IRI mice after KP1 treatment. (l, m) Western blotting analysis for Kim‐1 and Ngal protein levels in IRI mice after KP1 treatment. (n,o) Western blotting analysis for Parp‐1, p53 and c‐Casp3 protein levels in IRI mice after KP1 treatment. (p) Representative transmission electron micrographs (TEM), PAS and TUNEL staining micrograghs in IRI mice after KP1 treatment. (q,r) Western blotting analysis for Opa1, Sam50 and mitofilin protein levels in IRI mice after KP1 treatment. ^*^ *p* < 0.05, ^**^ *p* < 0.01, ^***^ *p* < 0.001 (n = 6). ns, no statistical difference.

We first assessed the stability of KP1 in mouse plasma after a single intravenous injection. Mass spectrometry assay revealed that the half‐life of KP1 in the plasma exceeded 5 h (Figure 1a). To investigate the tissue distribution of KP1 after intravenous injection, we utilized a mouse model of unilateral ischemia‐reperfusion injury (UIRI), in which one kidney was intact. At 24 h after UIRI, FITC‐tagged KP1 was injected intravenously, and major organs including the kidneys, lungs, liver, heart and spleen were collected at 1 h and 24 h thereafter. As shown in Figure 1b, FITC‐KP1 was detected in the liver, lung, and kidneys at 1 h after injection. Compared with the intact kidney (right), FITC‐KP1 was more abundant in the injured one (left). By 24 h after injection, KP1 was predominantly localized in the injured kidney, but not in other organs (Figure 1b). Therefore, it appears that KP1 was preferentially accumulated in the injured kidney at 24 h post‐injection.

We then examined the potential role of KP1 in AKI induced by cisplatin (CDDP), a nephrotoxic drug widely used in the clinic for chemotherapy of cancer patients. As shown in Figure 1c, serum creatinine (Scr) and blood urea nitrogen (BUN) assays revealed that KP1 ameliorated CDDP‐induced kidney dysfunction. KP1 attenuated CDDP‐induced upregulation of renal injury markers such as kidney injury molecule‐1 (Kim‐1) and neutrophil gelatinase‐associated lipocalin (Ngal) (Figure 1d,e), as well as apoptosis markers including poly(ADP‐ribose) polymerase 1 (Parp‐1), p53, cleaved caspase‐3 (c‐Casp3) (Figure 1f,g). Immunohistochemical staining also demonstrated that Kim‐1 and c‐Casp3 expression were abolished in renal tubular epithelium in KP1‐treated mice (Figure S1a). Periodic acid‐Schiff (PAS) staining revealed that KP1 prevented CDDP‐induced histopathological alterations, including tubular epithelial flattening, cell death, basement membrane rupture, tubular dilation, and prominent cast formation (Figure 1h). TUNEL staining also demonstrated that KP1 largely abolished tubular cell apoptosis induced by CDDP in the kidneys (Figure 1h). We found that CDDP‐induced AKI was associated with depletion of mitochondrial proteins OPA1, Sam50, and mitofilin, which were restored by KP1 (Figure 1i,j), suggesting a special role of KP1 in protecting against mitochondrial dysfunction in AKI.

To generalize these findings, we employed ischemic AKI induced by bilateral ischemia‐reperfusion injury (IRI). As shown in Figure 1k, KP1 also reduced IRI‐induced Scr and BUN levels. KP1 abolished IRI‐upregulated Kim‐1, Ngal, Parp‐1, p53, and c‐Casp3 (Figure 1l–o). Histologically, KP1 prevented IRI‐induced Kim‐1 and c‐Casp3 expression (Figure S1b), as well as pathological alterations and apoptosis (Figure 1p). Notably, KP1 conferred mitochondrial protection by restoring IRI‐depleted OPA1, Sam50, and mitofilin (Figure 1q,r). Electron microscopy also confirmed that KP1 preserved the ultrastructural integrity of mitochondria in tubular cells by preventing IRI‐induced fragmentation, spherical transformation, membrane blurring, and cristae disorganization (Figure 1p). Notably, KP1 did not show any adverse side effects in normal mice, even at the dose as high as 10 mg/kg/day for 4 weeks (Figure S2).

### 2.2 KP1 Prevents Renal Tubular Injury and Mitochondrial Dysfunction in Vitro

**FIGURE 2:**
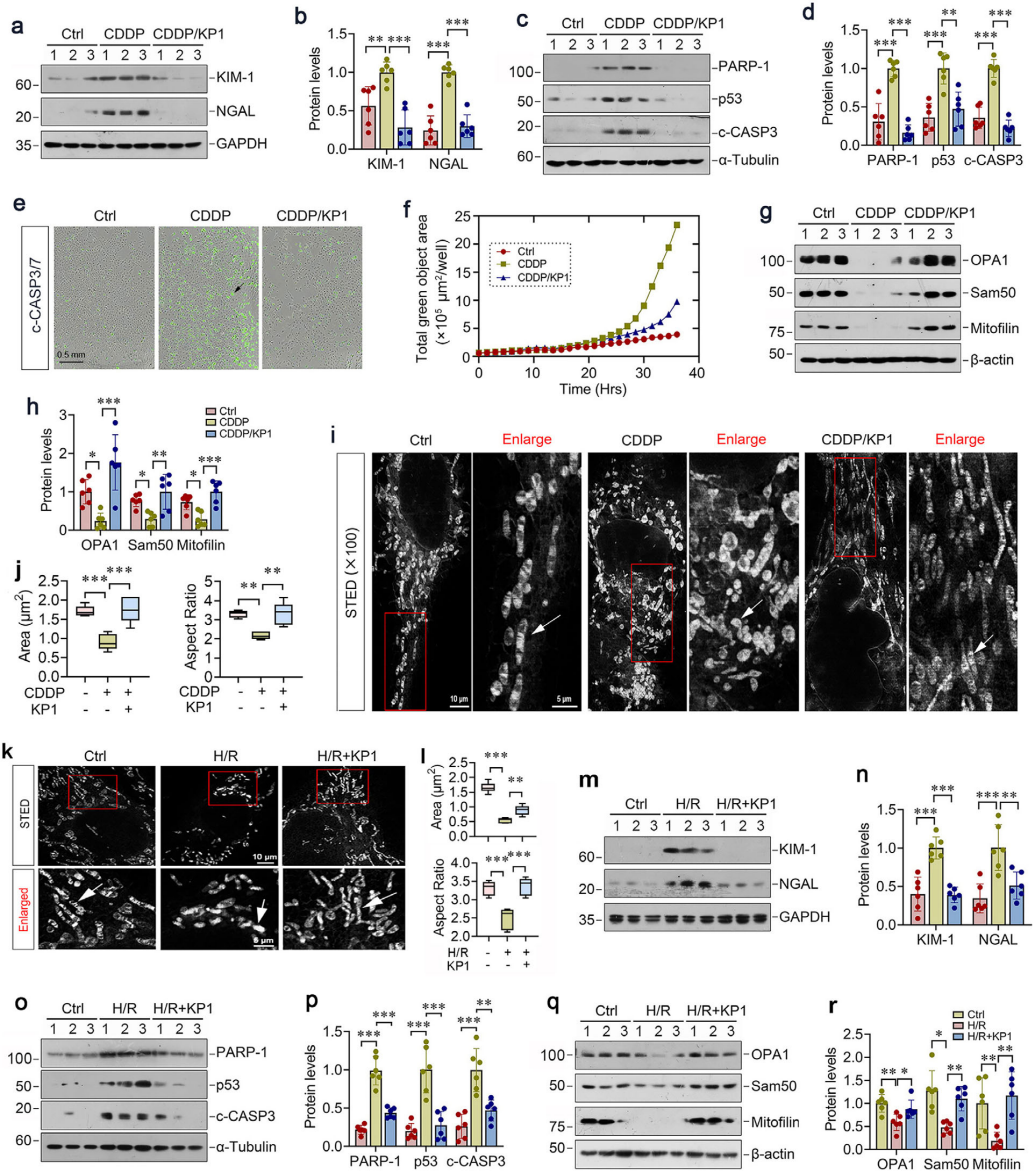
KP1 prevents renal tubular injury and mitochondrial disfunction in vitro. (a,b) Representative Western blot (a) and quantitative data (b) showed that KP1 abolished the induction of KIM‐1 and NGAL proteins in CDDP‐stimulated HKC‐8 cells with KP1 treatment. (c,d) Representative Western blot (c) and quantitative data (d) showed that KP1 abolished the induction of PARP‐1, p53 and c‐CASP3 proteins in CDDP‐stimulated cells with KP1 treatment. (e) Representative high‐throughput live‐cell images showed the positive staining for cleaved caspase‐3/7 in CDDP‐stimulated cells with KP1 treatment at 24 h. Scale bar, 0.5 mm. (f) Graph showed the intensity of CASP3/7‐positive green fluorescence signals over time (µm^2^/well). (g,h) Representative Western blot (g) and quantitative data (h) showed that KP1 restored OPA1, Sam50 and mitofilin protein levels in CDDP‐stimulated HKC‐8 cells with KP1 treatment. (i) Representative stimulated emission depletion (STED) micrographs showed the positive staining for mitochondria in CDDP‐stimulated cells with KP1 treatment. (j) Quantitative data of mitochondrial average area and length‐to‐width ratio (aspect ratio) in CDDP‐stimulated cells with KP1 treatment. (k) Representative STED micrographs showed the positive staining for mitochondria in H/R‐stimulated cells with KP1 treatment. (l) Quantitative data of mitochondrial average area and aspect ratio in H/R‐stimulated cells with KP1 treatment. (m,n) Representative Western blot (m) and quantitative data (n) showed that KP1 abolished the induction of KIM‐1 and NGAL proteins in H/R‐stimulated cells with KP1 treatment. (o,p) Representative Western blot (o) and quantitative data (p) showed that KP1 abolished the induction of PARP‐1, p53 and c‐CASP3 proteins in H/R‐stimulated cells with KP1 treatment. (q,r) Representative Western blot (q) and quantitative data (r) showed that KP1 restored OPA1, Sam50 and mitofilin protein levels in H/R‐stimulated cells with KP1 treatment. ^*^ *p* < 0.05, ^**^ *p* < 0.01, ^***^ *p* < 0.001 (n = 6). ns, no statistical difference.

We next investigated the role of KP1 in protecting tubular cells against nephrotoxic and ischemic injury using cultured human kidney proximal tubular epithelial cells (HKC‐8) in vitro. As shown in Figure 2a–d, CDDP induced the upregulation of tubular injury markers KIM‐1 and NGAL, apoptosis mediators PARP‐1, p53, and c‐CASP3 in HKC‐8 cells, all of which were attenuated by KP1. Longitudinal high‐throughput live‐cell imaging also revealed that KP1 time‐dependently suppressed CDDP‐triggered caspase‐3 and caspase‐7 cleavage and activation after 24 h of incubation (Figure 2e,f). Furthermore, KP1 restored mitochondrial proteins OPA1, Sam50 and mitofilin in CDDP‐treated HKC‐8 cells (Figure 2g,h). Stimulated emission depletion (STED) microscopy provided nanoscale‐resolution imaging of mitochondria, revealing elongated mitochondria with well‐defined cristae in live tubular epithelial cells. However, CDDP induced dramatic transformation of mitochondrial structure, with a smaller round shape due to increased fission and disorganized cristae (Figure 2i). Quantitative analysis showed that CDDP reduced mitochondrial size and the length‐to‐width ratio (aspect ratio) (Figure 2j), while KP1 could restore all of these fine structures of mitochondria in live tubular epithelial cells (Figure 2i,j).

We also assessed the role of KP1 in protecting tubular cells against hypoxic injury by using a hypoxia/reoxygenation (H/R) protocol, an in vitro model that mimics IRI in vivo. As shown in Figure 2k,l, KP1 reversed the ultrastructural changes of mitochondria triggered by H/R, alleviating mitochondrial spherical transformation and cristae disorganization. Consistently, KP1 also inhibited H/R‐induced upregulation of KIM‐1, NGAL, PARP‐1, p53, and c‐CASP3, and preserved mitochondrial integrity by restoring H/R‐depleted OPA1, Sam50, and mitofilin in HKC‐8 cells (Figure 2m–r).

### 2.3 KP1 Elicits Its Protection by Entering Tubular Cells via Endocytosis

KP1 has been reported to interact with TβR2 and inhibit its downstream signaling in CKD. However, we found that CDDP and IRI did not alter p‐Smad3/Smad3 or TβR2 levels, which were also unaffected by KP1 (Figure S3a–d). These results suggest that KP1‐mediated renoprotection following AKI is independent of regulating TGF‐β/Smad3 signaling. Notably, KP1 treatment largely restored endogenous Klotho expression (Figure S3). However, it remained unclear whether it is a direct effect of KP1 on Klotho expression or a consequence of improved pathology in vivo.

**FIGURE 3:**
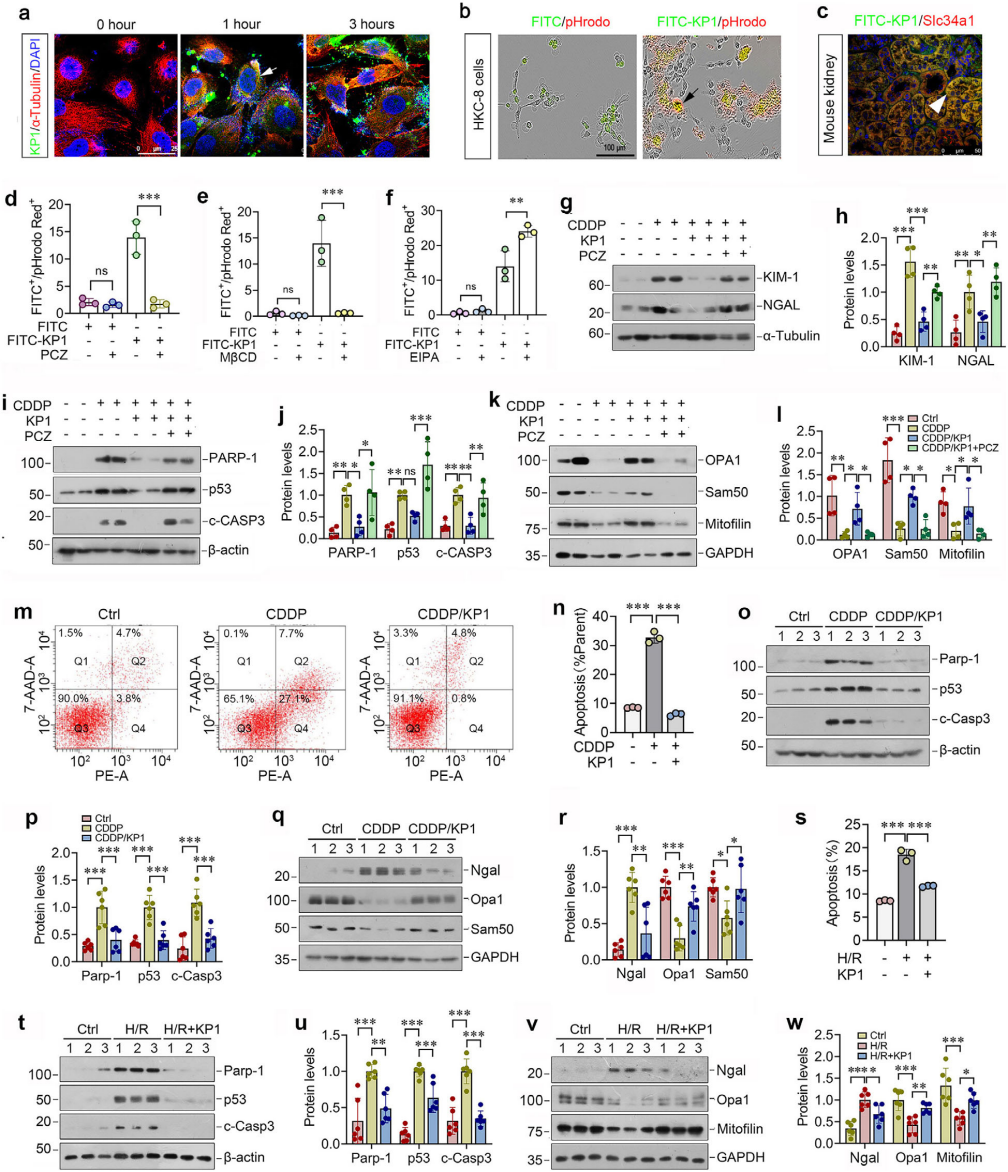
KP1 elicits its protection by entering tubular cells via endocytosis. (a) Representative micrographs of FITC‐KP1‐treated HKC‐8 cells for 0, 1, 3 h. Scale bar, 25 µm. (b) Representative micrographs showed the double‐positive staining for FITC and pHrodo Red in FITC‐ or FITC‐KP1‐treated HKC‐8 cells. Scale bar, 100 µm. (c) Representative immunofluorescence micrographs showed the uptake of FITC‐KP1 by Slc34a1‐positive proximal tubular cells in mouse kidney. Scale bar, 50 µm. (d‐f) Flow cytometry plots analyzing FITC^+^/pHrodo Red^+^ double‐positive cell populations after prochlorperazine (PCZ) (d), methyl‐β‐cyclodextrin (MβCD) (e) and 5‐(N‐Ethyl‐N‐isopropyl)amiloride (EIPA) (f) treatments as indicated. (g‐l) Western blotting analysis for (g,h) KIM‐1 and NGAL, (i,j) PARP‐1, p53 and c‐CASP3, (k,l) OPA1, Sam50 and mitofilin protein levels in HKC‐8 cells after various treatments as indicated. (m) Representative flow cytometry scatter plots in CDDP‐stimulated mouse primary proximal tubular epithelial cells with KP1 treatment for 7‐AAD and PE staining. (n) Quantification of apoptotic rate by flow cytometry. (o‐r) Western blotting analysis for (o,p) Parp‐1, p53 and c‐Casp3, (q,r) Ngal, Opa1 and Sam50 protein levels in CDDP‐stimulated mouse primary proximal tubular epithelial cells with KP1 treatment. (s) Flow cytometry plots analyzing apoptotic rate in H/R‐stimulated mouse primary proximal tubular epithelial cells with KP1 treatment. (t‐w) Western blotting analysis for (t,u) Parp‐1, p53 and c‐Casp3, (v,w) Ngal, Opa1 and mitofilin protein levels in H/R‐stimulated mouse primary proximal tubular epithelial cells with KP1 treatment. ^*^ *p* < 0.05, ^**^ *p* < 0.01, ^***^ *p* < 0.001. ns, no statistical difference.

To elucidate the underlying mechanisms by which KP1 protects against AKI, we first sought to investigate how KP1 elicits its activity by crossing the plasma membrane. To this end, we incubated HKC‐8 cells with FITC‐labeled KP1 to trace its localization, and observed time‐dependent cytoplasmic accumulation of FITC‐KP1 in tubular epithelial cells (Figure 3a). Using longitudinal live‐cell imaging with pHrodo‐Red Dextran, we confirmed FITC‐KP1 co‐localization with endocytic vesicles at 3 h, whereas co‐staining of unconjugated FITC with pHrodo‐Red was negligible (Figure 3b), indicating endocytosis‐dependent uptake of KP1 by HKC‐8 cells. Tubular cell uptake of FITC‐KP1 was also evidenced in mouse kidney in vivo after intravenous injection, as it co‐localized with proximal tubular marker Slc34a1 (Figure 3c). Furthermore, incubation with clathrin‐mediated endocytosis inhibitor prochlorperazine (PCZ) or caveolae‐mediated endocytosis inhibitor methyl‐β‐cyclodextrin (MβCD) blocked FITC‐KP1 entry to HKC‐8 cells, as assessed by flow cytometry (Figure 3d,e). However, incubation with macropinocytosis inhibitor 5‐(N‐Ethyl‐N‐isopropyl)amiloride (EIPA) did not block FITC‐KP1 entry to HKC‐8 cells (Figure 3f). These results suggest that the cellular uptake of KP1 is primarily mediated by endocytosis but not by macropinocytosis.

We found that blockade of KP1 uptake by PCZ abolished the cytoprotective activity of KP1 in HKC‐8 cells following CDDP treatment. In the presence of PCZ, KP1 was unable to inhibit KIM‐1, NGAL, PARP‐1, p53, and c‐CASP3 induction by CDDP (Figure 3g–j). Similarly, blockade of KP1 uptake by PCZ also abolished the ability of KP1 to restore OPA1, Sam50, and mitofilin in HKC‐8 cells after CDDP treatment (Figure 3k,l). These results indicate that the renoprotective activity of KP1 is dependent on its entry into tubular epithelial cells.

To closely mimic the in vivo situation, we utilized primary mouse proximal tubular epithelial cells, characterized by E‐cadherin^+^/vimentin^−^ immunostaining (Figure S4), to assess the ability of KP1 to protect tubular cells against nephrotoxic or hypoxic injury. As shown in Figure 3m,n, KP1 suppressed CDDP‐induced apoptosis in mouse primary proximal tubular epithelial cells, as assessed by flow cytometry. KP1 also repressed the expression of Parp‐1, p53, and c‐Casp3 (Figure 3o,p), inhibited Ngal, and restored Opa1 and Sam50 in mouse primary tubular cells (Figure 3q,r). Similar results were obtained when these cells were challenged with H/R. KP1 was able to reduce cell apoptosis (Figure 3s), inhibited Parp‐1, p53, c‐Casp3 and Ngal expression, and restored Opa1 and mitofilin after H/R treatment (Figure 3t–w).

### 2.4 KP1 Directly Binds Mitochondrial Protein ATAD3A

**FIGURE 4:**
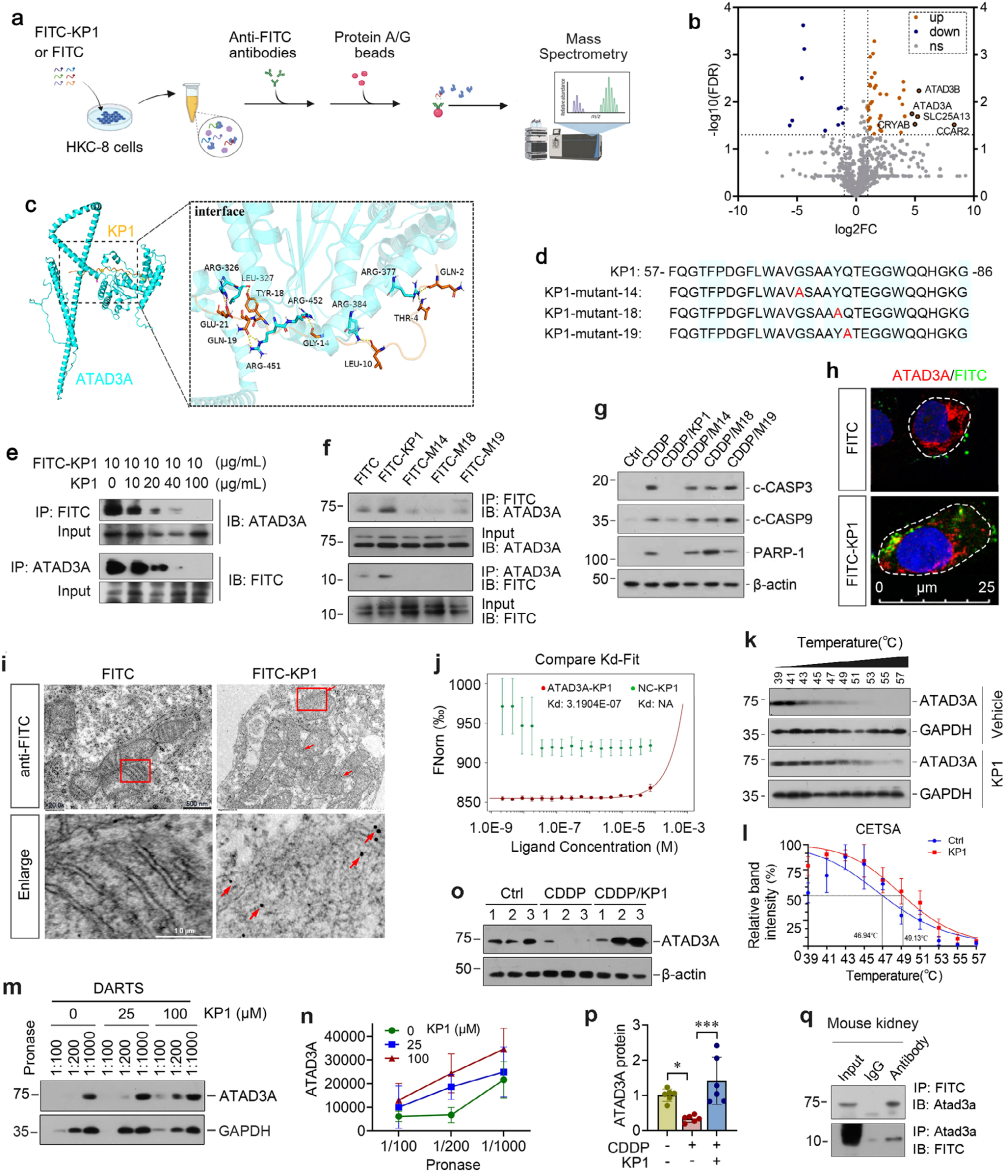
KP1 directly binds mitochondrial protein ATAD3A. (a) Experimental workflow diagram for identifying KP1‐targeted proteins using co‐immunoprecipitation‐mass spectrometry (Co‐IP/MS). (b) Volcano plots of the differentially pull‐down proteins in HKC‐8 cells of FITC versus FITC‐KP1 (n = 3 biologically independent samples). Top 5 KP1‐interacting proteins are indicated. (c) Molecular docking simulation of KP1 with ATAD3A, with KP1 in orange and ATAD3A in blue. Key amino acid residues at the binding interface are labeled. (d) KP1 and its mutant sequences, with blue background indicating conserved sequences through evolution and mutated residues highlighted in red. (e) Competitive binding assay with unlabeled KP1 competing with FITC‐KP1 for ATAD3A binding. HKC‐8 cell lysates were incubated with increasing concentrations of KP1 (0, 10, 20, 40, 100 µg/mL) in combination with a fixed concentration of FITC‐KP1 (10 µg/mL), followed by immunoprecipitation with anti‐FITC antibody and immunoblotting for ATAD3A, whereas reciprocal Co‐IP was performed with anti‐ATAD3A antibody and immunoblotting for FITC. (f) Co‐IP demonstrated that KP1 bound to ATAD3A. The HKC‐8 cell lysates incubated with FITC, FITC‐KP1, FITC‐KP1‐M14, FITC‐KP1‐M18, and FITC‐KP1‐M19 were immunoprecipitated (IP) with the anti‐FITC antibody at 4 °C overnight, followed by immunoblotted (IB) for ATAD3A, whereas in the reciprocal experiment, mixtures were IP with anti‐ATAD3A, followed by IB for FITC. (g) Representative Western blot analyses of c‐CASP3, c‐CASP9 and PARP‐1 in CDDP‐stimulated HKC‐8 cells treated with KP1 or KP1 mutants. (h) Representative immunofluorescence micrographs of ATAD3A in HKC‐8 cells after treatment with FITC or FITC‐KP1. Scale bar, 25 µm. (i) Representative immunogold electron micrographs demonstrating mitochondrial membrane localization of KP1. HKC‐8 cells were treated with FITC or FITC‐KP1, and arrowheads indicate FITC‐KP1‐positive signals. (j) MST binding curves of KP1‐ATAD3A interaction. X‐axis represents ligand (KP1) concentration (M), while Y‐axis represents normalized fluorescence intensity. Red curve: KP1 titration with lysate from 293T cells overexpressing ATAD3A‐GFP fusion protein. Green curve: KP1 titration with lysate from 293T cells overexpressing GFP protein. Calculated *Kd* = 3.19 × 10^−7^ M. (k) Western blot results of cellular thermal shift assay (CETSA). HKC‐8 cells were treated with KP1 or vehicle, followed by gradient temperature incubation. ATAD3A protein levels were detected by immunoblotting, with GAPDH as the control. (l) Melting curves of ATAD3A derived from CETSA assay. The relative band intensity was plotted against temperature, and the melting temperature (Tm) was calculated. (m,n) Drug affinity responsive target stability (DARTS) analysis of ATAD3A‐KP1 interaction. Cell lysates were incubated with KP1 (0, 25, or 100 µM) followed by digestion with Pronase at 1:100, 1:200, and 1:1000 dilutions. ATAD3A protein levels were assessed by immunoblotting (m). Quantification of band intensity (n): ATAD3A degradation rates decreased dose‐dependently with KP1. (o, p) Western blotting analysis for ATAD3A protein level in HKC‐8 cells after treatment with CDDP in the absence or presence of KP1. ^*^ *p* < 0.05, ^***^ *p* < 0.001 (n = 6). (q) Co‐IP assay of mouse kidney showed that human FITC‐KP1 could bind mouse Atad3a in vivo. Kidney lysates were immunoprecipitated with anti‐FITC antibody, followed by immunoblotting for Atad3a, whereas reciprocal IP was performed with anti‐Atad3a, followed by immunoblotting for FITC. IgG served as the negative control.

To identify intracellular targets of KP1, we employed a co‐immunoprecipitation coupled with mass spectrometry (Co‐IP/MS) approach. HKC‐8 cells were incubated with FITC or FITC‐KP1, and immunoprecipitation was performed using anti‐FITC‐conjugated agarose beads, followed by mass spectrometry analysis (Figure 4a). Co‐IP/MS revealed that KP1 interacted with multiple mitochondrial proteins. Among them, the most notable one was ATAD3A (Figure 4b). Molecular docking simulation revealed that KP1 exhibited high affinity for ATAD3A, with G14, Y18, and Q19 as contact sites, corresponding to Gly70, Tyr74, and Gln75 in the human αKlotho protein, respectively (Figure 4c,d). Furthermore, Co‐IP analysis demonstrated that FITC‐tagged KP1 physically interacted with ATAD3A in HKC‐8 cells (Figure 4e), validating the binding of KP1 to ATAD3A. A competition assay with excess unlabeled KP1 validated the specificity of the FITC‐KP1/ATAD3A interaction (Figure 4e). Point mutations of KP1 by substituting alanine at the predicted binding residues G14, Y18, and Q19 abolished the KP1/ATAD3A interaction in Co‐IP assays (Figure 4f). Consistently, single‐amino acid mutations at these binding sites abolished the cytoprotective effect of KP1, as the M14, M18, and M19 mutants failed to prevent CDDP‐induced injury (Figure 4g and Figure S5). Confocal microscopic imaging confirmed the intracellular co‐localization of KP1 and ATAD3A (Figure 4h). To further ascertain the mitochondrial localization of KP1, we employed immunogold electron microscopy to localize FITC‐KP1 and visualized its presence on mitochondrial membranes in HKC‐8 cells (Figure 4i). Microscale thermophoresis (MST), an immobilization‐free fluorescence‐based biophysical technique for analyzing biomolecular interactions [21], confirmed high‐affinity binding between KP1 and ATAD3A, with a Kd of 3.19×10^−7^ M (Figure 4j). Furthermore, we employed the cellular thermal shift assay (CETSA), a label‐free biophysical technique based on the principle of ligand‐induced thermal stabilization of target proteins inside intact cells [22], and demonstrated that KP1 caused a shift in the melting temperature curve of ATAD3A, but not that of the control protein GAPDH (Figure 4k,l).

To investigate the functional consequence of KP1 binding to ATAD3A, we utilized the drug affinity responsive target stability (DARTS) assays. As shown in Figure 4m,n, KP1 dose‐dependently protected ATAD3A from pronase‐mediated degradation, indicating that KP1 stabilizes its target protein ATAD3A upon binding. We found that in mouse models of AKI induced by CDDP or IRI, KP1 treatment completely prevented injury‐induced ATAD3A depletion (Figure S6a–e). Furthermore, tubular cell injury triggered by CDDP or H/R caused depletion of ATAD3A protein in HKC‐8 cells in vitro, which was reversed by KP1 incubation (Figure 4o,p and Figure S6f). Of note, human KP1 shares 96% identity with its mouse counterpart [19] and was able to interact with Atad3a in mouse kidney after injection of FITC‐KP1 (Figure 4q). Collectively, these results indicate that KP1 directly binds to mitochondrial ATAD3A and stabilizes it by preventing injury‐induced degradation. Interestingly, incubation of HKC‐8 cells with His‐tagged soluble Koltho (sKlotho) revealed that Klotho protein neither entered tubular cells nor interacted with ATAD3A (Figure S7).

### 2.5 ATAD3A Preserves Mitochondrial Integrity and Protects Against AKI in Vivo

**FIGURE 5:**
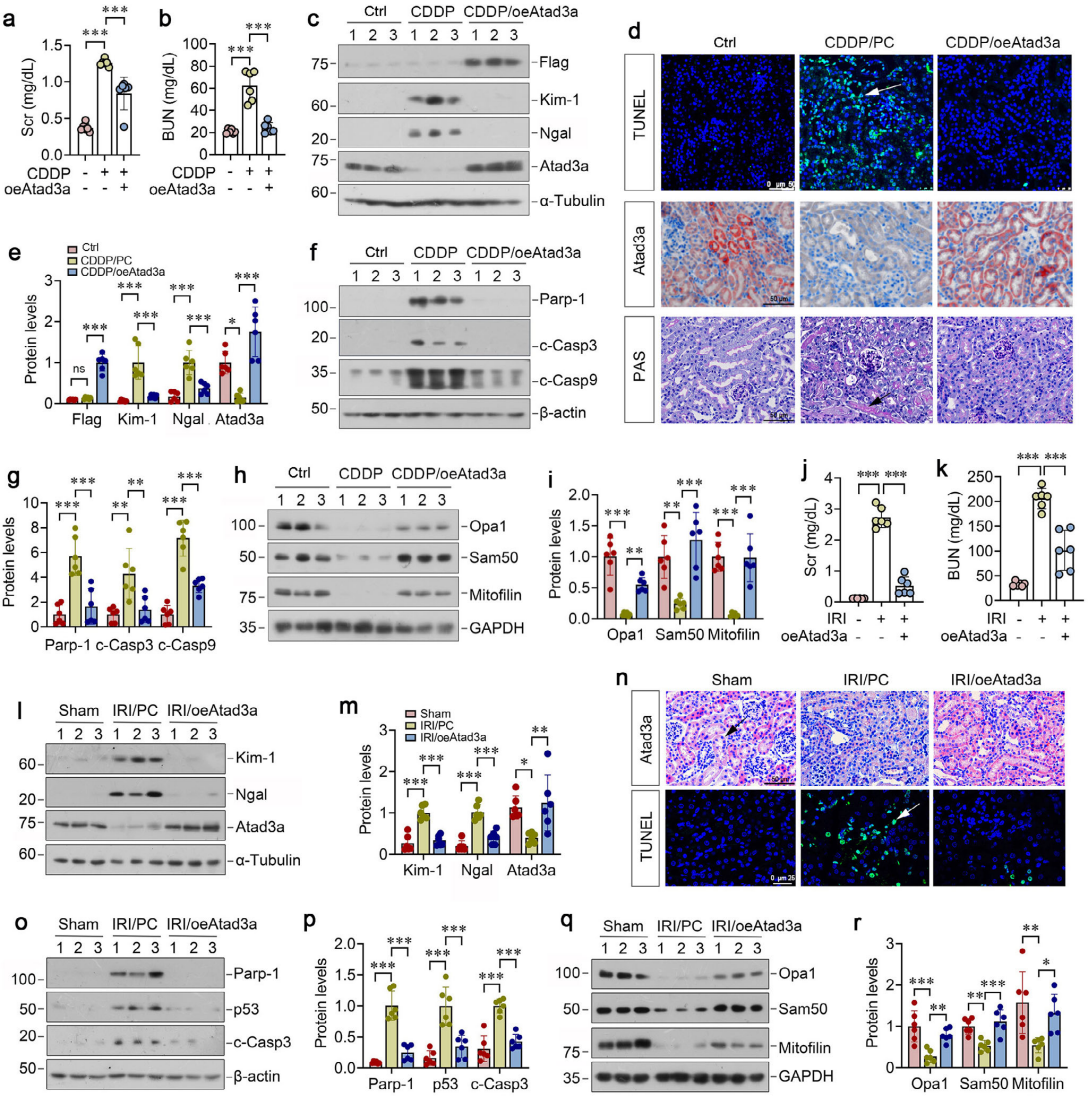
ATAD3A preserves mitochondrial functions and protects against AKI in vivo. (a,b) Graphic presentation shows the Scr and BUN levels in CDDP mice with Atad3a overexpression. (c) Representative Western blot analyses of Flag, Kim‐1, Ngal and Atad3a proteins in CDDP mice after injection with Atad3a‐overexpression plasmids. (d) Representative immunostaining for Atad3a and PAS and TUNEL staining in CDDP mice with Atad3a overexpression. (e) Quantitative data of Flag, Kim‐1, Ngal and Atad3a protein levels. (f‐i) Western blotting analysis for (f,g) Parp‐1, c‐Casp3 and c‐Casp9, (h, i) Opa1, Sam50 and mitofilin protein levels in CDDP mice with Atad3a overexpression. (j,k) Graphic presentation shows the Scr and BUN levels in IRI mice with Atad3a overexpression. (l,m) Western blotting analysis for Kim‐1, Ngal and Atad3a. (n) Representative immunostaining for Atad3a and TUNEL staining in IRI mice with Atad3a overexpression. (o‐r) Western blotting analysis for (o,p) Parp‐1, p53 and c‐Casp3, (q,r) Opa1, Sam50 and mitofilin protein levels in IRI mice with Atad3a overexpression. ^*^ *p* < 0.05, ^**^ *p* < 0.01, ^***^ *p* < 0.001. ns, no statistical difference.

To explore the role of Atad3a in regulating AKI, we manipulated its expression level in mice via overexpression of the exogenous *Atad3a* gene. To this end, mice were injected with an expression plasmid encoding Flag‐tagged *Atad3a* (pFlag‐*Atad3a*) using a hydrodynamics‐based gene transfer approach 2 days before CDDP injection. As shown in Figure 5a,b, overexpression of exogenous Atad3a reduced Scr and BUN levels and mitigated kidney dysfunction. Notably, the expression of exogenous Atad3a was confirmed by immunoblotting for Flag and Atad3a in kidney lysates (Figure 5c–e). Immunohistochemical staining also demonstrated that Atad3a expression was completely restored in renal tubular epithelium in mice injected with the pFlag‐*Atad3a* plasmid (Figure 5d). Overexpression of exogenous Atad3a also suppressed the expression of Kim‐1, Ngal (Figure 5c, e), and Parp‐1, c‐Casp3, and c‐Casp9 (Figure 5f,g). TUNEL staining and histopathological assessment revealed a decrease in TUNEL^+^ cells, accompanied by reduced tubular dilation and cast deposition after Atad3a overexpression (Figure 5d). Atad3a also preserved Opa1, Sam50 and mitofilin in the kidney after CDDP treatment (Figure 5h,i).

We also investigated the effect of exogenous Atad3a on AKI induced by IRI. As shown in Figure 5j–p, overexpression of Atad3a decreased Scr and BUN levels, inhibited the expression of Kim‐1, Ngal, Parp‐1, p53 and c‐Casp3, and reduced TUNEL^+^ apoptotic cells in the kidneys of mice subjected to IRI. Atad3a overexpression also largely preserved Opa1, Sam50 and mitofilin, underscoring its role in protecting mitochondrial integrity (Figure 5q,r). In contrast, knockdown of *Atad3a* expression by short hairpin RNA (shRNA)‐mediated inhibition further worsened AKI after IRI, with elevated Scr and BUN levels (Figure S8a,b). Knockdown of Atad3a also aggravated renal expression of Kim‐1, Ngal, Parp‐1, p53 and c‐Casp3 after IRI (Figure S8c–f). Importantly, Atad3a depletion further decreased Opa1, Sam50 and mitofilin, and worsened histological injury (Figure S8g–i).

### 2.6 ATAD3A Preserves Mitochondrial Integrity in Tubular Cells After Injury in Vitro

**FIGURE 6:**
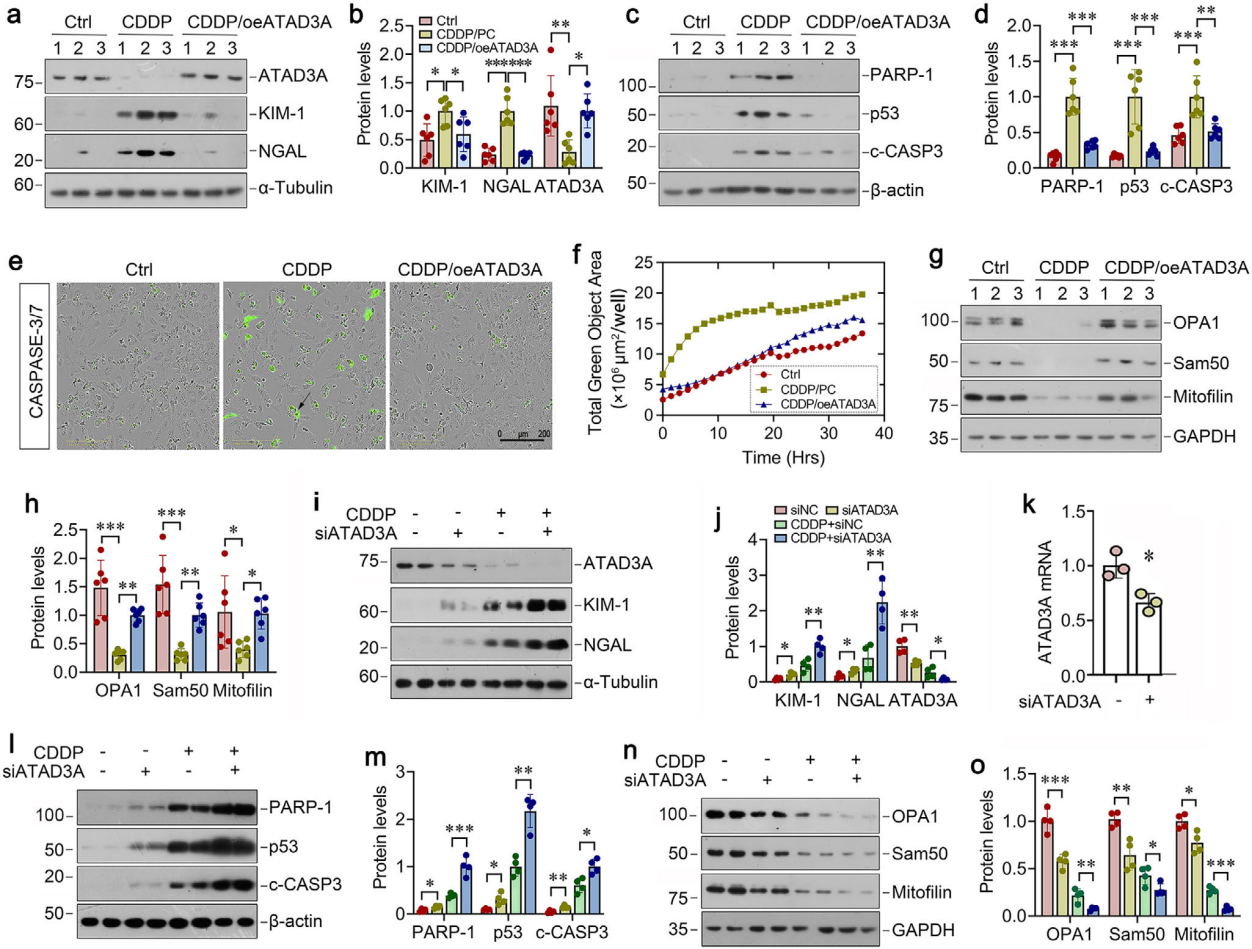
ATAD3A alleviates CDDP‐induced mitochondrial dysfunction in vitro. (a‐d) Western blotting analysis for (a,b) KIM‐1, NGAL and ATAD3A, (c,d) PARP‐1, p53 and c‐CASP3 protein levels in CDDP‐stimulated cells with ATAD3A overexpression. (e) Representative CASP3/7 staining images in ATAD3A‐overexpression CDDP‐stimulated cells. Scale bar, 200 µm. (f) Time‑course of the intensity of CASP3/7‐positive green fluorescence signals (µm^2^/well). (g,h) Western blotting analysis for OPA1, Sam50, mitofilin protein levels in CDDP‐stimulated cells with ATAD3A overexpression. (i) Representative Western blot of KIM‐1, NGAL and ATAD3A proteins in HKC‐8 cells after treatment as indicated. (j) Quantitative data showed the levels of KIM‐1, NGAL and ATAD3A proteins. (k) Knockdown of ATAD3A expression by siRNA in HKC‐8 cells. The ATAD3A mRNA levels were assessed by qRT‐PCR in different groups as indicated. (l‐o) Western blotting analysis for (l,m) PARP‐1, p53 and c‐CASP3, (n,o) OPA1, Sam50 and mitofilin protein levels in HKC‐8 cells after various treatments as indicated. ^*^ *p* < 0.05, ^**^ *p* < 0.01, ^***^ *p* < 0.001. ns, no statistical difference.

We further investigated the role of ATAD3A in protecting against mitochondrial dysfunction after injury by using HKC‐8 cells in vitro. As shown in Figure 6a,b, overexpressing ATAD3A restored its protein levels and suppressed KIM‐1 and NGAL after CDDP treatment. Overexpression of ATAD3A also prevented CDDP‐induced upregulation of PARP‐1, p53, and c‐CASP3 expression (Figure 6c,d). Live‐cell imaging confirmed that ATAD3A overexpression inhibited CDDP‐triggered activation of caspase‐3 and caspase‐7 (Figure 6e,f). The protective effect of ATAD3A was associated with the restoration of OPA1, Sam50 and mitofilin (Figure 6g,h), suggesting that ATAD3A alone is sufficient for protecting against CDDP‐induced mitochondrial dysfunction and apoptosis.

We then confirmed the role of ATAD3A in preserving mitochondrial integrity by knocking down its expression using small interfering RNA (siRNA). As shown in Figure 6i–m, knockdown of ATAD3A by si*ATAD3A* in HKC‐8 cells slightly induced KIM‐1, NGAL, PARP‐1, p53, and c‐CASP3 expression, suggesting that ATAD3A depletion spontaneously triggers tubular injury and apoptosis. Moreover, knockdown of ATAD3A further exacerbated CDDP‐triggered induction of these proteins (Figure 6i–m). ATAD3A loss also aggravated mitochondrial dysfunction, as evidenced by decreased OPA1, Sam50, and mitofilin (Figure 6n,o).

We also studied the effect of ATAD3A on tubular cell injury induced by H/R. As shown in Figure S9a–d, overexpression of ATAD3A reduced the expression of KIM‐1, NGAL, PARP‐1, p53, and c‐CASP3 in HKC‐8 cells. Overexpression of ATAD3A also restored OPA1, Sam50, and mitofilin, which were suppressed by H/R treatment (Figure S9e,f). Conversely, knockdown of ATAD3A by the siRNA approach abolished the cytoprotective effects of KP1 after H/R injury (Figure S9g–j). Knockdown of ATAD3A also prevented the restoration of mitochondrial proteins by KP1 after H/R treatment (Figure S9k,l). These results indicate that ATAD3A is a critical downstream mediator of KP1 in protecting against tubular injury and mitochondrial dysfunction in AKI.

### 2.7 ATAD3A Preserves Mitochondrial Integrity Through Direct Interaction With HIGD2A

**FIGURE 7:**
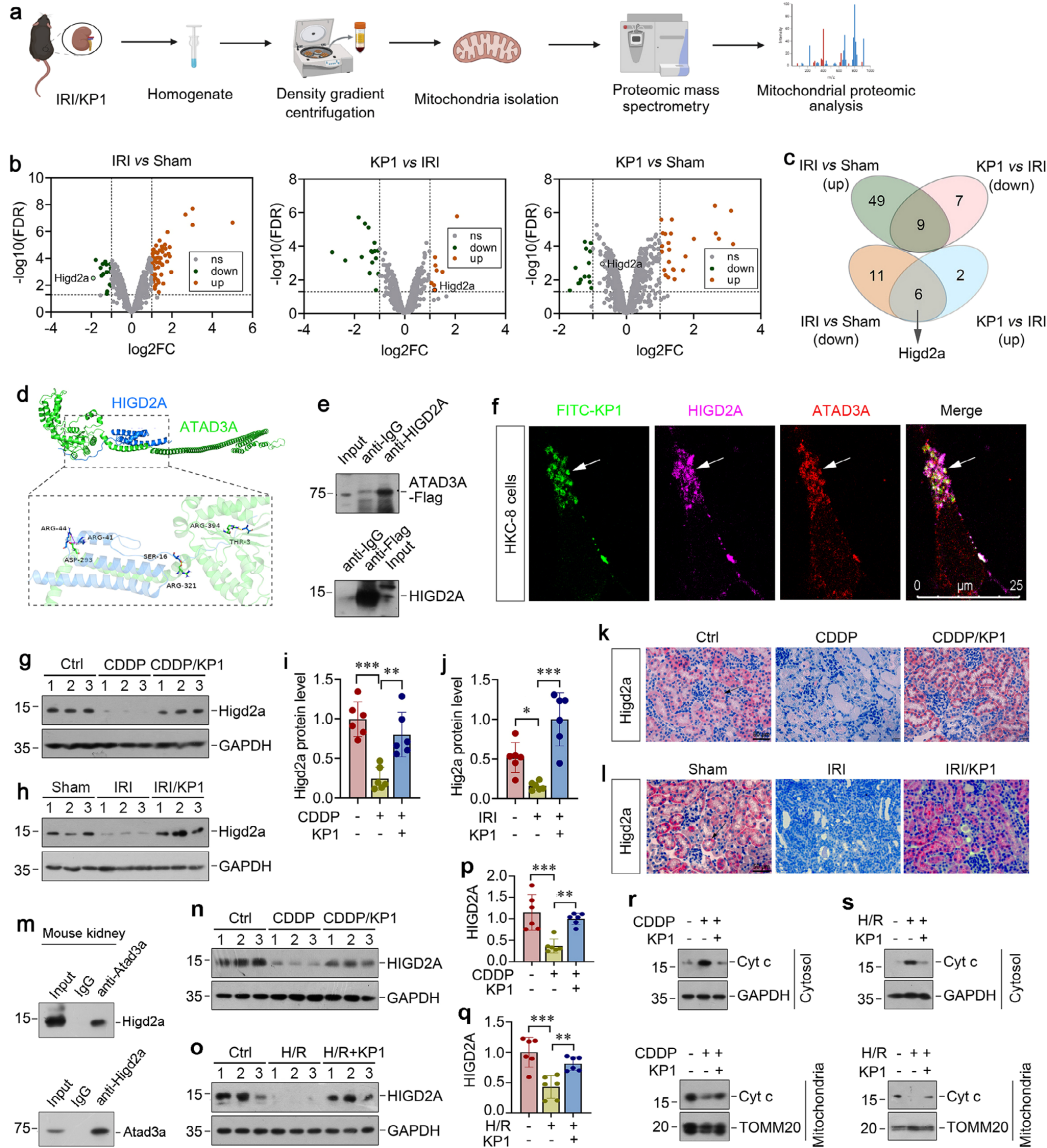
ATAD3A preserves mitochondrial integrity through direct interaction with HIGD2A. (a) Schematic workflow of mitochondrial proteomic analysis. Kidneys from sham, IRI or IRI/KP1 mice were homogenized, processed by density gradient centrifugation to isolate mitochondria, and then subjected to mass spectrometry for mitochondrial proteomic analysis. (b) Volcano plots of the differentially expressed proteins in the kidney mitochondria of different groups as indicated, including ‘IRI versus Sham’, ‘IRI+KP1 versus IRI’, and ‘IRI+KP1 versus Sham’ (n = 3 biologically independent animals). (c) Venn diagram showed the differential expression of mitochondrial proteins in the kidneys of different groups, presenting the overlap of upregulated and downregulated proteins across comparisons. (d) Molecular docking simulation of ATAD3A with HIGD2A, with ATAD3A in green and HIGD2A in blue. (e) Co‐IP demonstrated that HIGD2A bound to ATAD3A. *ATAD3A*‐Flag‐overexpressing HKC‐8 cell lysates  were IP with the anti‐HIGD2A antibody, followed by IB for ATAD3A. In the reciprocal experiment, mixtures were IP with anti‐Flag antibody, followed by IB for HIGD2A. (f) Representative immunofluorescence micrographs showed co‐localization of ATAD3A (red) and HIGD2A (purple) in FITC‐tagged (green)‐KP1‐treated HKC‐8 cells. Scale bar, 25 µm. (g,h) Representative Western blot for Higd2a protein in (g) CDDP mice and (h) IRI mice with KP1 treatment. (i,j) Quantitative data of Higd2a protein level in (i) CDDP mice and (j) IRI mice with KP1 treatment. (k,l) Representative immunostaining for Higd2a in (k) CDDP mice and (l) IRI mice with KP1 treatment. (m) In vivo Co‐IP assay confirmed the interaction between endogenous Atad3a and Higd2a in mice. Kidney lysates were immunoprecipitated with anti‐Atad3a or anti‐Higd2a antibody respectively, followed by immunoblotting for the corresponding binding partner. IgG was used as the isotype control. (n, o) Representative Western blot for HIGD2A protein expression in (n) CDDP‐ and (o) H/R‐treated HKC‐8 cells in the absence or presence of KP1. (p,q) Quantitative data of HIGD2A protein levels in (p) CDDP‐ and (q) H/R‐treated HKC‐8 cells without or with KP1 treatment. (r) Representative Western blot of cytosolic and mitochondrial cytochrome c (Cyt c) protein in KP1‐treated HKC‐8 cells subjected to CDDP treatment. (s) Representative Western blot of cytosolic and mitochondrial Cyt c protein in KP1‐treated HKC‐8 cells subjected to H/R injury. ^**^ *p* < 0.01, ^***^ *p* < 0.001.

To elucidate the mechanism underlying ATAD3A‐mediated protection against mitochondrial dysfunction, we sought to identify the direct targets of ATAD3A in mitochondria by using an unbiased proteomic approach. Mitochondria were isolated from kidneys of different groups of mice and subjected to mass spectrometry (MS) (Figure 7a). We identified 58 upregulated and 17 downregulated proteins in the IRI group versus sham controls (Figure 7b). Compared to IRI, KP1 treatment resulted in 8 upregulated and 16 downregulated proteins (Figure 7b). Among these, KP1 restored the expression of 6 mitochondrial proteins that were downregulated in IRI kidneys. The top hit was the hypoxia‐inducible gene 1 (HIG1) domain family member 2A (HIGD2A), a subunit of the cytochrome c oxidase complex (complex IV) (Figure 7c). Molecular docking simulation predicted a strong ATAD3A‐HIGD2A interaction, with a binding free energy of ‐115.99 kcal/mol (Figure 7d). To validate the interaction between ATAD3A and HIGD2A, we performed co‐IP assays. After incubation of the cell lysates from HKC‐8 cells overexpressing Flag‐tagged ATAD3A with anti‐HIGD2A antibody, ATAD3A was detected in the immunocomplexes (Figure 7e). In the reciprocal experiments, HIGD2A was also readily detected in the complexes precipitated by anti‐Flag antibody (Figure 7e). Confocal microscopy confirmed the co‐localization of FITC‐KP1, ATAD3A and HIGD2A in HKC‐8 cells (Figure 7f).

We found that AKI caused by either CDDP or IRI resulted in marked downregulation of Higd2a protein, which was restored by KP1 in both models (Figure 7g–j). Immunohistochemical staining also revealed that KP1 could restore Higd2a expression in the tubular epithelium of the kidneys in AKI mice induced by CDDP or IRI (Figure 7k,l). In the mouse kidney, Atad3a and Higd2a were physically associated, as demonstrated by Co‐IP (Figure 7m). Similarly, when HKC‐8 cells were treated with CDDP or H/R, KP1 clearly preserved the expression of HIGD2A protein in vitro (Figure 7n–q). We found that treatment with CDDP or H/R was associated with the release of cytochrome c from mitochondria into the cytosol, and KP1 reversed such cytochrome c translocation (Figure 7r,s), which could trigger tubular cell apoptosis.

### 2.8 HIGD2A Protects Against Renal Tubular Cell Apoptosis

**FIGURE 8:**
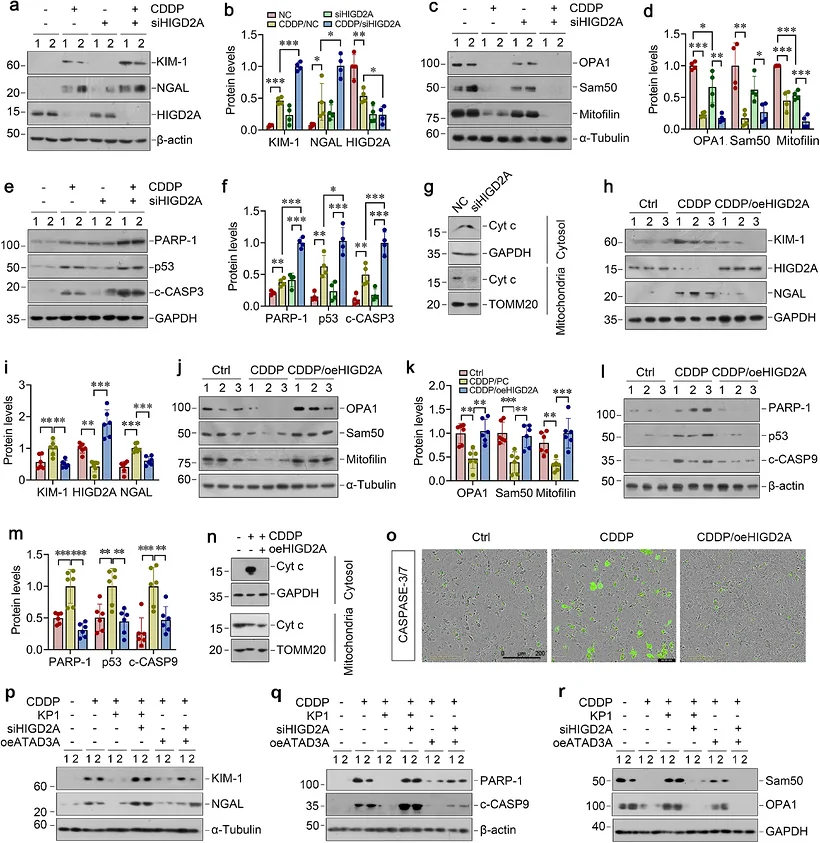
HIGD2A protects against renal tubular cell apoptosis. (a‐f) Western blotting analysis for (a,b) KIM‐1, NGAL and HIGD2A, (c,d) OPA1, Sam50 and mitofilin, (e, f) PARP‐1, p53 and c‐CASP3 protein levels in HKC‐8 cells after treatment as indicated. (g) Representative Western blot of cytosolic and mitochondrial Cyt c protein in HKC‐8 cells after knocking down HIGD2A by siRNA approach. (h‐m) Western blotting analysis for (h,i) KIM‐1, NGAL and HIGD2A, (j,k) OPA1, Sam50 and mitofilin, (l,m) PARP‐1, p53 and c‐CASP9 protein levels. HKC‐8 cells were transfected with HIGD2A expression vector, followed by treatment without or with CDDP. (n) Representative Western blot analysis of cytosolic and mitochondrial Cyt c protein in HKC‐8 cells after various treatment as indicated. (o) Representative CASP3/7 staining images in HKC‐8 cells after various treatment as indicated. (p‐r) Western blotting analysis for (p) KIM‐1 and NGAL, (q) PARP‐1 and c‐CASP9, (r) Sam50 and OPA1 protein levels in HKC‐8 cells after various treatments as indicated. ^*^ *p* < 0.05, ^**^ *p* < 0.01, ^***^ *p* < 0.001. ns, no statistical difference.

To confirm the role of HIGD2A in protecting against tubular cell apoptosis, we manipulated the expression of HIGD2A by knocking down or overexpressing it in HKC‐8 cells in vitro. As shown in Figure 8a,b, knockdown of *HIGD2A* by siRNA aggravated the CDDP‐induced upregulation of KIM‐1 and NGAL expression. HIGD2A depletion also accelerated the loss of OPA1, Sam50, and mitofilin induced by CDDP (Figure 8c,d). Consequently, HIGD2A knockdown aggravated PARP‐1, p53, and c‐CASP3 expression induced by CDDP (Figure 8e,f), suggesting that HIGD2A deficiency further exacerbated tubular cell injury and apoptosis. Mitochondrial/cytosolic fractionation assays revealed that HIGD2A depletion alone promoted mitochondrial cytochrome c release into the cytosol (Figure 8g).

We further examined the role of HIGD2A in protecting against tubular cell apoptosis by overexpression. As shown in Figure 8h–m, overexpression of HIGD2A abolished CDDP‐induced upregulation of KIM‐1, NGAL, PARP‐1, p53 and c‐CASP9 and restored OPA1, Sam50 and mitofilin in CDDP‐treated HKC‐8 cells, suggesting that HIGD2A protects against tubular injury, apoptosis, and mitochondrial dysfunction. Moreover, HIGD2A blocked CDDP‐triggered cytochrome c release from mitochondria to cytosol (Figure 8n), thereby inhibiting downstream caspase‐3 and caspase‐7 activation (Figure 8o).

We found that KP1‐mediated protection of tubular cells depended on HIGD2A expression, as knockdown of *HIGD2A* by siRNA abolished KP1‐mediated inhibition of KIM‐1, NGAL, PARP‐1 and c‐CASP9, and prevented KP1 from restoring Sam50 and OPA1 expression in HKC‐8 cells after CDDP treatment. (Figure 8p–r and Figure S10a–f). Similarly, knockdown of HIGD2A also negated the protective effects of ATAD3A overexpression in tubular cells (Figure 8p–r and Figure S10a–f). Furthermore, KP1 restored ATAD3A and HIGD2A interaction in the presence of CDDP (Figure S10g). Together, these results suggest that HIGD2A is a downstream effector that mediates the cytoprotective action of the KP1/ATAD3A axis.

### 2.9 KP1 Confers Renal Protection in Established AKI

**FIGURE 9:**
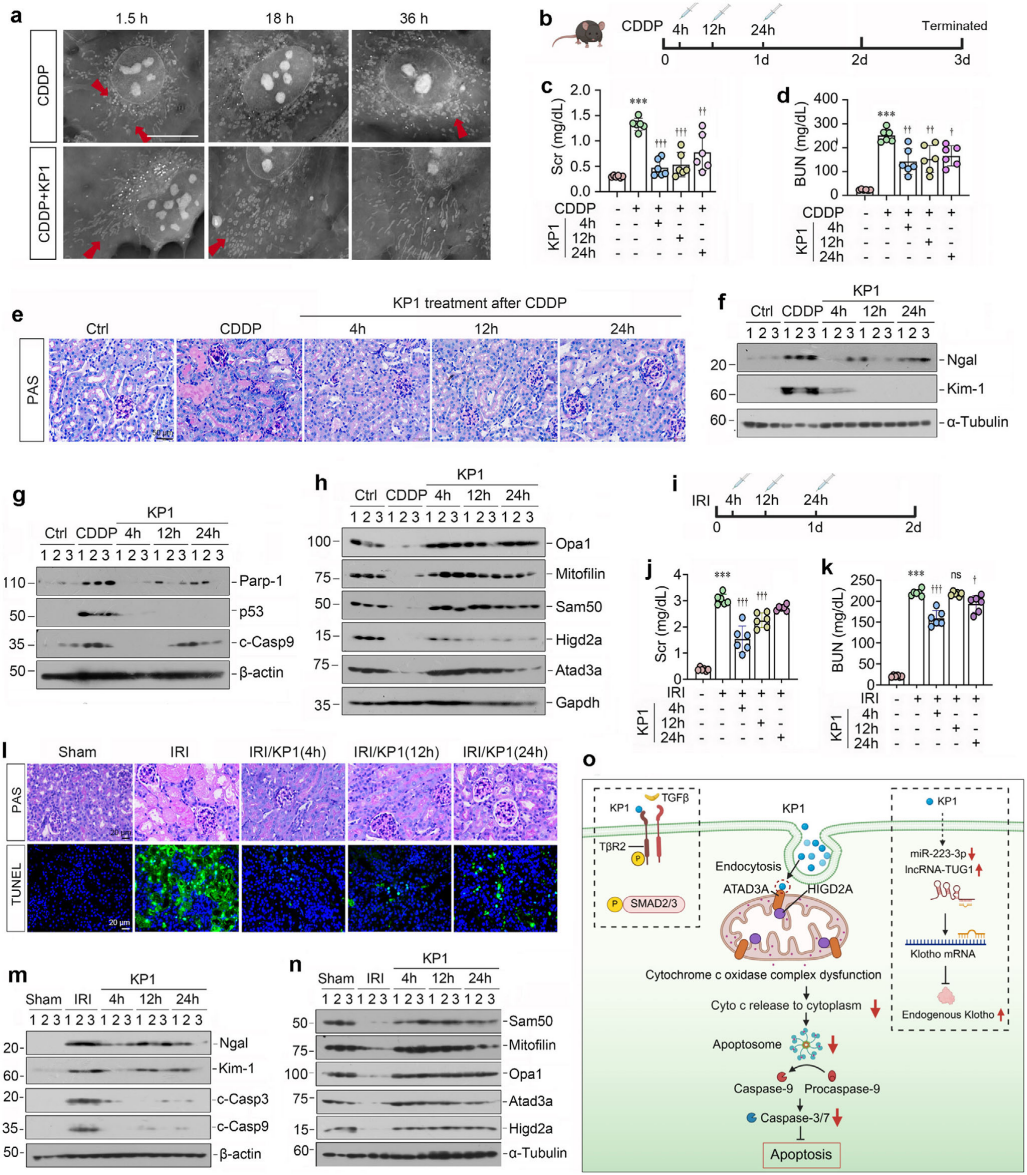
Delayed administration of KP1 protects against both nephrotoxic and ischemic AKI in mice. (a) Representative label‐free live‐cell micrographs showed mitochondrial morphology in HKC‐8 cells at different time points (1.5, 18, 36 h) after treatment with CDDP in the absence or presence of KP1. Scale bar, 20 µm. (b) Schematic diagram showed KP1 treatment protocol in CDDP‐induced AKI. KP1 was administered at 4, 12 and 24 h after CDDP injection, and mice were euthanatized on day 3. (c,d) Graphic presentation showed Scr and BUN levels in CDDP mice with different KP1 treatment protocols. ^***^ *p* < 0.001 *vs* Ctrl. ^†^ *p* < 0.05, ^††^ *p* < 0.01, ^†††^ *p* < 0.001 *vs* CDDP. (e) Representative micrographs of PAS staining in different groups of mice as indicated. (f‐h) Representative Western blot analyses of renal Ngal and Kim‐1 (f), Parp‐1, p53, c‐Casp9 (g) and Opa1, Sam50, Mitofilin, Higd2a, Atad3a (h) proteins in different groups as indicated. (i) Schematic diagram of KP1 treatment protocol in IRI‐induced AKI. KP1 was administered at 4, 12 and 24 h after IRI, and mice were euthanatized on day 2. (j, k) Graphic presentation showed Scr and BUN levels in different groups as indicated. ^***^ *p* < 0.001 *vs* Sham. ^†^ *p* < 0.05, ^†††^ *p* < 0.001 *vs* IRI. ns, no statistical difference. (l) Representative micrographs of renal PAS and TUNEL staining in different groups as indicated. (m) Representative Western blot analyses of renal Ngal, Kim‐1, c‐Casp3 and c‐Casp9 protein levels in different groups as indicated. (n) Representative Western blot analyses of renal Sam50, Mitofilin, Opa1, Atad3a and Higd2a protein levels in different groups as indicated. (o) Schematic illustration of the protective mechanism of KP1 against kidney injury. KP1 enters renal tubular epithelial cells via endocytosis, targets mitochondrial protein ATAD3A, stabilizes the ATAD3A/HIGD2A protein complex, inhibits cytochrome c release and the subsequent caspase apoptosis cascade, thereby protecting against AKI. In addition, KP1 restores endogenous Klotho expression, which could play a role in renal protection. However, although KP1 has been reported to blocking TGF‐β/Smad3 signaling, it appears not involved in mediating KP1 protection in the setting of AKI.

We finally investigated whether KP1 can confer renal protection in established AKI, a scenario with clinical relevance. Label‐free live‐cell microscopy showed that CDDP caused an initial mitochondrial fission until 18 h regardless of KP1 presence (Figure 9a). However, KP1 could clearly restore the elongated mitochondrial morphology at 36 h after CDDP incubation in HKC‐8 cells, suggesting that KP1 has the potential to repair mitochondrial damage, leading to recovery (Movies S1 and S2). Time‐course studies revealed that KP1 could preserve ATAD3A protein at different time points after CDDP treatment (Figure S11).

Based on these findings, we carried out additional experiments in which KP1 was injected at 4, 12 and 24 h after CDDP (Figure 9b). As shown in Figure 9c,d, injection of KP1 even at 24 h after CDDP could effectively reduce Scr and BUN levels at 3 days. Consistently, histopathological assessment showed that KP1 mitigated CDDP‐induced morphological injury (Figure 9e). Delayed administration of KP1 also attenuated Kim‐1, Ngal, Parp‐1, p53, and c‐Casp9 expression, and largely restored mitochondrial proteins Opa1, Mitofilin, Sam50, Atad3a, and Higd2a in the kidneysafter CDDP treatment (Figure 9f–h and Figure S12a–j).

We also studied the effect of KP1 on established AKI induced by IRI (Figure 9i). As shown in Figure 9j,k, injection of KP1 at 4, 12 and 24 h after IRI also reduced Scr and BUN levels, with less effectiveness at 12 and 24 h. PAS and TUNEL staining revealed that KP1 was capable of preserving kidney morphological integrity and inhibiting apoptosis, even when it was given at 24 h post‐IRI. Similarly, delayed administration of KP1 at different time points was effective in reducing Kim‐1, Ngal, c‐Casp3 and c‐Casp9, and in preserving mitochondrial proteins Sam50, Mitofilin, Opa1, Atad3a and Higd2a in the kidney after IRI (Figure 9l–n and Figure S12k–s). These results suggest that KP1 not only prevents kidney failure after acute injury but also confers renal protection in established AKI. Collectively, these studies suggest that KP1 binds to mitochondrial protein ATAD3A, stabilizes the ATAD3A/HIGD2A protein complex, and prevents cytochrome c release from mitochondria into the cytosol, thereby protecting tubular cells against apoptosis after injury (Figure 9o).

## 3 Discussion

Kidney tubular epithelial cells are rich in mitochondria, reflecting their high energy demands. As such, the preservation of mitochondrial integrity and function is essential for maintaining renal tubular health and overall kidney function [14]. In this study, we demonstrate that KP1, a peptide derived from human Klotho and recapitulating its renoprotective potential, enters renal tubular cells via endocytosis and directly binds the mitochondrial protein ATAD3A and prevents its degradation, thereby preserving and sustaining its downstream effector HIGD2A. This process blocks cytochrome c release and caspase activation, ultimately inhibiting renal tubular cell apoptosis and providing protection against AKI (Figure 9o). Collectively, these studies elucidate the molecular mechanisms underlying mitochondrial dysfunction in AKI and provide a basis for developing KP1 as a therapeutic agent in combating AKI, a devastating condition presently with no effective therapies.

One of the novel findings in the present study is that KP1 mimics full‐length Klotho to protect against AKI (Figure 1). This protective effect of KP1 in the AKI setting appears to be independent of inhibiting canonical TGF‐β/Smad signaling as previously reported (Figure S3) [19], suggesting a novel mode of action. We show that KP1 enters tubular cells via endocytosis and directly targets and binds to mitochondrial protein ATAD3A, protecting against its degradation and stabilizing the ATAD3A/HIGD2A complex in the mitochondria, thereby preventing cytochrome c release, caspase activation, and tubular cell apoptosis. This conclusion is supported by several lines of evidence. First, FITC‐labelled KP1 can enter human proximal tubular HKC‐8 cells and kidney tubular cells in vivo, and blockade of endocytosis abolishes the protective potential of KP1 (Figure 3), suggesting that entering cells is necessary for KP1 to elicit its cytoprotective activities. Furthermore, KP1 binds to mitochondrial protein ATAD3A with high affinity as predicted by molecular docking simulation and confirmed by various sophisticated approaches such as co‐IP, MST, CETSA, and DARTS, thereby preserving it from degradation after injury (Figure 4). Moreover, overexpression of ATAD3A amelioras, whereas knockdown of ATAD3A accelerates, CDDP‐ and IRI‐triggered tubular injury and AKI, manifested by mitochondrial damage and apoptosis (Figures 5 and 6). Of interest, we have identified three amino acids of KP1 (G14, Y18 and Q19) that are important for binding to ATAD3A, and mutation of each of which disrupts KP1/ATAD3A interaction and abolishes the protective effect of KP1. As these three amino acids are completely identical in the Klotho protein of different species ranging from human to Drosophila [19], this suggests that the KP1/ATAD3A interaction may be evolutionarily conserved.

A particularly notable finding in this study is that KP1 confers renal protection in established AKI, a scenario with considerable clinical implications. Using label‐free live‐cell imaging, we showed that KP1 did not completely prevent mitochondrial fission and damage in tubular cells in the initial phase of injury after CDDP treatment, as evidenced by marked swelling, fragmentation, and disorganized arrangement. However, mitochondrial integrity is progressively recovered by KP1 within 36 h, ultimately restoring elongated rod‐shaped morphology with networked architecture (Figure 9 and Movies S1 and S2). Mitochondrial fission and fusion are dynamic processes, and stabilization of ATAD3A by KP1 would shift the balance toward the recovery of mitochondrial integrity. This may explain how KP1 restores the structure and function of the damaged mitochondria even after injury is established. Indeed, delayed administration of KP1 at the time point as late as 24 h after CDDP or IRI still alleviates AKI and restores mitochondrial proteins (Figure 9). The ability of KP1 to promote recovery even after injury is established distinguishes it from many previous candidates that only show benefit when given prophylactically. Furthermore, the restoration of mitochondrial proteins suggests that KP1 not only prevents further damage but actively engages repair mechanisms.

Klotho has been shown to alleviate mitochondrial dysfunction in kidney diseases through multiple mechanisms, including (i) activating AMPK‐PGC1α to alleviate mitochondrial damage in diabetic nephropathy [23], (ii) modulating the FoxO3‐BNIP3 axis to promote mitophagy in AKI [24], (iii) engaging Nrf2/HO‐1 signaling to mitigate mitochondrial dysfunction during renal aging [25, 26], and (iv) reducing mtDNA release to protect podocytes [27]. However, it remains unclear how Klotho, whether as a transmembrane protein or its soluble form present in the extracellular space, transduces its signal across the plasma membrane to modulate mitochondrial function. It is often presumed that Klotho may regulate mitochondrial integrity and its signaling indirectly via a receptor‐mediated transduction. The present study is the first to demonstrate that KP1, a Klotho‐derived peptide, directly enters cells to functionally engage with mitochondrial proteins. This unexpected finding challenges the existing paradigm of Klotho action and establishes a new framework for future investigation. Notably, we show that the His‐tagged sKlotho cannot enter tubular cells via endocytosis or bind to mitochondrial proteins in a manner similar to KP1 (Figure S7). This suggests that by entering tubular cells and interacting with ATAD3A, KP1 exerts previously unrecognized effects that go beyond those of its parental Klotho protein in protecting tubular cells against AKI.

The ATAD3A protein is a crucial regulator of mitochondrial biology and function [28]. It predominantly localizes across the inner and outer mitochondrial membranes in an oligomeric state and is essential for maintaining mitochondrial cristae organization and the integrity of mitochondrial nucleoids (mtDNA‐protein complexes) [28]. ATAD3A interacts with diverse mitochondrial proteins and effectors [28], orchestrating key regulatory processes including nucleoid trafficking [29], mitochondrial dynamics [30], mitophagy [31, 32], cancer metastasis [33], suppression of ER stress [34], mitochondrial structure and respiration maintenance [35], and cholesterol import [36]. As such, ATAD3A could represent a promising therapeutic target for ameliorating mitochondrial dysfunction. However, effective ATAD3A‐targeting agents have yet to be discovered. In this context, our identification of KP1 as a molecule that directly binds to and stabilizes ATAD3A is particularly significant, as this is the first study to address this critical knowledge gap. Accordingly, our findings not only unravel a novel mechanism of mitochondrial protection via KP1 and ATAD3A, but also underscore the potential therapeutic value of this pathway in treating various mitochondria‐associated pathologies that extend far beyond kidney disorders.

The present study further identifies HIGD2A, a member of the HIG1 hypoxia‐inducible domain (HIGD) protein family, as a novel ATAD3A interacting partner and downstream effector [37]. HIGD2A, a mitochondrial respiratory chain complex IV subunit localized to the inner membrane [38], is crucial for the proper assembly and stability of mitochondrial respiratory supercomplexes and regulates cytochrome c oxidase assembly and function [39]. As its name implies, HIGD2A helps cells adapt to low oxygen environments by maintaining mitochondrial function, thereby influencing energy metabolism, cell cycle progression [40], and hypoxic survival [41]. However, the expression and function of HIGD2A in the kidney have remained unexplored. Our findings demonstrate that HIGD2A, highly expressed in healthy kidney tubular epithelium, is downregulated in injured kidney after AKI, and KP1 can restore its expression. Furthermore, HIGD2A interacts with ATAD3A to form a complex (Figure 7), and overexpression or knockdown of HIGD2A attenuates or aggravates AKI‐induced tubular cell apoptosis by blocking cytochrome c release and caspase activation (Figure 8). Collectively, these studies establish that HIGD2A is a novel interacting partner and downstream effector of ATAD3A. In this context, it is conceivable to conclude that KP1 protects kidney tubular cells by directly and precisely targeting mitochondrial proteins ATAD3A and HIGD2A, leading to the preservation of mitochondrial function after AKI (Figure 9o).

The present study has several limitations and leaves many questions unanswered. For instance, IP/MS has identified numerous KP1‐interacting proteins, and it remains unknown whether other proteins besides ATAD3A also participate in mediating renal protection after AKI. In addition, KP1 could largely restore endogenous Klotho expression (Figure S3). Although it remains unclear whether it is a direct effect of KP1 on Klotho expression or a consequence of improved pathology in vivo, we cannot exclude the possibility that the restoration of endogenous Klotho expression plays, at least in part, a role in conferring renal protection. Of technical note, overexpression and knockdown of ATAD3A or HIGD2A in vivo were performed using a hydrodynamics‐based gene transfer technique. This approach also leads to transgene expression in the liver; therefore, kidney cell‐specific strategies would be needed in future studies. Finally, the relevance of these findings to human AKI warrants further investigation.

In summary, we demonstrate that KP1, a Klotho‐derived peptide, protects kidney function and alleviates tubular cell apoptosis after AKI. This protection is mediated through KP1 entry into tubular cells, directly binding to mitochondrial ATAD3A and stabilizing the ATAD3A‐HIGD2A complex, thereby leading to blockade of cytochrome c release and caspase activation. These findings establish KP1 as a first‐in‐class therapeutic agent for directly targeting mitochondria and provide a basis for developing novel remedies for treating patients with AKI or any other diseases associated with mitochondrial dysfunction.

## 4 Materials and Methods

### 4.1 Peptide Synthesis

KP1, FITC‐KP1, and KP1 alanine mutants were described previously [19] and synthesized by GenScript (Piscataway, NJ) with a purity of >95%. The amino acid sequence of KP1 is as follows: 57‐FQGTFPDGFLWAVGSAAYQTEGGWQQHGKG‐86. KP1 and its mutants were dissolved in 0.01 M acetic acid solution at a concentration of 10 µg/µL, whereas FITC‐KP1 was dissolved in DMSO at the same molar concentration.

### 4.2 Animal Models and KP1 Treatment

Animal studies were approved by the Animal Ethics Committee at the Nanfang Hospital, Southern Medical University. Male C57BL/6 mice aged 6–8 weeks were purchased from Beijing Vital River Laboratory Animal Technology Co. and housed in a standard environment in the Experimental Animal Center of Southern Medical University, with a 12‐h light/dark cycle and free access to water and chow. Animals were randomly assigned to the control and experimental groups, six mice per group. Surgery, drug injection, and tissue collection were performed in the same setting for each group in a continuous time period. For bilateral ischemia‐reperfusion injury (IRI), mice were anesthetized, and their kidneys were exposed, and the renal arteries were clamped with microaneurysm clamps on both sides of the renal arteries for 29 min. During ischemia, the body temperature of mice was maintained at 37.5°C. One day after reperfusion, mice were euthanized, and kidney tissues and blood were collected. The sham group underwent all procedures except for clamping of the renal arteries. Groups of mice were also subjected to cisplatin (CDDP) treatment. For the CDDP model, cisplatin (20 mg/kg) was administered intraperitoneally to mice. Kidney tissues and blood samples were collected 72 h later. The control group of mice received an equivalent volume of vehicle (saline) intraperitoneally. KP1 was administered daily via intravenous injection at a dose of 5 mg/kg/day, starting 2 days before IRI surgery or cisplatin injection.

### 4.3 Organ Imaging of KP1 Distribution

C57BL/6 mice were subjected to UIRI. One day after surgery, mice were subjected to depilation and intravenously injected with 200 µL of FITC‐KP1 (5  mg/kg) or FITC at the same molar concentration. Mice were euthanized at 1 and 24 h thereafter. Major organs including kidneys, heart, liver, lungs, and spleen were removed and placed on black paper. Organs were imaged using a Bruker FX PRO imaging system equipped with excitation at 470 nm and emission at 535 nm, and images were captured and digitally analyzed. All procedures were conducted in the dark.

### 4.4 Cell Culture and Treatment

Human kidney proximal tubular cells (HKC‐8) were provided by L. Racusen (Johns Hopkins University, Baltimore, MD). Human embryonic kidney cells (HEK‐293T) were purchased from the American Type Culture Collection (Manassas, VA). After 16–24 h of serum starvation, HKC‐8 cells were pretreated with KP1 or KP1 mutant (10 µg/mL), prochlorperazine (PCZ, 15 µM), FITC or FITC‐KP1 (3.1 µM) for 1 h prior to hypoxia‐reoxygenation (H/R) or CDDP treatment. In some experiments, pHrodo Red Dextran (0.1 mg/mL) (P10361; Invitrogen), PK Mito Orange (0.25 µM) (PKMO‐1; Genvivo Biotech), and Incucyte Caspase‐3/7 dye (5 µM) (4440; Sartorius AG, Göttingen, Germany) were added according to the manufacturer's instructions.

For hypoxia‐reoxygenation injury, cells were randomly divided into control (Ctrl) and hypoxia‐reoxygenation (H/R) groups. H/R groups were exposed to hypoxia (1% O_2_) for 24 h and then normoxia (20% O_2_) for 4–6 h, while control group cells were maintained under normoxic conditions throughout the culture period. For CDDP treatment, cells were exposed to cisplatin (7 µg/mL) for 24 h, whereas the control group received vehicle (DMSO) treatment.

### 4.5 Mouse Primary Proximal Tubular Epithelial Cells

Mouse primary proximal tubular epithelial cells were isolated and cultured as previously described [42]. Briefly, the cortical portion of mouse kidneys was minced and digested with 1 mg/mL collagenase IV (17104‐019; Gibco) using the standardized murine kidney dissociation protocol in the single‐cell suspension preparation instrument (DSC‐410; RWD, Shenzhen, China). After digestion, the mashed tissue was suspended in DMEM/F‐12 (C11330500CP; Gibco), and filtrated through a 70 µm cell strainer to remove undigested debris, discontinuous Percoll (17089109; Cytiva, Uppsala, Sweden) gradient centrifugation (35%/45%) was performed. Tubular fractions at the density interface were collected into DMEM/F‐12 medium supplemented with 10% fetal bovine serum, 50 U/mL penicillin‐streptomycin (15140‐122; Gibco). Cells were cultivated for 4–8 days until they reached 60%–80% confluency. Tubular epithelial cells were characterized by morphology, positive staining for E‐cadherin, and negative staining for vimentin. The purity of tubular epithelial cells was approximately 95%. The culture medium was changed on days 2 and 5, and every 3 days thereafter.

### 4.6 Western Blot Analysis

Proteins were extracted from kidney tissues and HKC‐8 cells and separated using sodium dodecyl sulfate‐polyacrylamide gel electrophoresis (SDS‐PAGE). The proteins were then transferred to a polyvinylidene fluoride (PVDF) membrane (IPVH00010; EMD Millipore Co, Billerica, MA), and blocked with 5% non‐fat milk before incubation with the appropriate primary antibodies. The protein bands were visualized using SuperEnhanced chemiluminescence detection reagents (P1010; Applygen Technologies Inc., Beijing, China). Blots were scanned, and band intensities were quantified using ImageJ software (National Institutes of Health, Bethesda, MD) and normalized to appropriate internal controls. The sources of antibodies used are listed in Table S1.

### 4.7 RT‐qPCR

Total RNA was extracted from kidney tissues and HKC‐8 cells using TRIzol Reagent Kit (Invitrogen, Carlsbad, CA). Total mRNA was reverse‐transcribed to cDNA using the GoScript Reverse Transcription System Kit (Promega, Madison, WI, USA). The specific primers for mouse*Tnfa*, human *TNFα* mouse *Atad3a*, and human *ATAD3A* are given in Table S2. The mRNA levels were quantitatively analyzed using SYBR Green PCR Master Mix (Promega, Madison, WI, USA), while β‐actin was used as an internal control.

### 4.8 Cell Transfection

ATAD3A overexpression plasmid and HIGD2A overexpression plasmid were obtained from GenePharma (Suzhou, China). Human ATAD3A siRNA (sc‐88047) and HIGD2A siRNA (sc‐91606) were purchased from Santa Cruz. HKC‐8 cells were cultured in 6‐well plates for 16–24 h before transfection. Prior to transfection, the culture medium was replaced with serum‐reduced medium (31985‐070; Opti‐MEM I, Gibco, Carlsbad, CA). The transfection was carried out with Lipofectamine 2000 (11668019; Invitrogen, Carlsbad, CA) according to the manufacturer's instructions. Plasmid (0.5 µg/µL) or siRNA (20 nM) was mixed with Lipofectamine 2000 and added to HKC‐8 cells and incubated for 6–8 h, and the transfection mixture was replaced with 10% FBS‐containing medium and cultured for another 24 h. The sequences of human ATAD3A overexpression plasmid, human HIGD2A overexpression plasmid, and pcDNA3.1 plasmid (V79020; Invitrogen) are listed in Table S3.

### 4.9 Hydrodynamics‐Based Gene Transfer in Vivo

The expression plasmids used in this study were custom‐synthesized by GenePharma (Suzhou, China) and prepared using QIAfilter Plasmid Midi and Maxi Kits (12263; QIAGEN, Hilden, Germany) according to the manufacturer's instructions. Plasmid injections were performed using a hydrodynamics‐based gene transfer technique, as described previously [43]. Briefly, plasmids were injected intravenously at a dose of 2.5 mg/kg (diluted with a total volume of 2 mL of saline) 2 days before IRI surgery or cisplatin injection. The sequences of mouse ATAD3A shRNA, mouse ATAD3A overexpression plasmid, and pcDNA3.1 plasmid (V79020; Invitrogen) are presented in Table S3.

### 4.10 Co‐Immunoprecipitation

The procedure was performed according to the manufacturer's instructions for Protein A/G PLUS‐Agarose (sc‐2003; Santa Cruz Biotechnology, Dallas, TX), as described previously [44]. Captured proteins were subjected to either Western blot analysis or high‐throughput mass spectrometry.

### 4.11 Histology and Immunohistochemical Staining

Paraffin‐embedded mouse kidney sections were prepared by a routine procedure. Periodic acid‐Schiff (PAS) staining was performed to evaluate the extent of renal tubular injury, while TUNEL staining (G3250; Promega, Madison, WI) was employed to quantify apoptosis. After antigen unmasking, sections were incubated with primary antibodies overnight at 4°C, followed by incubation with biotinylated secondary antibodies and developed with aminoethylcarbazole (AEC) substrate (SK‐4200; Vector Laboratories, Newark, CA). The tissue sections were counterstained with hematoxylin. Image quantification was performed using Image Pro‐Plus version 6.0 software (Media Cybernetics, Bethesda, MD). The sources of antibodies used are listed in Table S1.

### 4.12 Immunofluorescent Staining

Paraffin‐embedded mouse kidney sections and coverslip‐fixed cells were prepared by a routine procedure. After antigen unmasking, sections and coverslips were incubated with primary antibodies and fluorophore‑conjugated secondary antibodies. Samples were counterstained with DAPI for nuclear labeling and imaged using a confocal microscope (Leica, Wetzlar, Germany). The sources of antibodies used are listed in Table S1.

### 4.13 Flow Cytometry

Cell suspensions were transferred to flow cytometry tubes. Fluorescence channels were selected, and forward scatter (FSC) and side scatter (SSC) voltages were adjusted with gating strategies established based on control samples. Samples were analyzed using a BD Biosciences flow cytometer. Apoptosis rates were quantified using PE Annexin V Apoptosis Detection Kit I (559763; BD Biosciences, Franklin Lakes, NJ) according to the manufacturer's instructions.

### 4.14 Microscale Thermophoresis (MST)

The GFP‐ATAD3A fusion plasmid was transfected into HEK‐293T cells, followed by lysis in RIPA buffer. KP1 was serially diluted twofold from 200 µM (16 concentrations) and mixed with equal volumes of cell lysate. Samples were loaded into standard capillaries in concentration‐gradient order and analyzed on a Monolith NT.115 instrument (NanoTemper) for dissociation constant (*Kd*) determination. All procedures were conducted in 0.01 M acetic acid buffer at 25°C. Control groups underwent identical procedures using a GFP‐expressing plasmid and matched KP1 concentrations.

### 4.15 Molecular Docking Simulation

A peptide model was constructed using Avogadro software (version 1.2.0) with AMBER force field optimization and docked via the ZDock server (version 3.0.2). The top‐ranked conformation (ZDock score > 120) was analyzed using PDBePISA to calculate binding free energy. Complexes exhibiting ΔG < ‐5 kJ/mol were subjected to 100‐ns molecular dynamics simulations to verify stability.

### 4.16 Drug affinity Responsive Target Stability (DARTS)

ATAD3A‐overexpressing HEK‐293T cells were lysed in M‐PER buffer (78501; Thermo Fisher Scientific) supplemented with phenylmethylsulfonyl fluoride (PMSF) (36978; Thermo Scientific). For binding assays, KP1 (0, 25, or 100 µM) was incubated with equal volumes of lysate (5 µg/µL) for 30 min at room temperature. Resultant complexes were digested with Pronase (10165921001; Roche Diagnostics GmbH, Mannheim, Germany) at enzyme‐to‐mixture ratios of 1:100, 1:500, and 1:1000 for 10 min at room temperature. Proteolytic fragments were analyzed by Western blotting using anti‐ATAD3A antibodies or subjected to LC‐MS/MS on a mass spectrometer.

### 4.17 Cellular Thermal Shift Assay (CETSA)

CETSA was performed to evaluate the thermal stabilization of ATAD3A in the absence or presence of KP1. Briefly, ATAD3A‐overexpressing cells treated with KP1 or vehicle for 1 h were harvested and resuspended in PBS supplemented with a protease inhibitor cocktail. Cell suspensions were aliquoted equally and incubated at each temperature across a gradient of 39–57°C for 5 min, followed by cooling at room temperature for 3 min. Samples were lysed via three repeated freeze‐thaw cycles (each cycle consisted of 3 min of flash‐freezing in liquid nitrogen, followed by 3 min of thawing in a 25°C metal bath), then centrifuged at 12 500 rpm for 20 min at 4°C to remove insoluble protein aggregates. Supernatants were collected and analyzed by Western blotting. Relative ATAD3A levels were quantified to generate melting curves, and melting temperature (T_m_) values were compared to assess target engagement.

### 4.18 Mitochondrial Isolation

Mitochondria were isolated from fresh kidney tissues and cells using a mitochondrial isolation kit (ABS9343 for tissues; ABS9344 for cells; Absin, Shanghai, China) according to the manufacturer's instructions. Renal tissue fragments or cells were thoroughly homogenized in ice‐cold isolation buffer using a Dounce tissue grinder, and mitochondria were subsequently isolated by density gradient centrifugation.

### 4.19 Mitochondrial Proteomic Analysis via DIA Mass Spectrometry

The proteomic analysis in this study was performed by Cosmos Wisdom Corporation (Hangzhou, China). Proteins extracted from isolated mitochondria were digested into peptides with trypsin. Chromatographic separation was carried out using the Vanquish Neo nanoflow system (Thermo Fisher Scientific), and samples post high‐efficiency liquid chromatography separation were subjected to data‐independent acquisition (DIA) mass spectrometry using an Astral high‐resolution mass spectrometer (Thermo Fisher Scientific, Waltham, MA). DIA data were processed using DIA‐NN software. Bioinformatics analyses included protein annotation (GO annotation, protein domain annotation, KEGG pathway annotation, and subcellular localization), functional enrichment, and protein‐protein interaction.

### 4.20 Targeted Peptide Quantification via PRM Mass Spectrometry

Chromatographic separation was performed using the Vanquish Neo nanoflow system, and data acquisition was performed in parallel reaction monitoring (PRM) mode using an Orbitrap Astral high‐resolution mass spectrometer (Thermo Scientific). The detection was in positive mode, with a scan range of 380–980 m/z for MS1 precursor ions, and a resolution of 240,000 at 200 m/z. Normalized AGC Target was set at 500%, with a maximum injection time of 5 ms. MS2 employing the PRM data acquisition mode was performed with a scheduled window (2 min) method according to the specified inclusion list (an Isolation Window of 1 m/z, HCD Collision Energy set at 25 eV, Normalized AGC Target at 1000%, and a Maximum IT of 20 ms). The Skyline software (version 22.2; MacCoss Lab, University of Washington, Seattle, WA) was used for PRM method development and peak detection. The extracted peak intensities were exported for downstream statistical analyses.

### 4.21 Transmission Electron Microscopy and Immunogold Electron Microscopy

Renal tissue and cellular samples for this study were processed and imaged by electron microscopy at Guangzhou Huayin Medical Laboratory Center Co (Guangzhou, China). Immunogold labeling for FITC‐KP1 was performed using a specific anti‐FITC antibody. The sources of antibodies used are listed in Table S1.

### 4.22 Stimulated Emission Depletion (STED) Imaging

Cells were incubated with PK Mito Orange (PKMO‐1; Genvivo Biotech, Nanjing, China) dye according to the manufacturer's protocol, followed by image acquisition using a STEDYCON microscope (Abberior Instruments, Göttingen, Germany) equipped with 100x objective lens. Mitochondrial quantification was performed in ImageJ with uniform parameters across all samples. Briefly, images were calibrated for spatial scale, processed with background subtraction and mild Gaussian blur, then binarized by Otsu auto‐thresholding and segmented via the Watershed algorithm. Morphology was quantified using the Analyze Particles function: size range 0.1–5 µm^2^, circularity 0–1, and edge objects excluded. Five random fields were analyzed per group, and the mean value of each field was treated as one independent data point.

### 4.23 Label‐Free Live Cell Microscopy Imaging

Cells were captured by a label‐free live‐cell microscopy system (SC3000Pro; IDT channels; Zircon Optics Inc., China) equipped with a 40× objective lens.

### 4.24 Longitudinal High‐Throughput Live‐Cell Analysis

Apoptosis dynamics were monitored on the Incucyte SX5 Live‐Cell Analysis System (Sartorius AG, Göttingen, Germany) using Caspase‐3/7 Green Reagent, with phase‐contrast and fluorescence images captured at 1.5‐h intervals over 36 h under physiological conditions (37°C, 5% CO_2_). Cell viability and apoptosis indices were automatically quantified using Incucyte Software (version 2023.1B; Sartorius AG).

### 4.25 Detection of Serum Creatinine and Blood Urea Nitrogen

Serum creatinine (Scr) and blood urea nitrogen (BUN) levels were measured using an automatic chemistry analyzer (AU480; Beckman Coulter, Brea, CA). The levels of Scr and BUN were expressed in mg/dL.

### 4.26 Statistical Analysis

All data are presented as mean ± SEM from at least three independent experiments in cell culture and six mice per group. Statistical analyses were performed using GraphPad Prism 9.0.0 (GraphPad Software, San Diego, CA, USA). Comparisons between two groups were assessed using unpaired two‑tailed *t*‑tests, and comparisons among multiple groups were analyzed by one‑way ANOVA followed by LSD or Dunnett's post hoc tests. A *p* value < 0.05 was considered statistically significant.

## Author Contributions

Conceptualization: Liu Y; methodology: Zhang X, Zhou H; investigation: Zhang X, Zhou H; visualization: Zhang X; funding acquisition: Zhang X, Liu Y; project administration: Liu Y; formal analysis: Zhang X; data curation: Zhang X; supervision: Liu Y; resources: Wu T, Zhang Z, Lin S, Hong X; validation: Wu T, Zhang Z, Lin S, Hong X, Liu Y; writing – original draft: Zhang X; writing – review & editing: Wu T, Zhang Z, Lin S, Hong X, Liu Y.

## Conflicts of Interest

The authors declare no conflicts of interest.

## Supporting information




**Supporting File 1**: advs77214‐sup‐0001‐SuppMat.pdf.

**Supporting File 2**: advs77214‐sup‐0002‐WB_Raw_Data.pdf.

**Supporting File 3**: advs77214‐sup‐0003‐MovieS1.mp4.

**Supporting File 4**: advs77214‐sup‐0004‐MovieS2.mp4.

## Data Availability

Proteomics data have been deposited at iProX (https://www.iprox.cn) with accession numbers PXD072323 and PXD082248, and are publicly available as of the date of publication. Original western blot images have been submitted as a part of supplementary. Any additional information is available from the corresponding author upon request.
