# Supplementary material for: Klotho‐Derived Peptide 1 Protects against Acute Kidney Injury by Directly Targeting Mitochondrial ATAD3A
Source: Adv Sci (Weinh). 2026 Aug 25;13(63):e77214. doi: 10.1002/advs.77214 (PMC13505471; doi:10.1002/advs.77214)
Supplement: Supplementary file 2 [file ADVS-13-e77214-s002.pdf]

Figure 1.

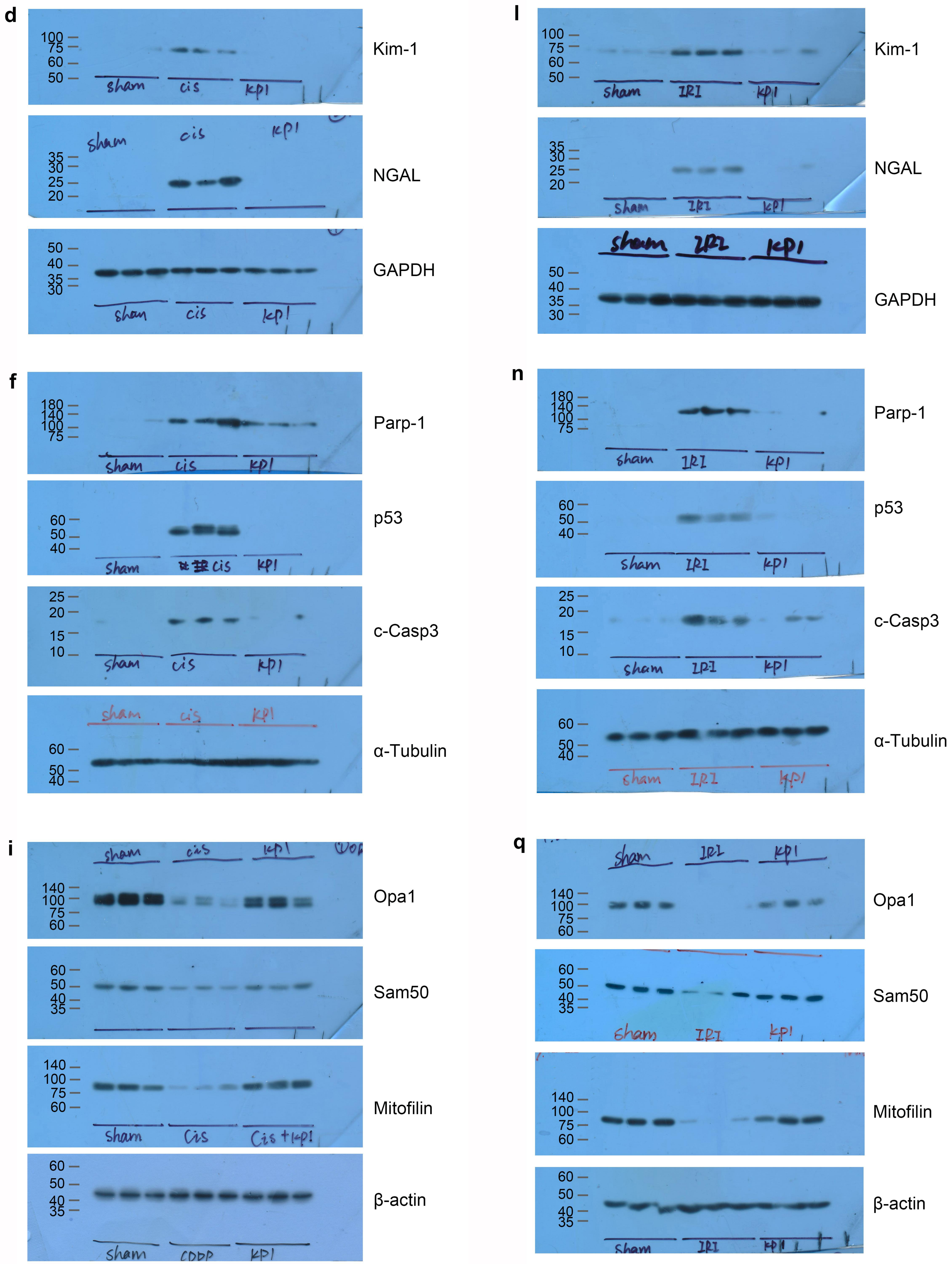
d
Kim-1
NGAL
GAPDH
l
Kim-1
NGAL
GAPDH
f
Parp-1
p53
c-Casp3
α-Tubulin
n
Parp-1
p53
c-Casp3
α-Tubulin
i
Opa1
Sam50
Mitofilin
β-actin
q
Opa1
Sam50
Mitofilin
β-actin

Figure 2.

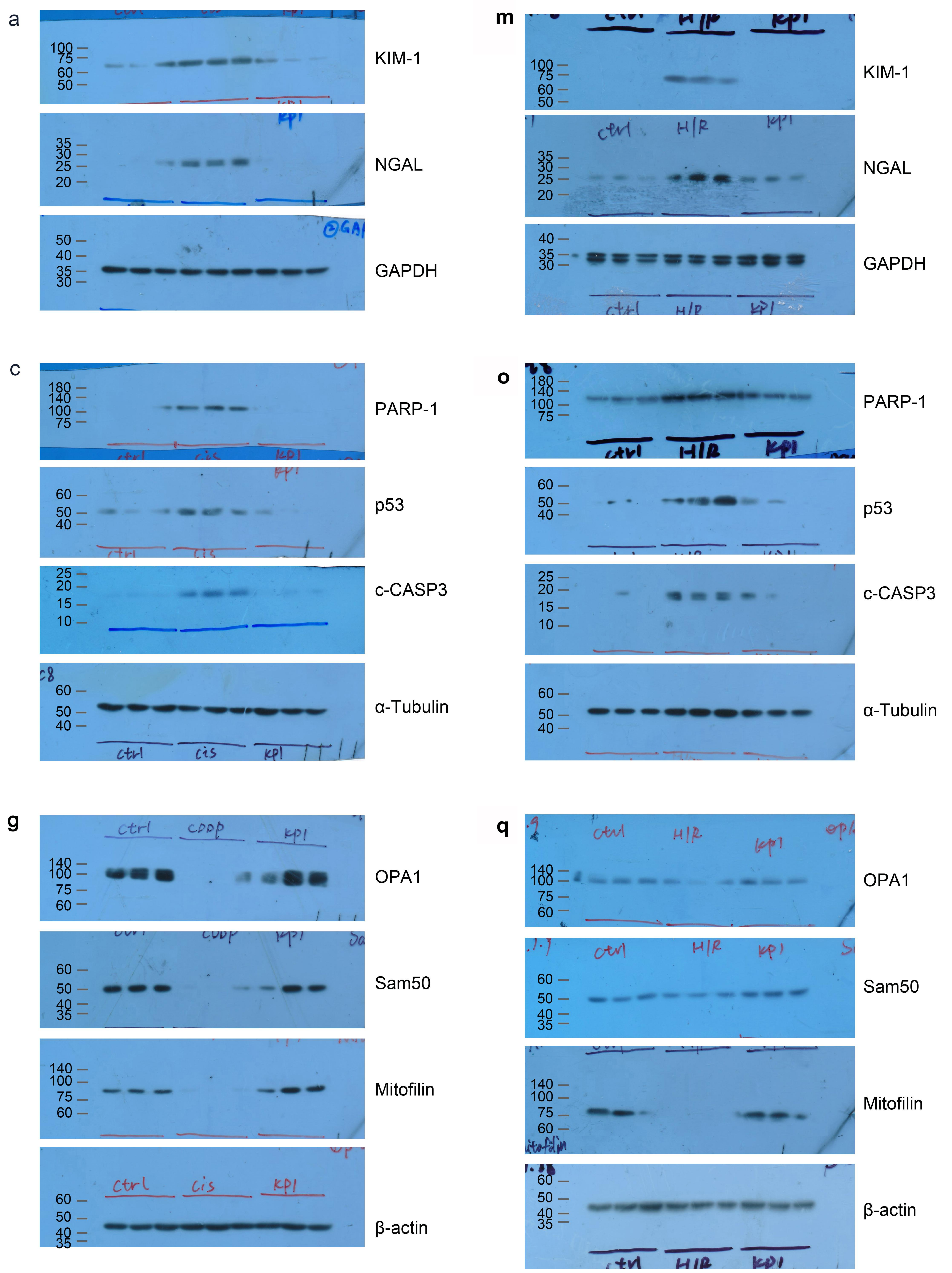

a
KIM-1
NGAL
GAPDH
c
PARP-1
p53
c-CASP3
α-Tubulin
g
OPA1
Sam50
Mitofilin
β-actin
m
KIM-1
NGAL
GAPDH
o
PARP-1
p53
c-CASP3
α-Tubulin
q
OPA1
Sam50
Mitofilin
β-actin

Figure 3.

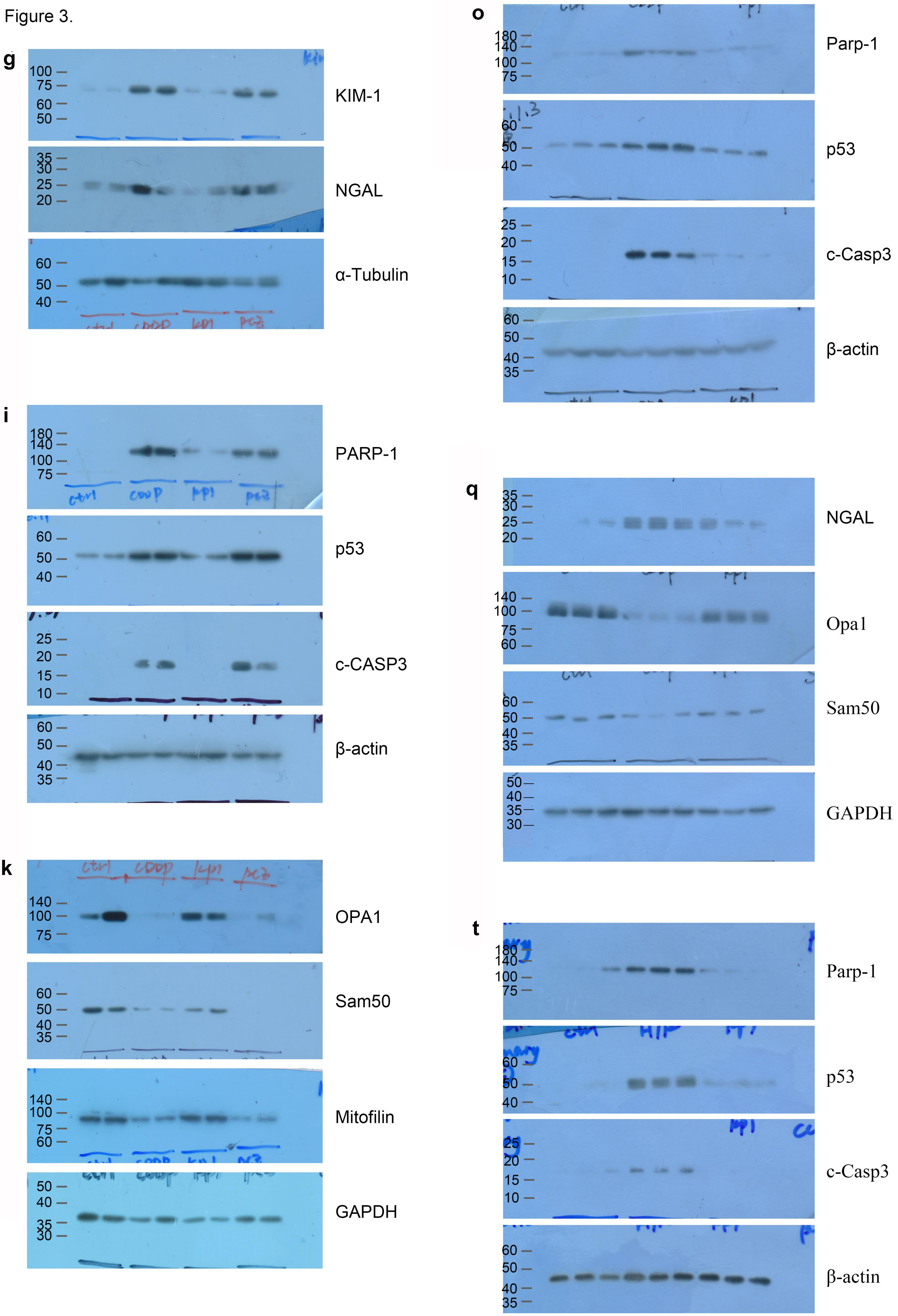
g
KIM-1
NGAL
α-Tubulin
i
PARP-1
p53
c-CASP3
β-actin
k
OPA1
Sam50
Mitofilin
GAPDH
o
Parp-1
p53
c-Casp3
β-actin
q
NGAL
Opa1
Sam50
GAPDH
t
Parp-1
p53
c-Casp3
β-actin

Figure 3.

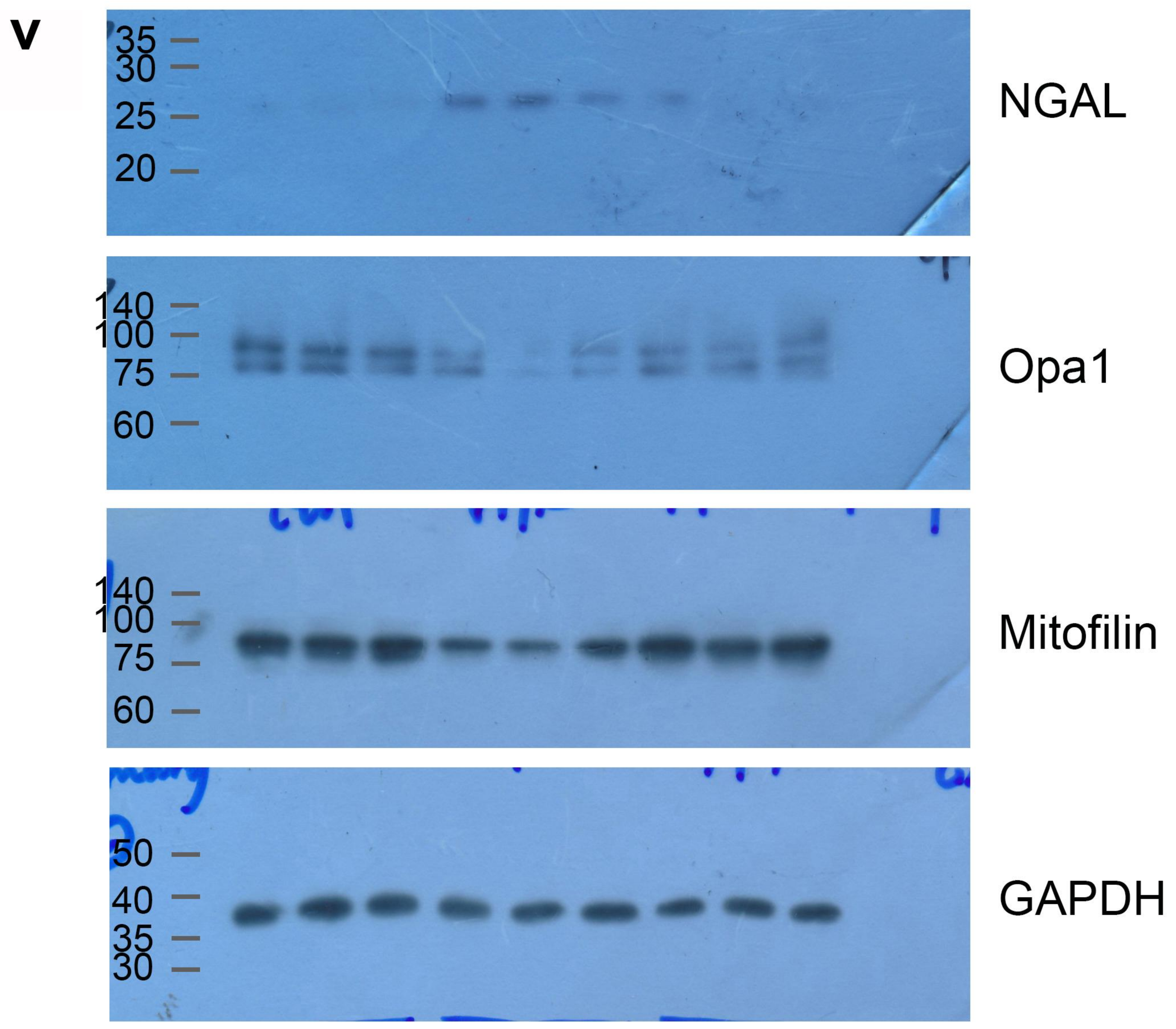

Figure 4.

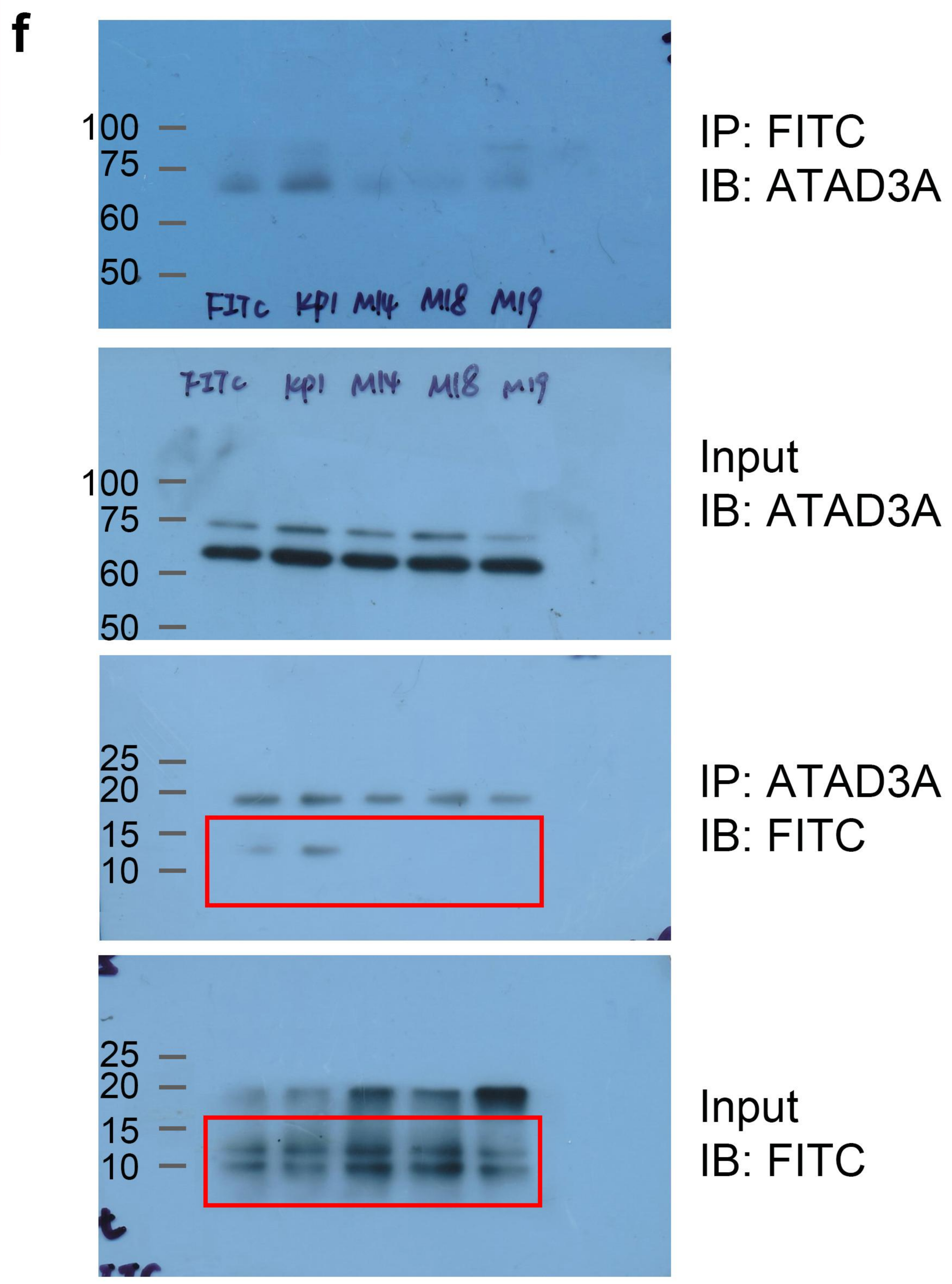

Figure 4.

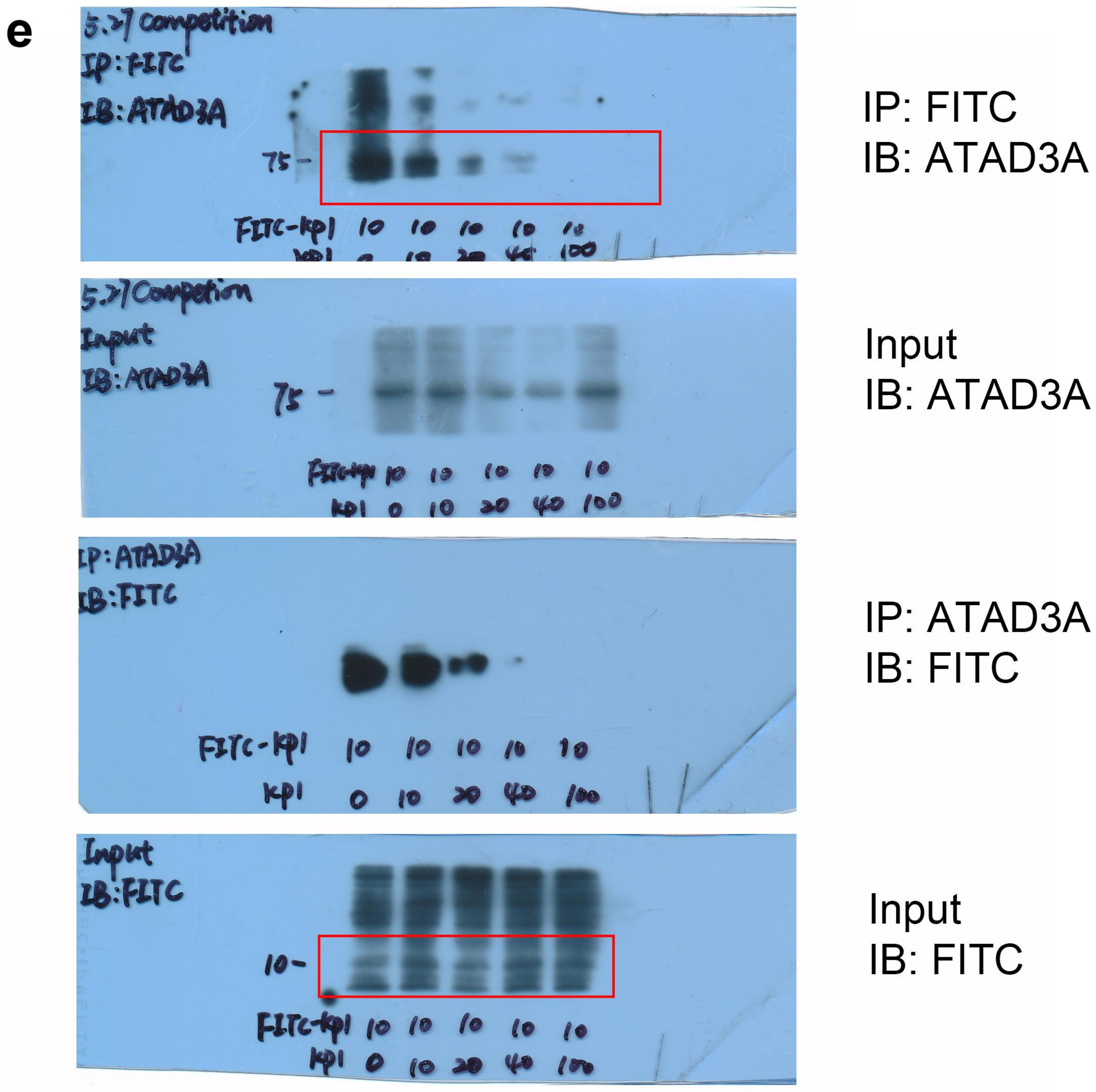

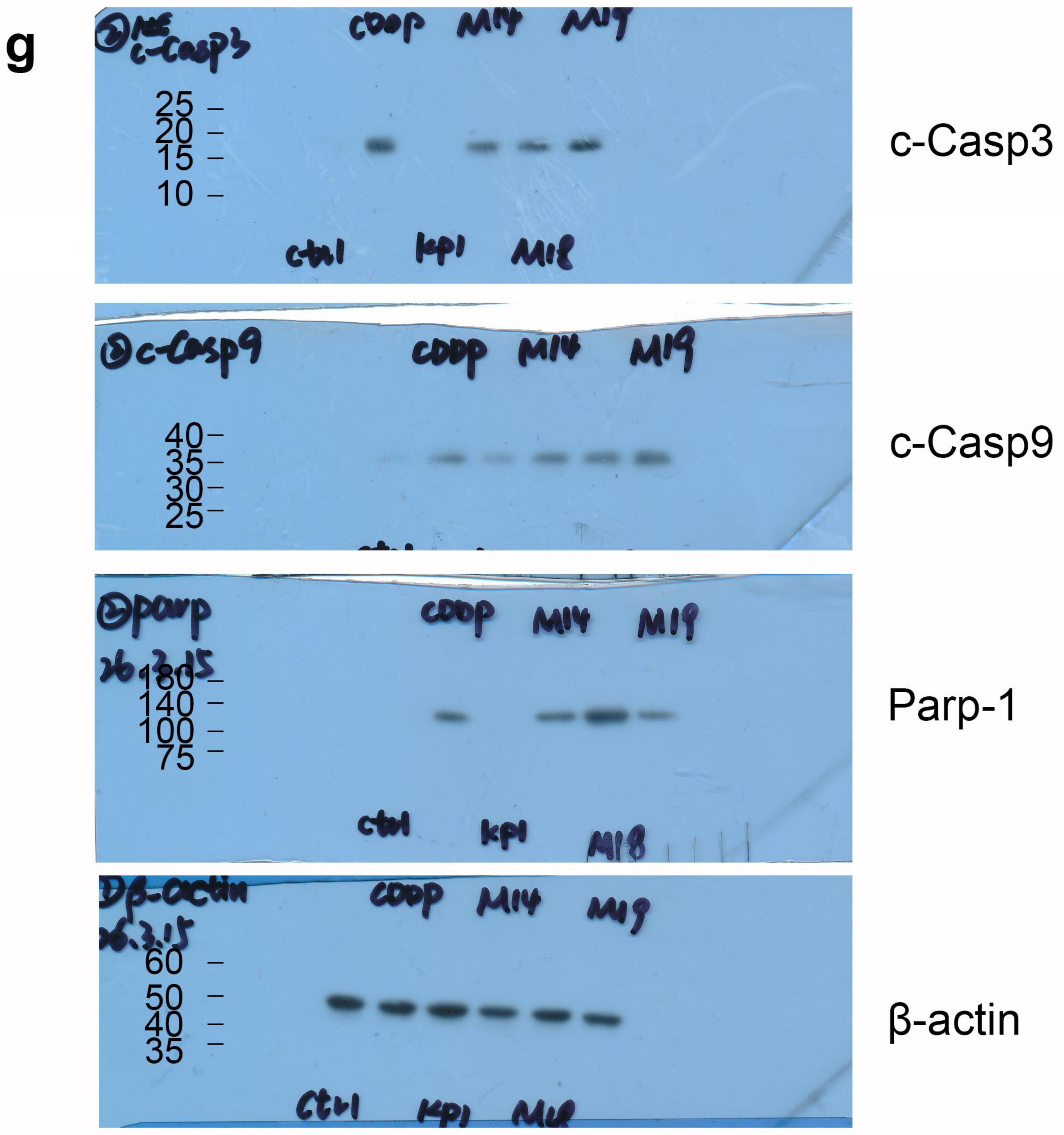

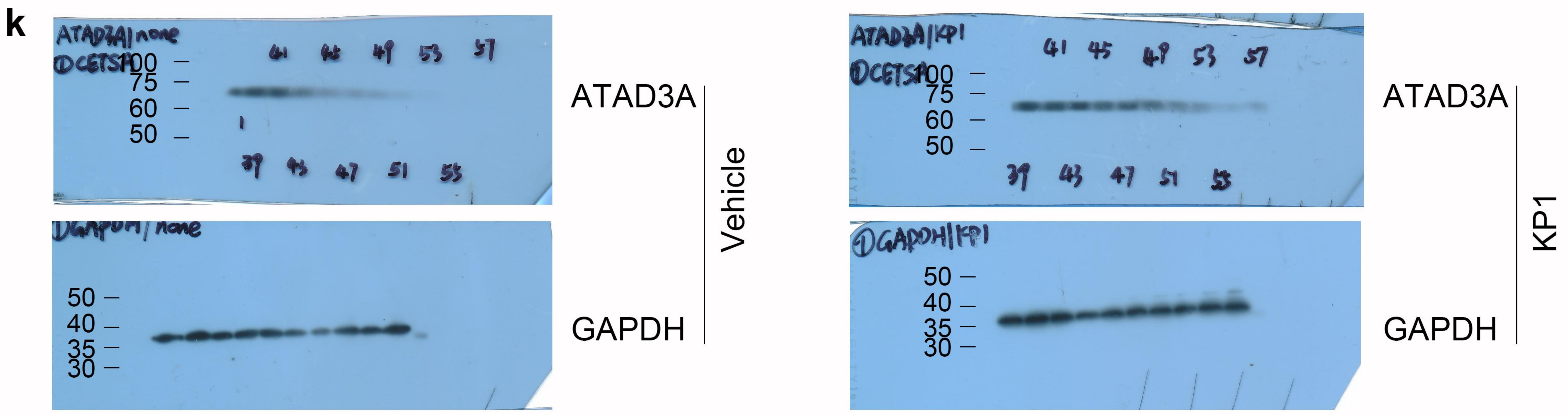

Figure 4

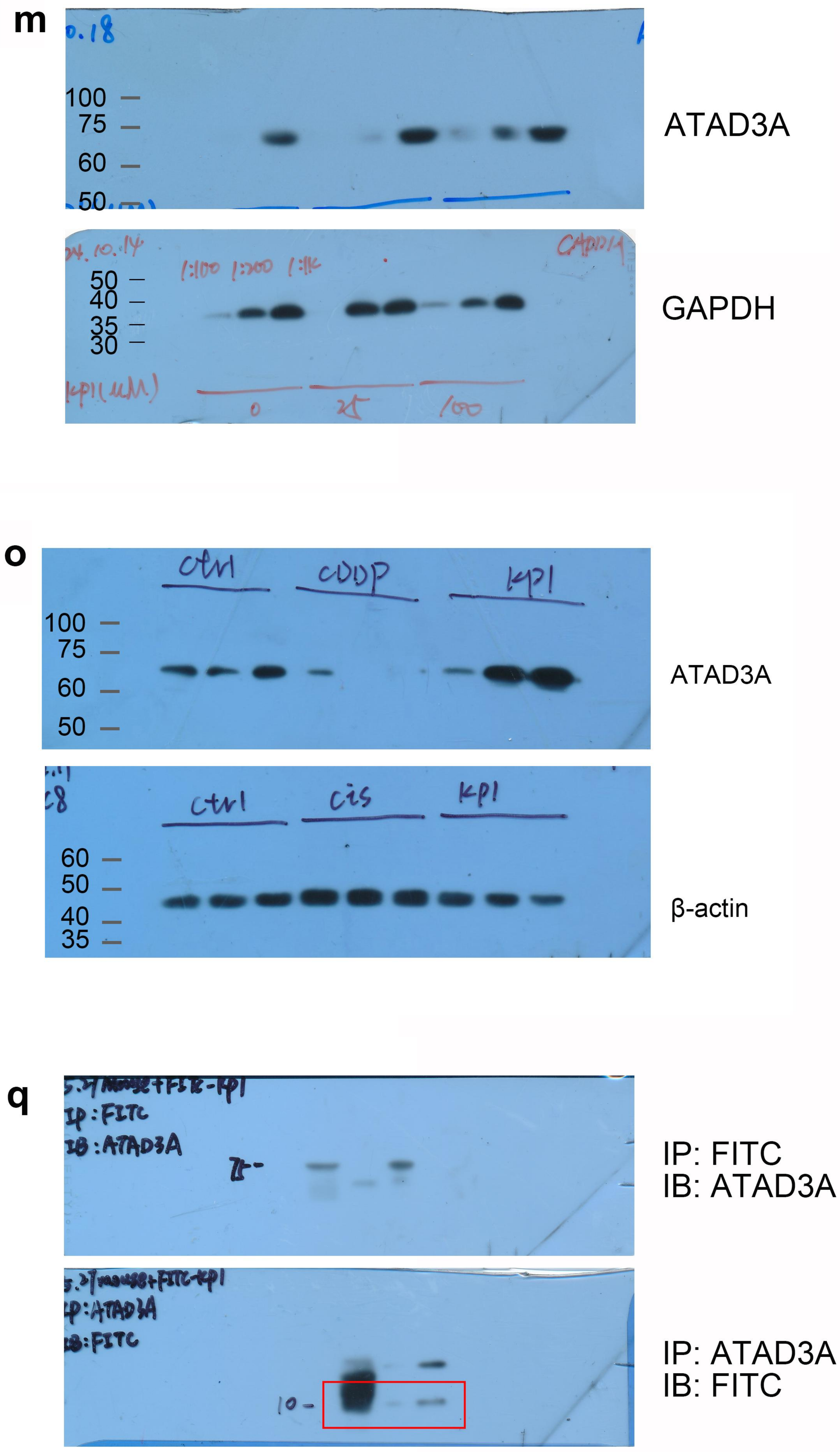

Figure 5.

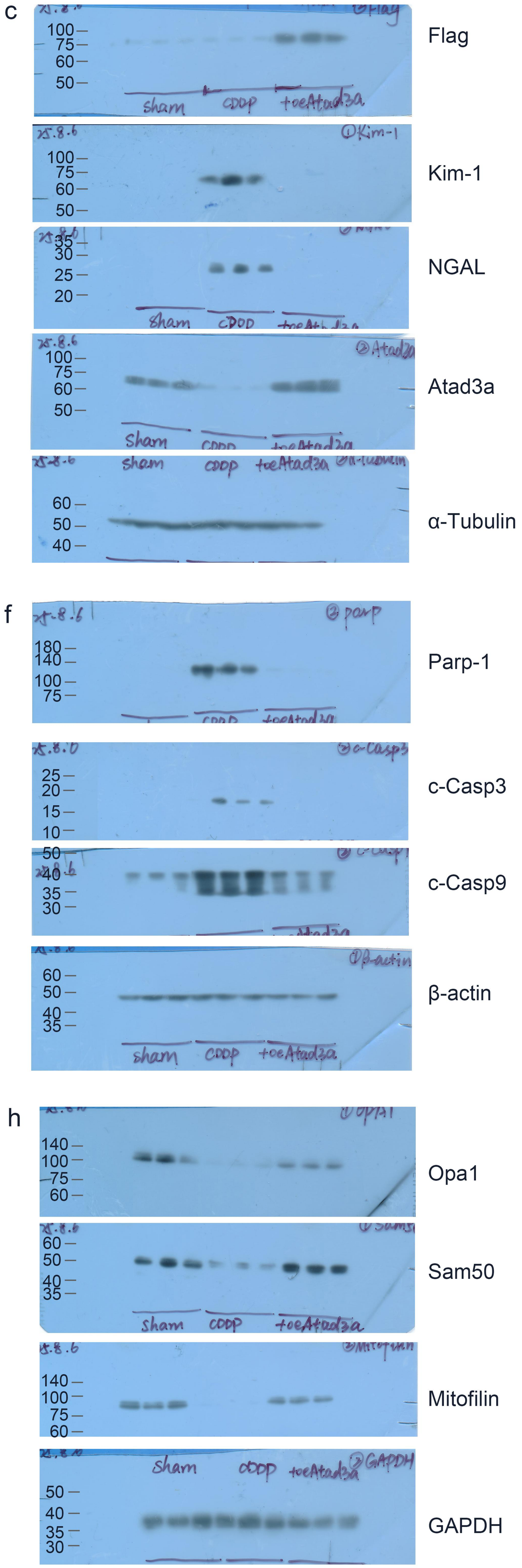

Figure 5.

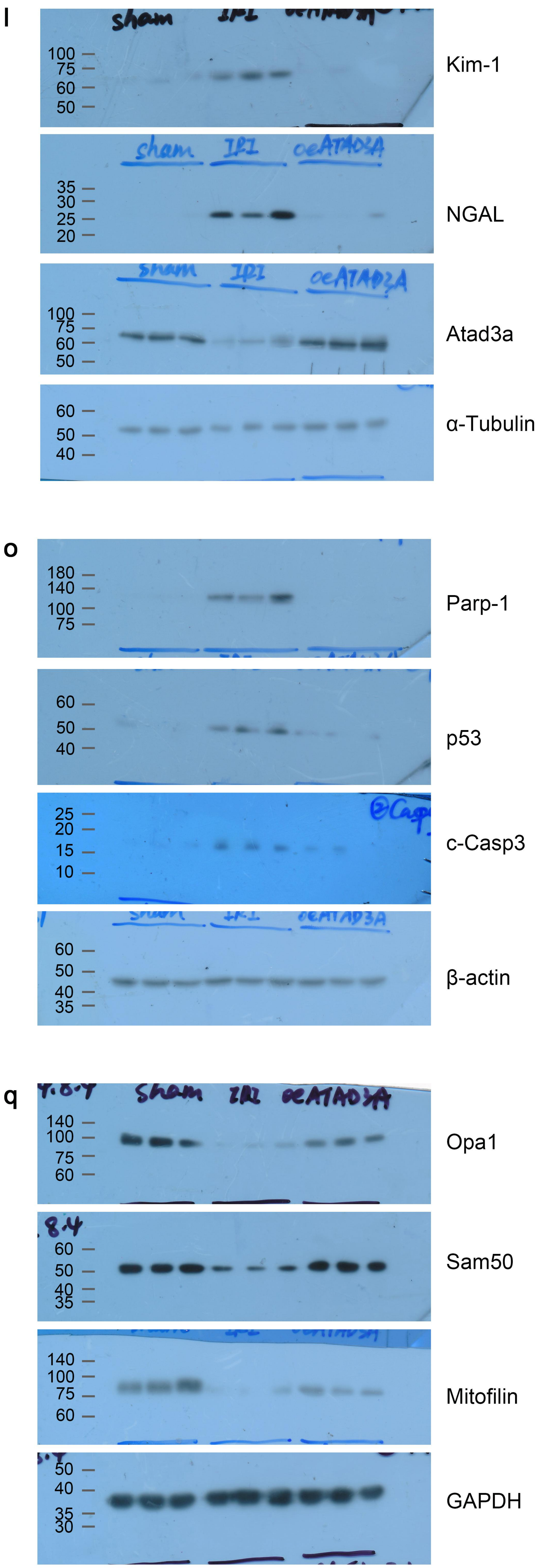

Figure 6.

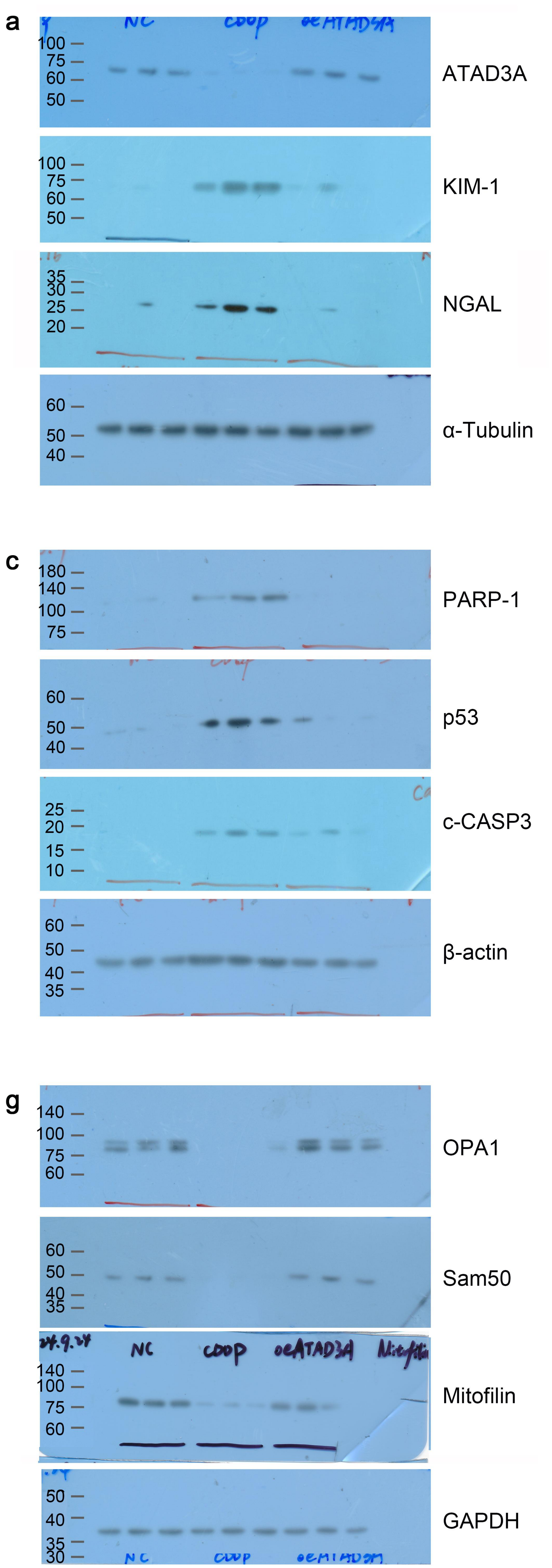

Figure 6.

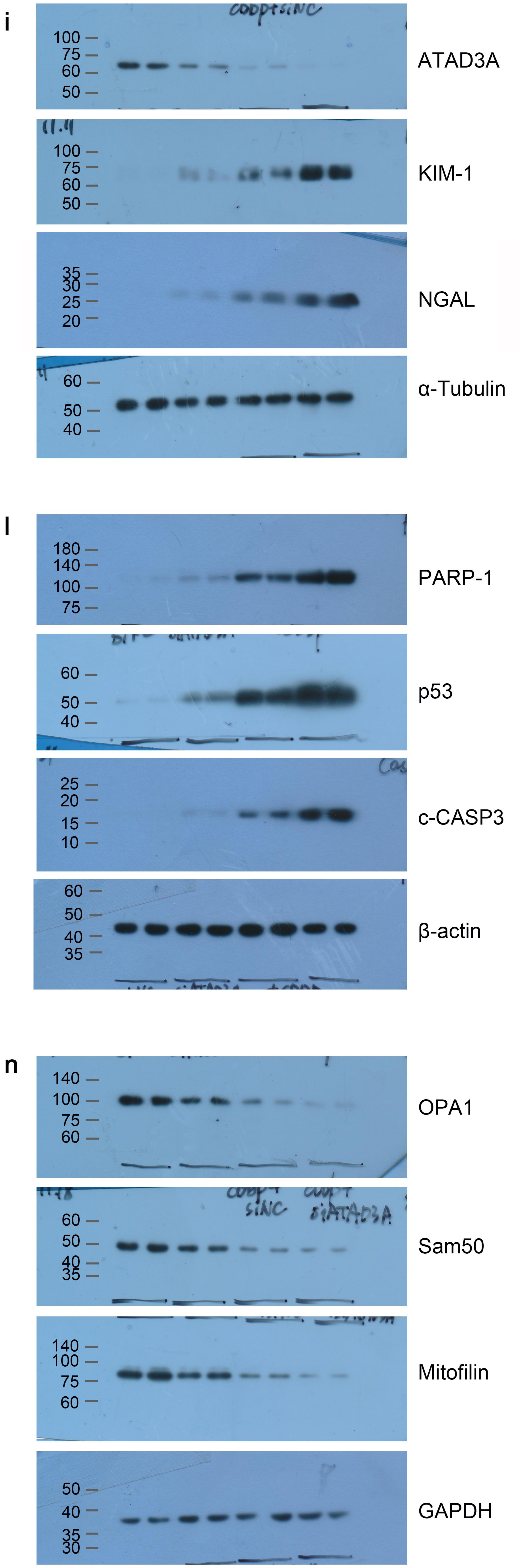

Figure 7.

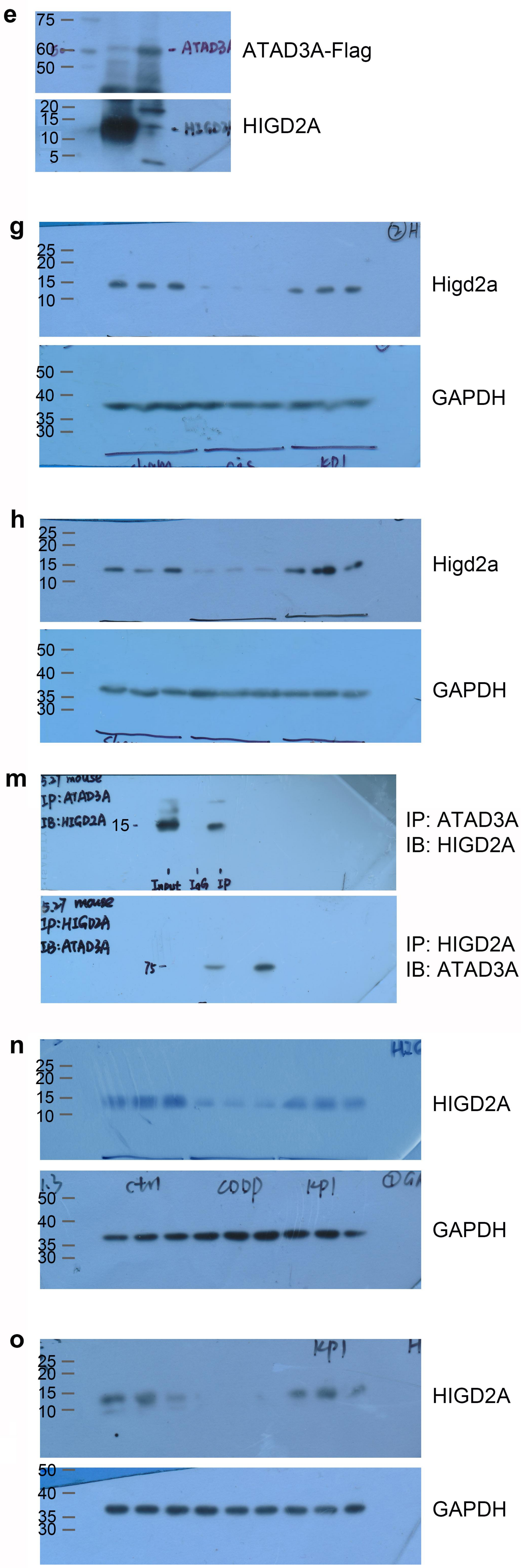

Figure 7.

r

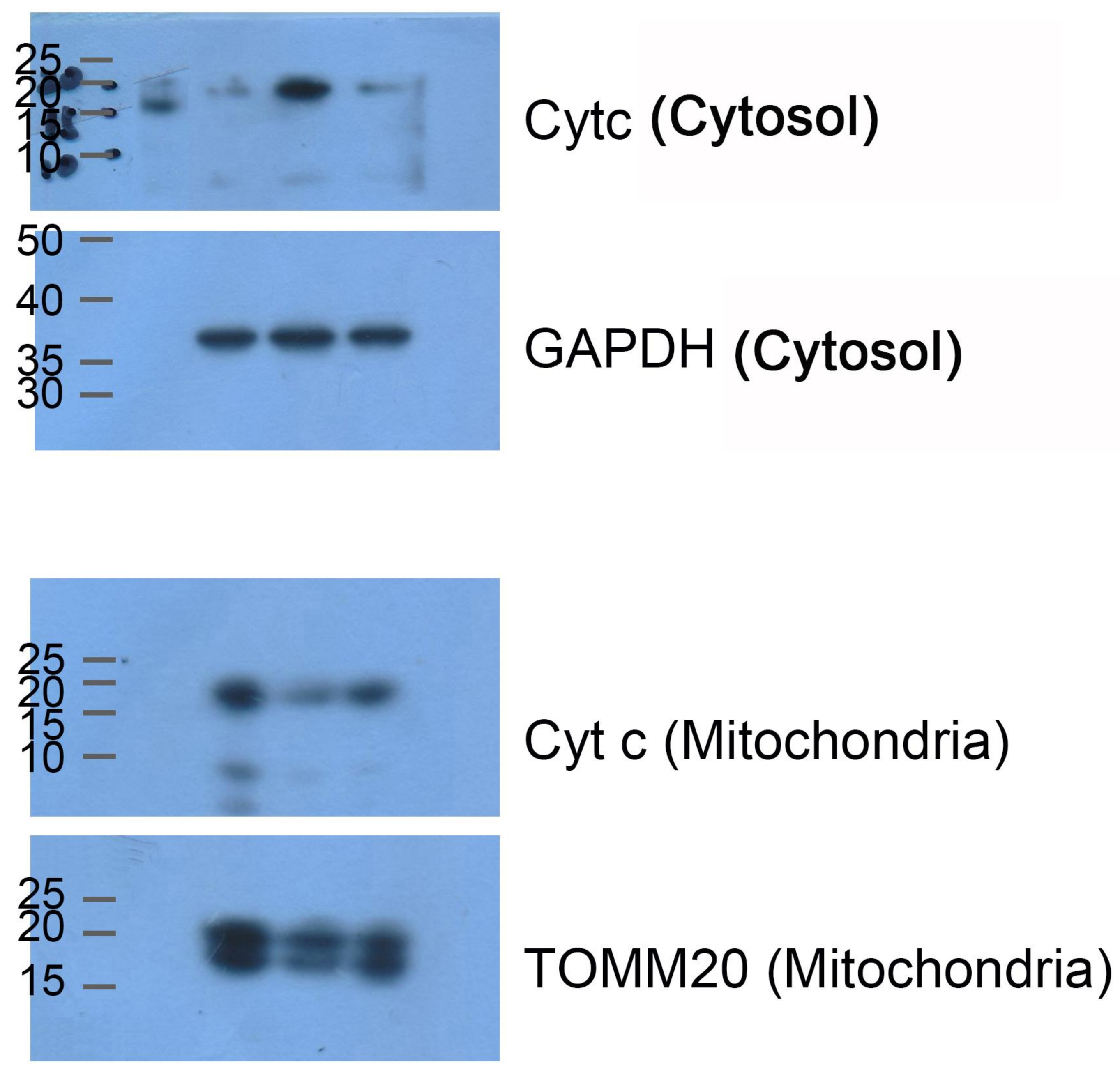

s

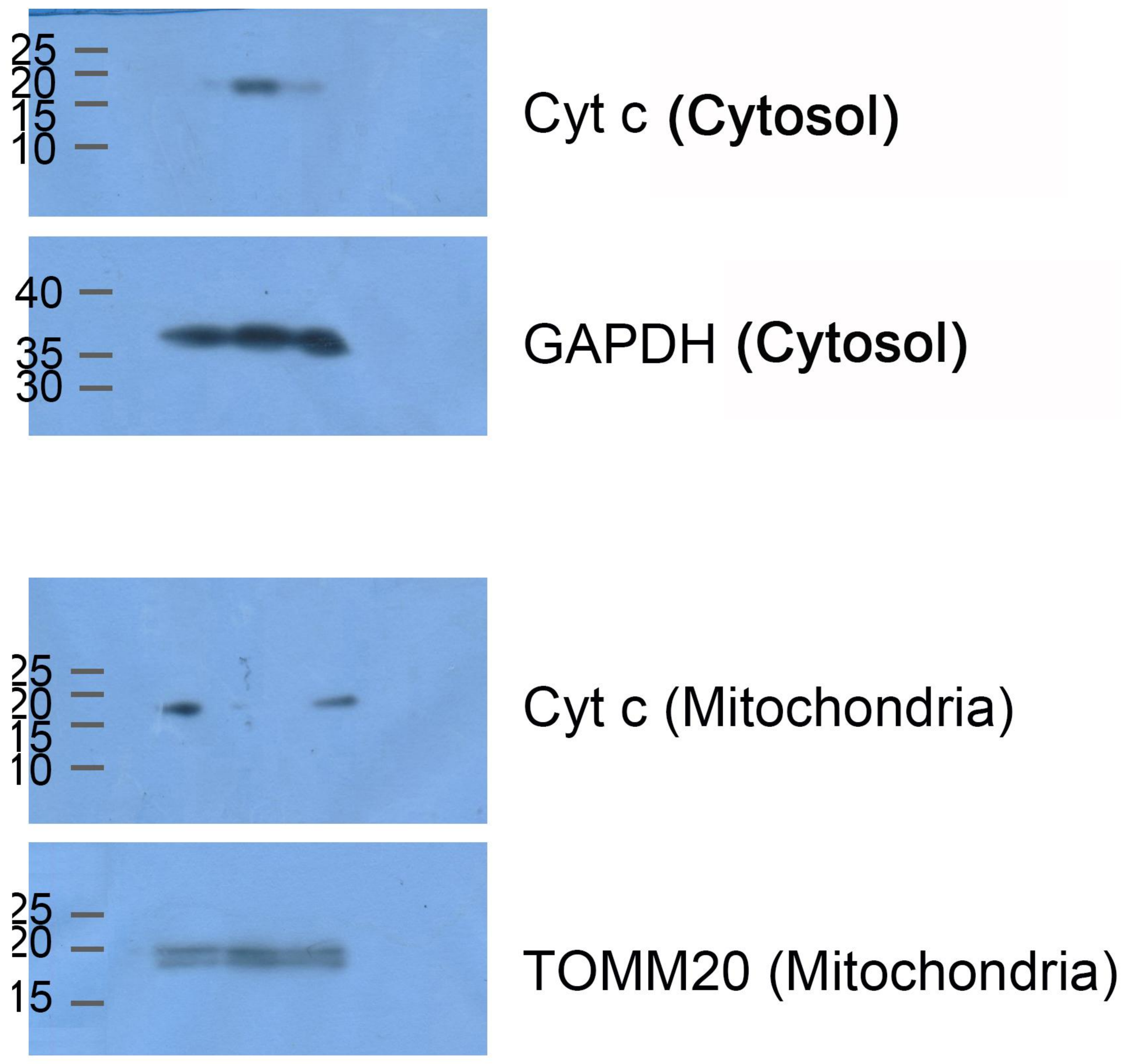

Figure 8.

a

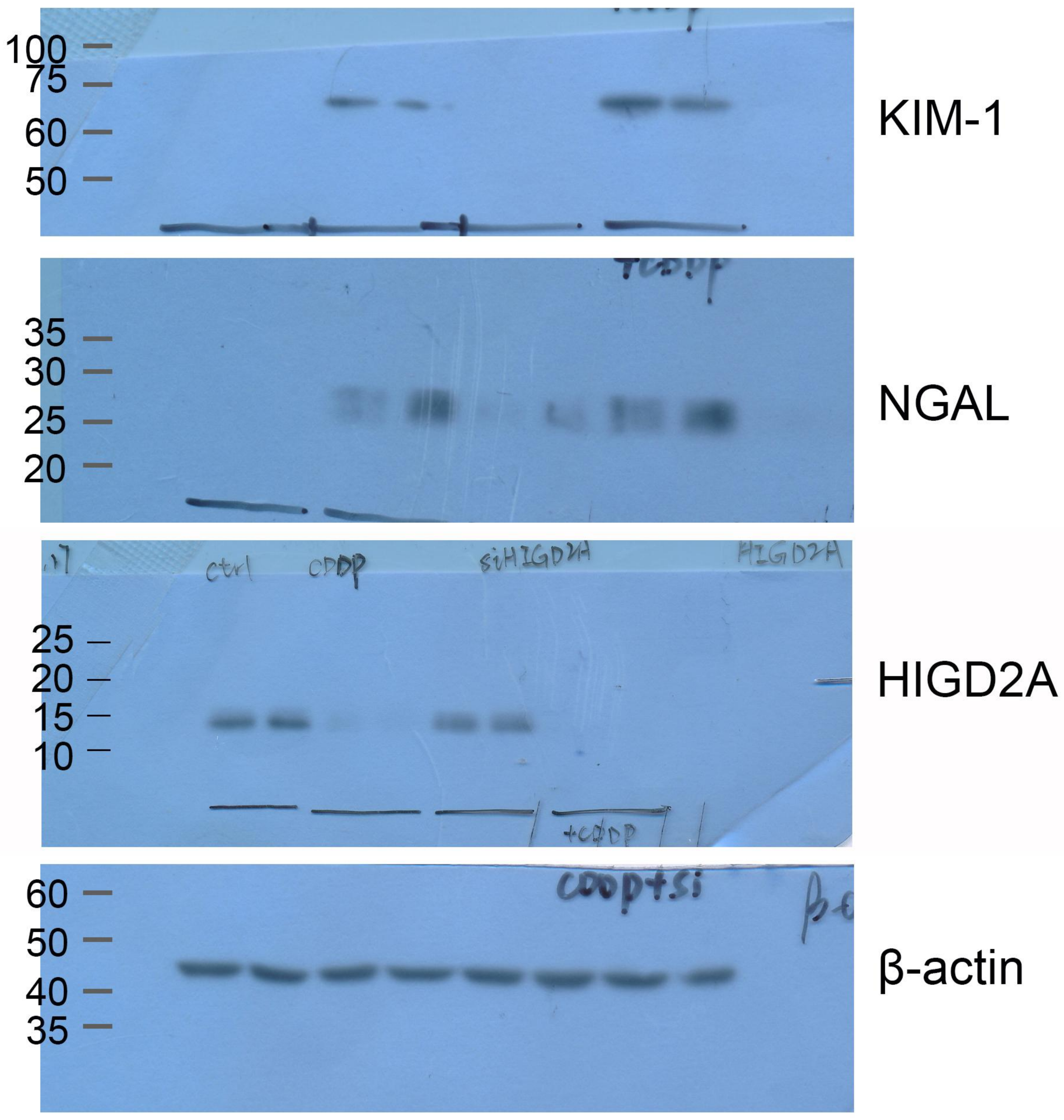

Figure 8.

c

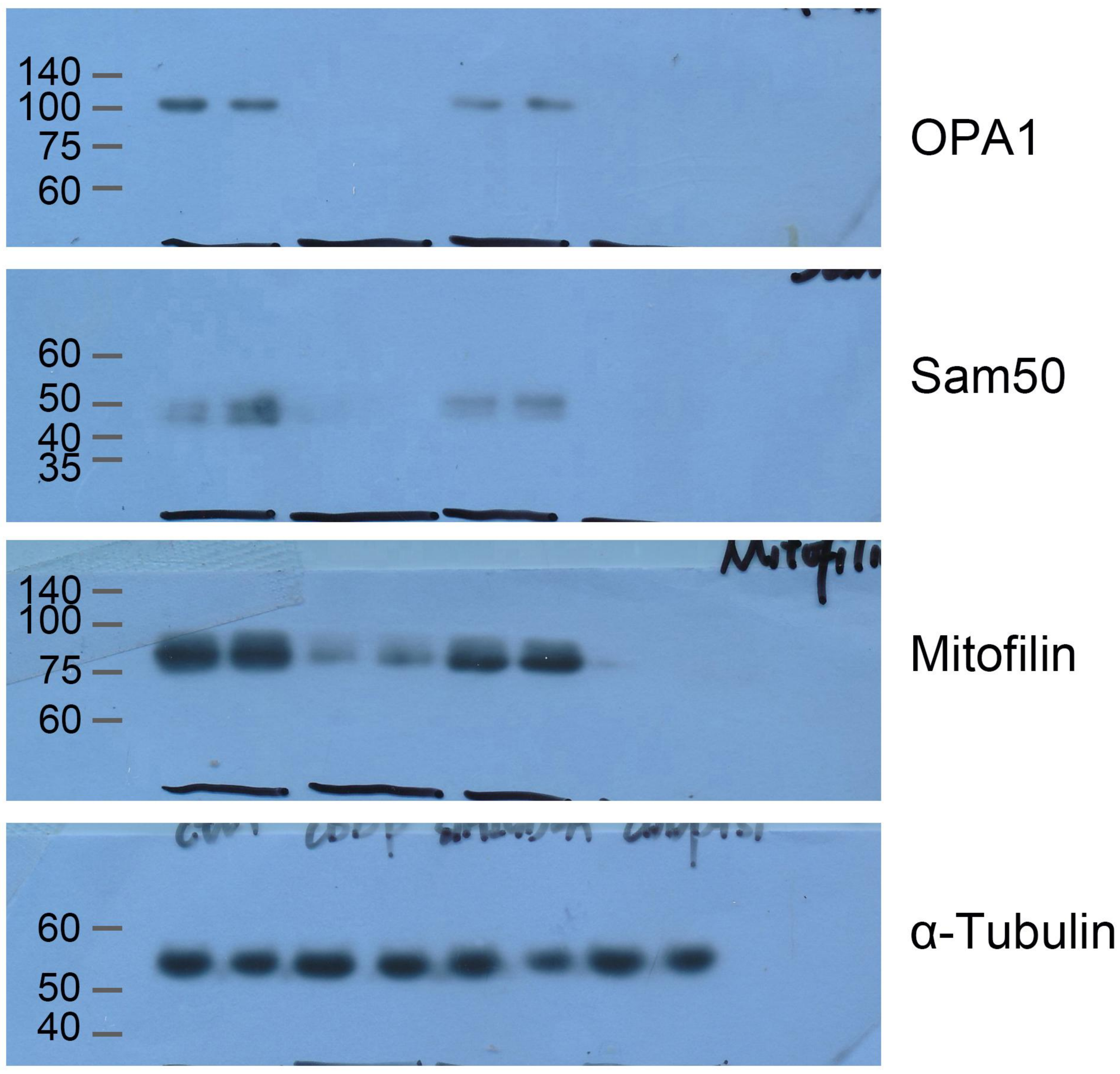

e

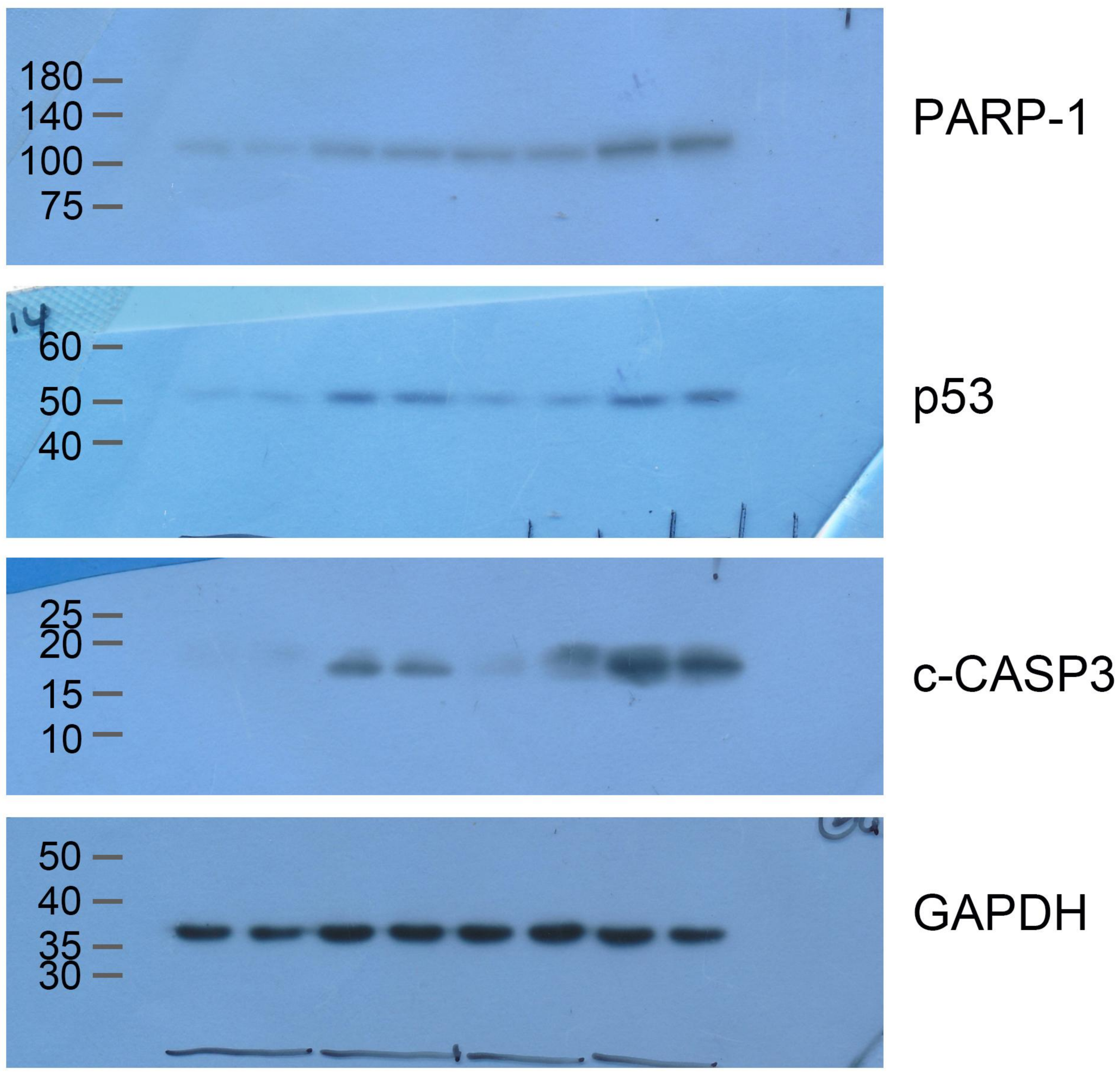

h

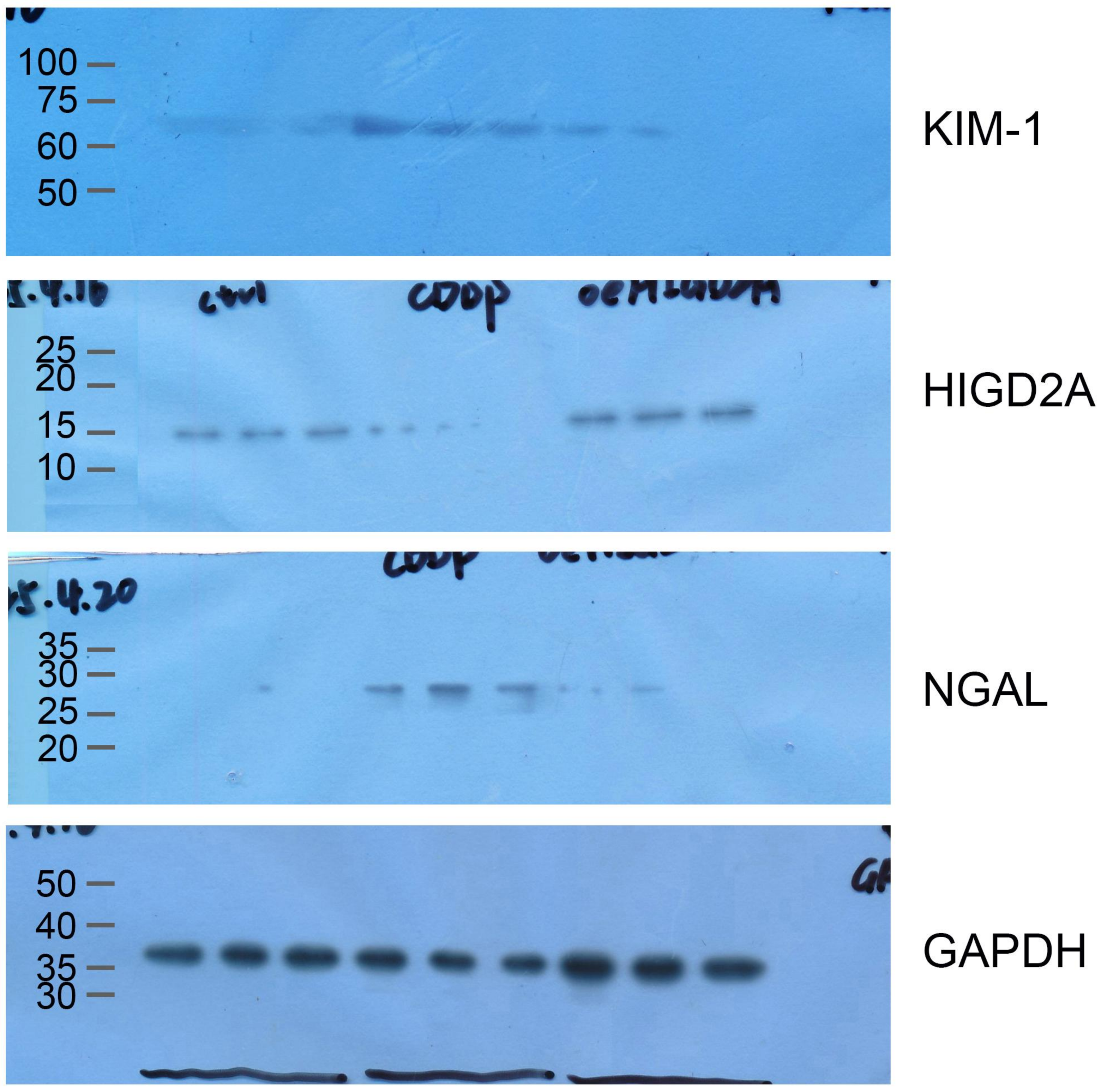

Figure 8.

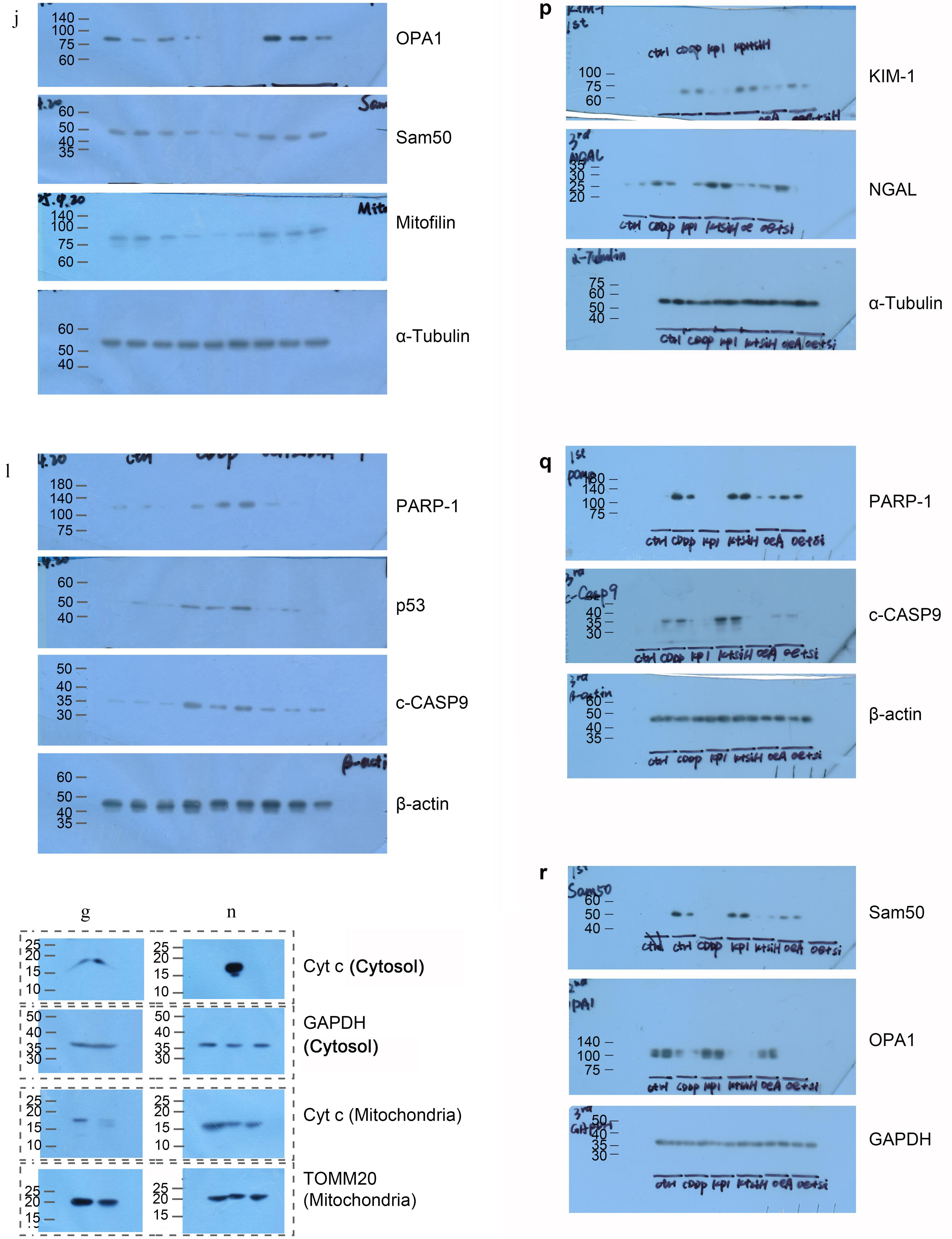
j
OPA1
Sam50
Mitofilin
α-Tubulin
l
PARP-1
p53
c-CASP9
β-actin
g
n
Cyt c (Cytosol)
GAPDH (Cytosol)
Cyt c (Mitochondria)
TOMM20 (Mitochondria)
p
KIM-1
NGAL
α-Tubulin
q
PARP-1
c-CASP9
β-actin
r
Sam50
OPA1
GAPDH

Figure 9.

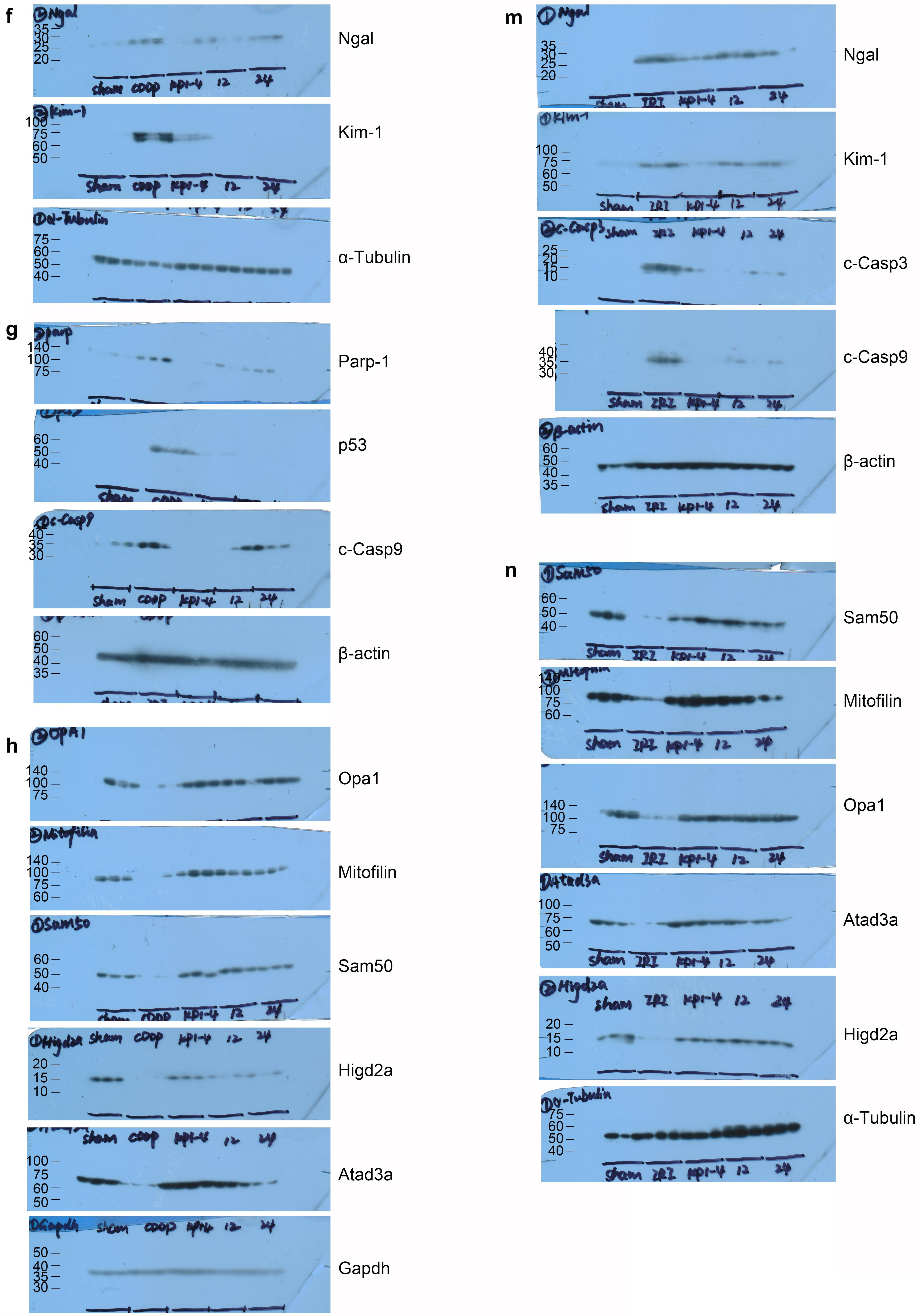
f
Ngal
Kim-1
α-Tubulin
g
Parp-1
p53
c-Casp9
β-actin
h
Opa1
Mitofilin
Sam50
Higd2a
Atad3a
Gapdh
m
Ngal
Kim-1
c-Casp3
c-Casp9
β-actin
n
Sam50
Mitofilin
Opa1
Atad3a
Higd2a
α-Tubulin

Figure S3.

a

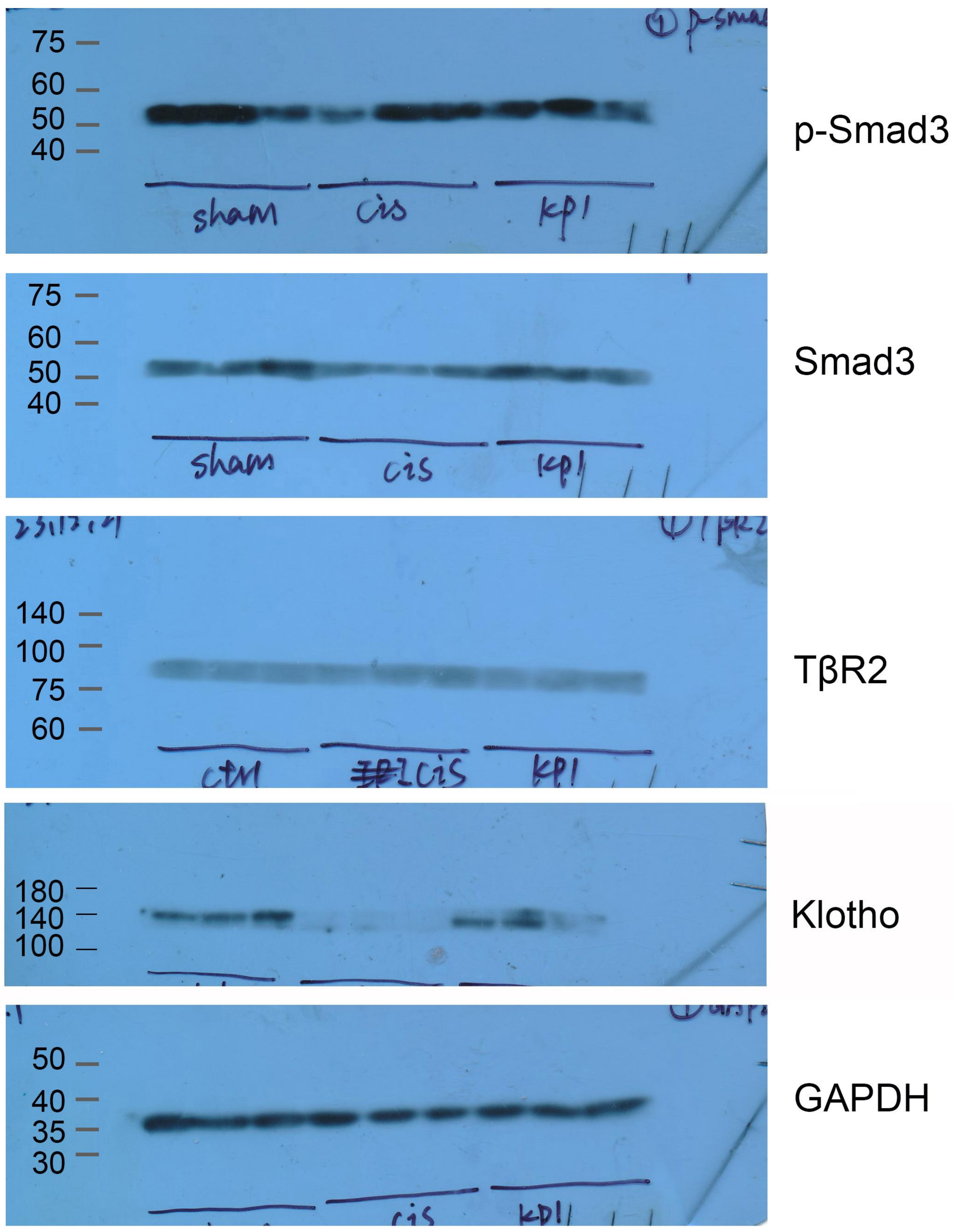

c

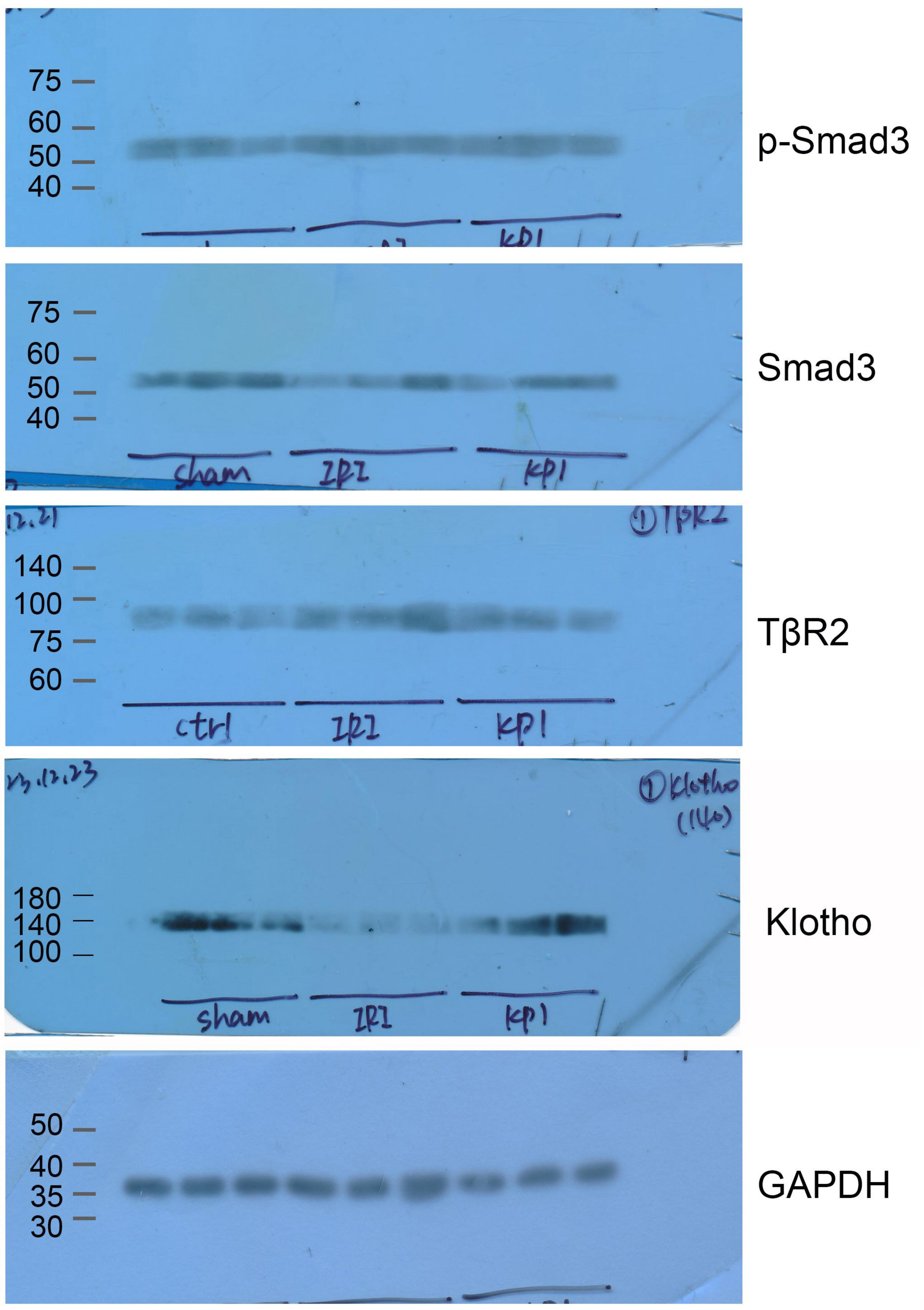

Figure S5.

b

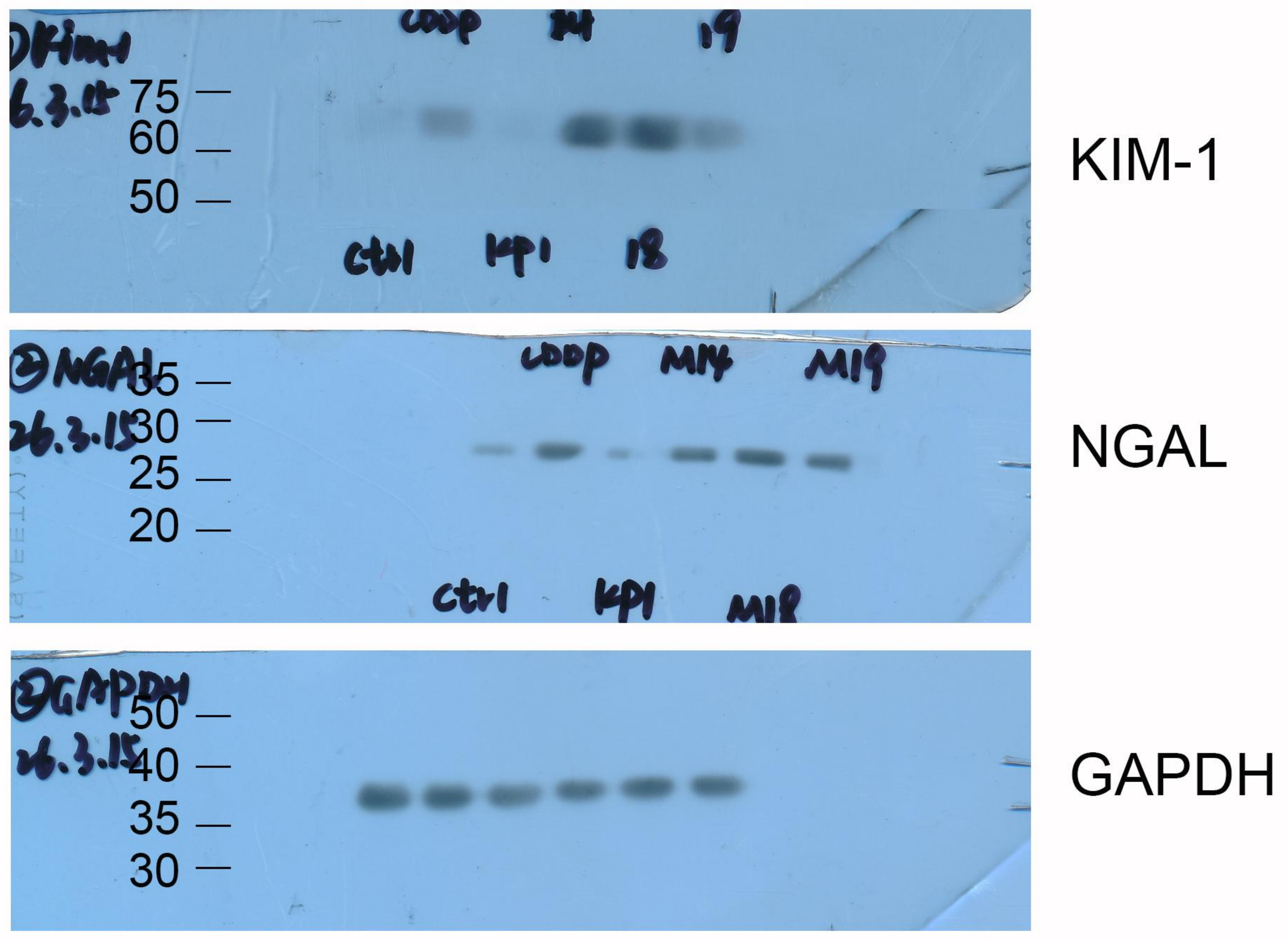

d

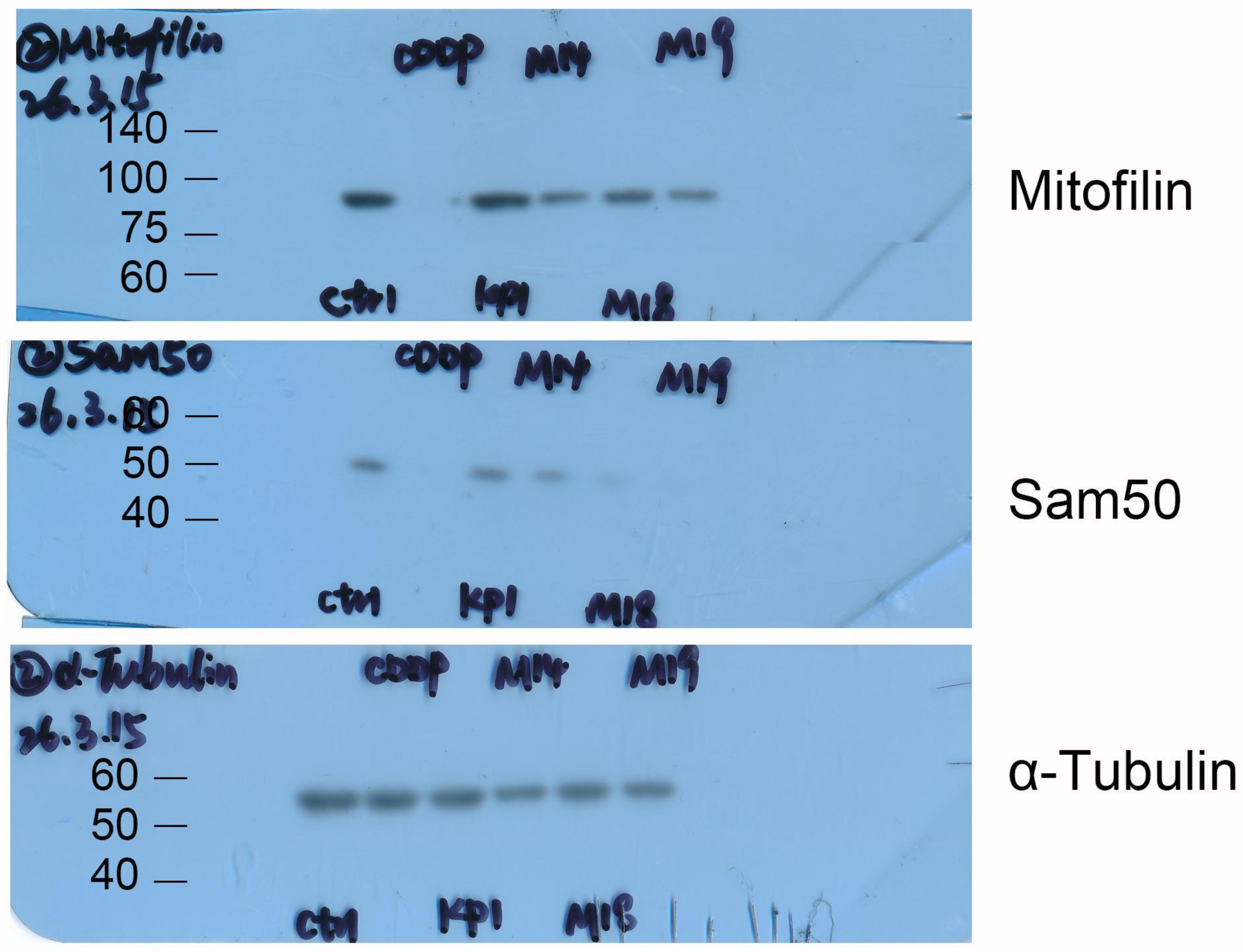

Figure S7.

b

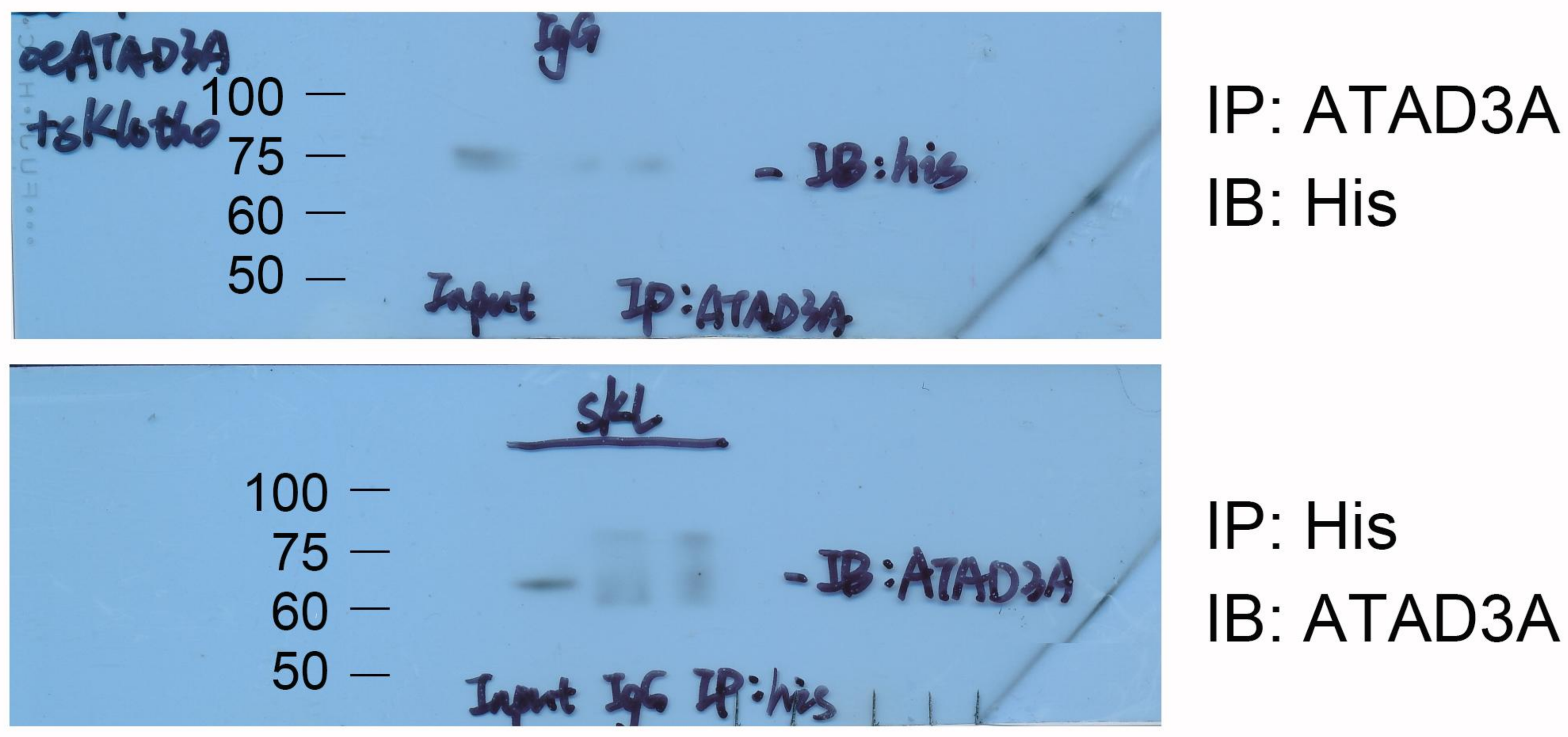

Figure S6.

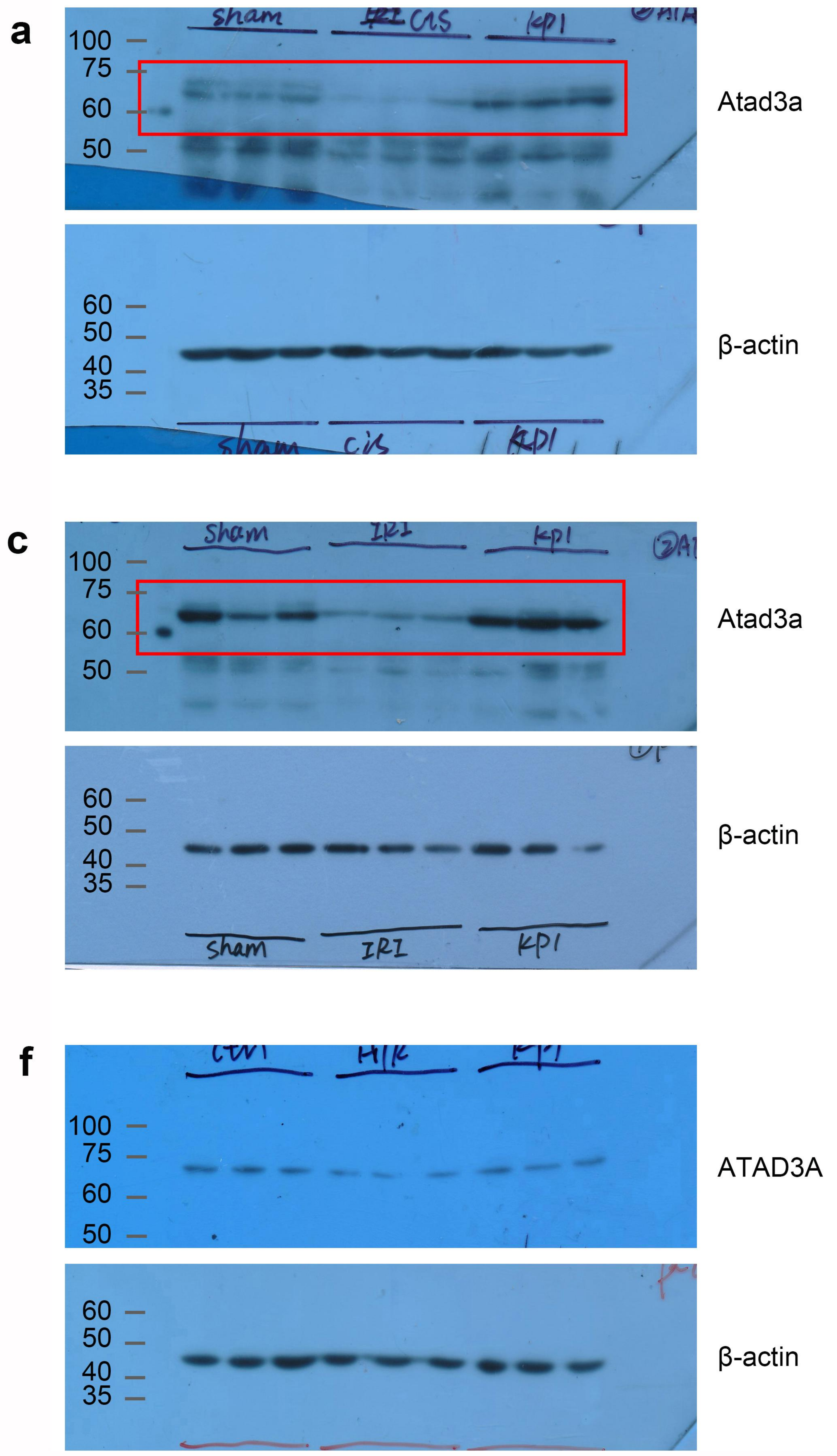

Figure S8.

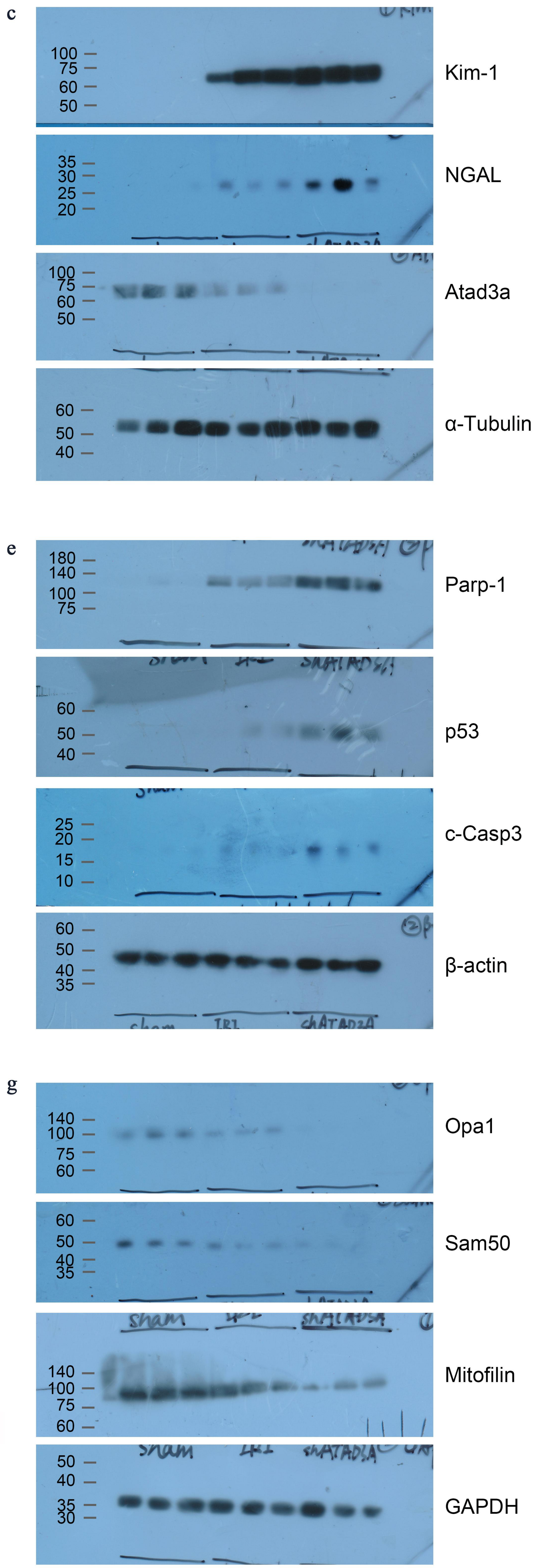

Figure S10.

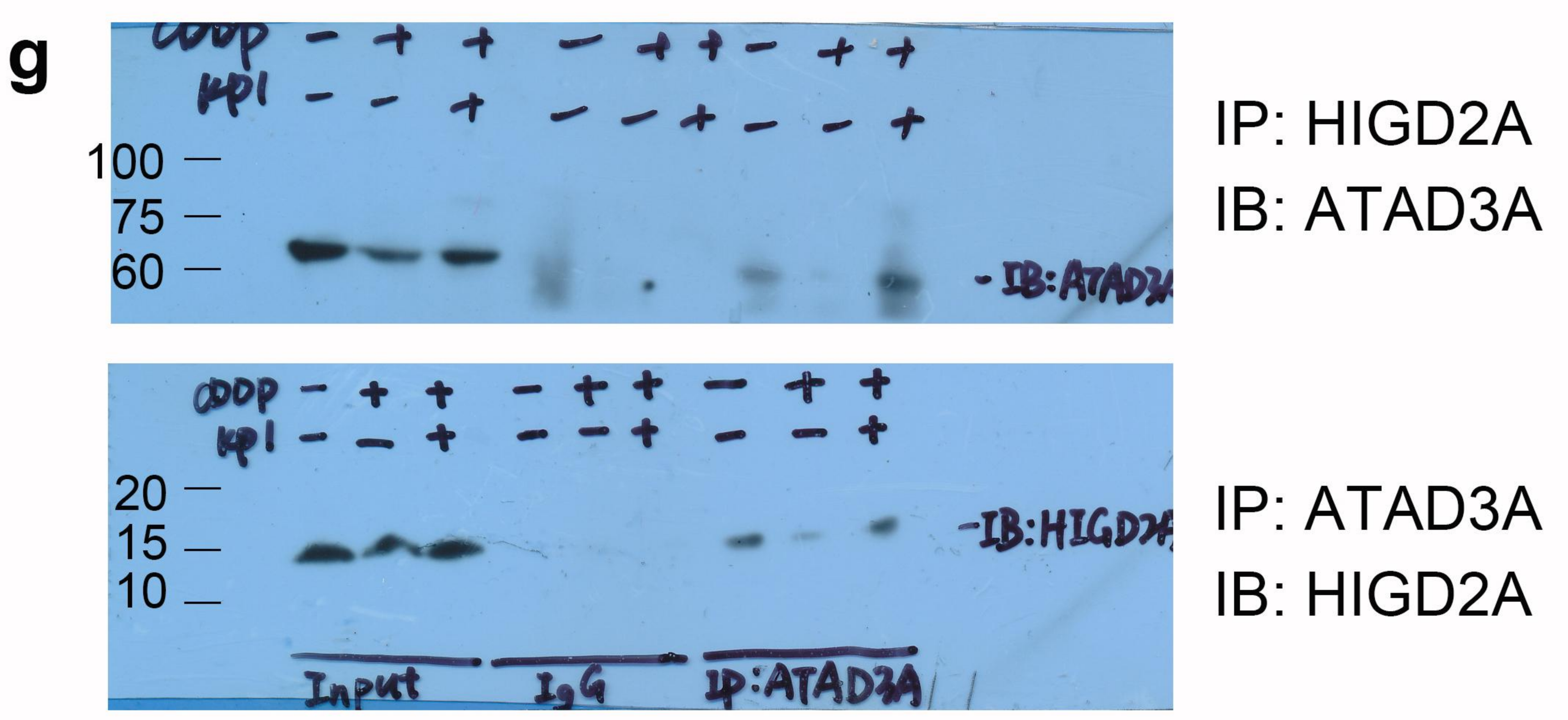

Figure S11

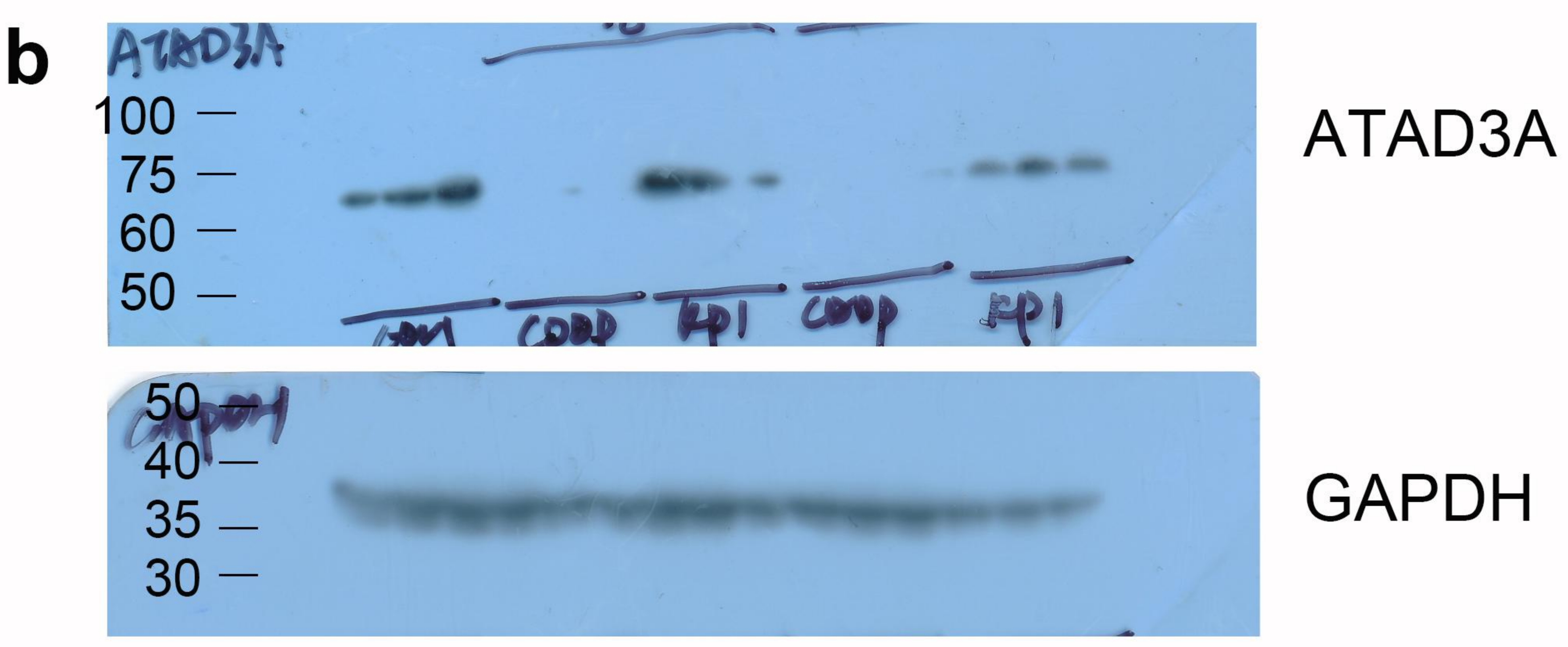

Figure S9

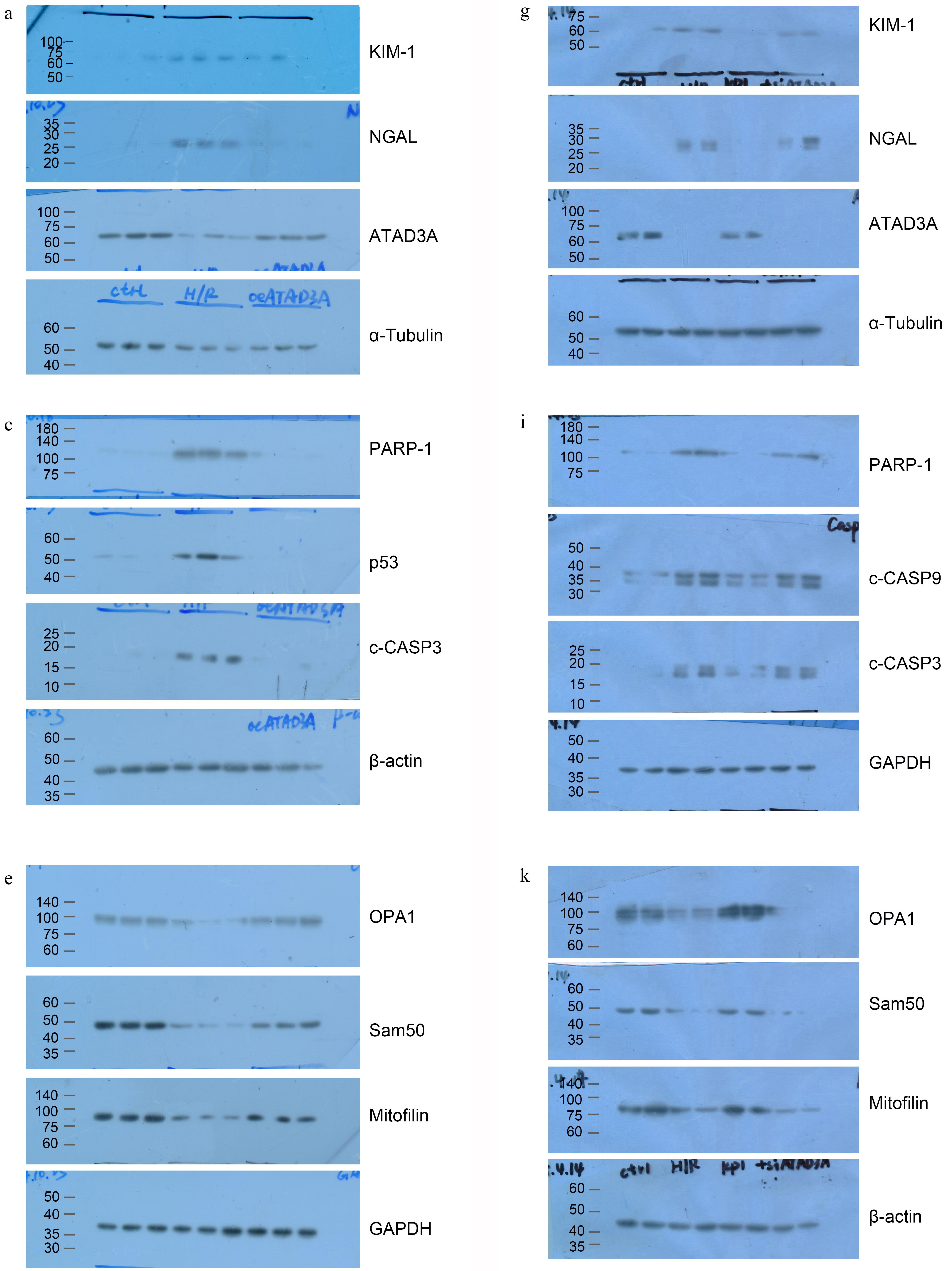
a
KIM-1
NGAL
ATAD3A
α-Tubulin
ctrl
H/R
oeATAD3A
c
PARP-1
p53
c-CASP3
β-actin
e
OPA1
Sam50
Mitofilin
GAPDH
g
KIM-1
NGAL
ATAD3A
α-Tubulin
i
PARP-1
c-CASP9
c-CASP3
GAPDH
k
OPA1
Sam50
Mitofilin
β-actin
