# Supplementary material for: Klotho‐Derived Peptide 1 Protects against Acute Kidney Injury by Directly Targeting Mitochondrial ATAD3A
Source: Adv Sci (Weinh). 2026 Aug 25:e77214. Online ahead of print. doi: 10.1002/advs.77214 (PMC13505471; doi:10.1002/advs.77214)
Supplement: Supplementary file 2 [file ADVS-9999-e77214-s004.pdf]

Klotho-derived Peptide 1 Protects Against Acute Kidney Injury by Directly Targeting Mitochondrial ATAD3A

Xiaoyao Zhang[1,2], Shihui Lin[1,2], Tianyu Wu[1,2], Zhixin Zhang[1,2], Hong Zhou[1,2], Xue Hong[1,2], Youhua Liu[1,2*]

Supplementary Figure

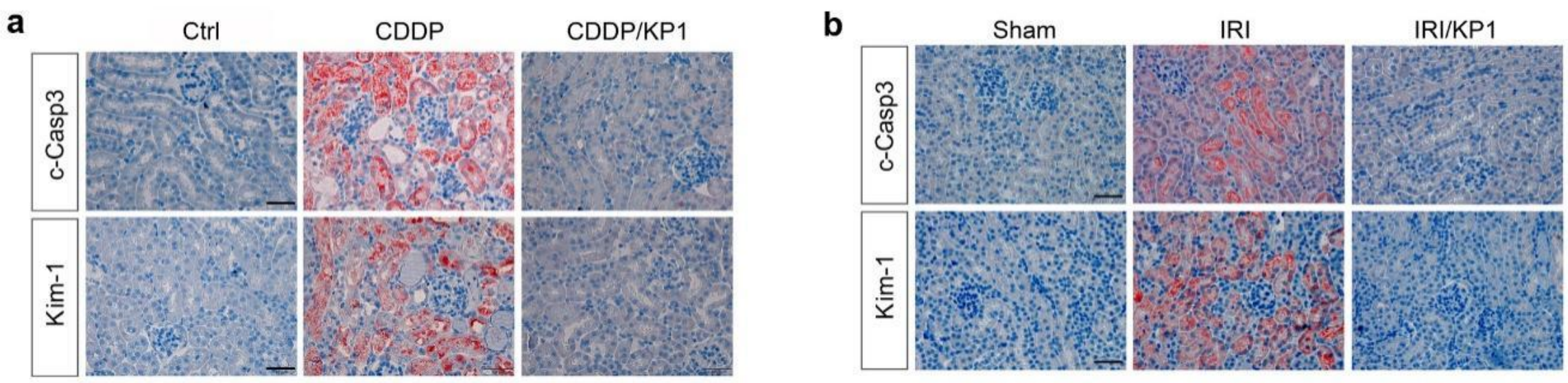

Fig. S1. KP1 protects against both nephrotoxic and ischemic kidney injury and apoptosis in mice. **(a)** Representative immunohistochemical micrographs of c-Casp3 and Kim-1 proteins in CDDP mice after KP1 treatment. Scale bar, 50 µm. **(b)** Representative immunohistochemical micrographs of c-Casp3 and Kim-1 proteins in IRI mice after KP1 treatment. Scale bar, 50 µm.

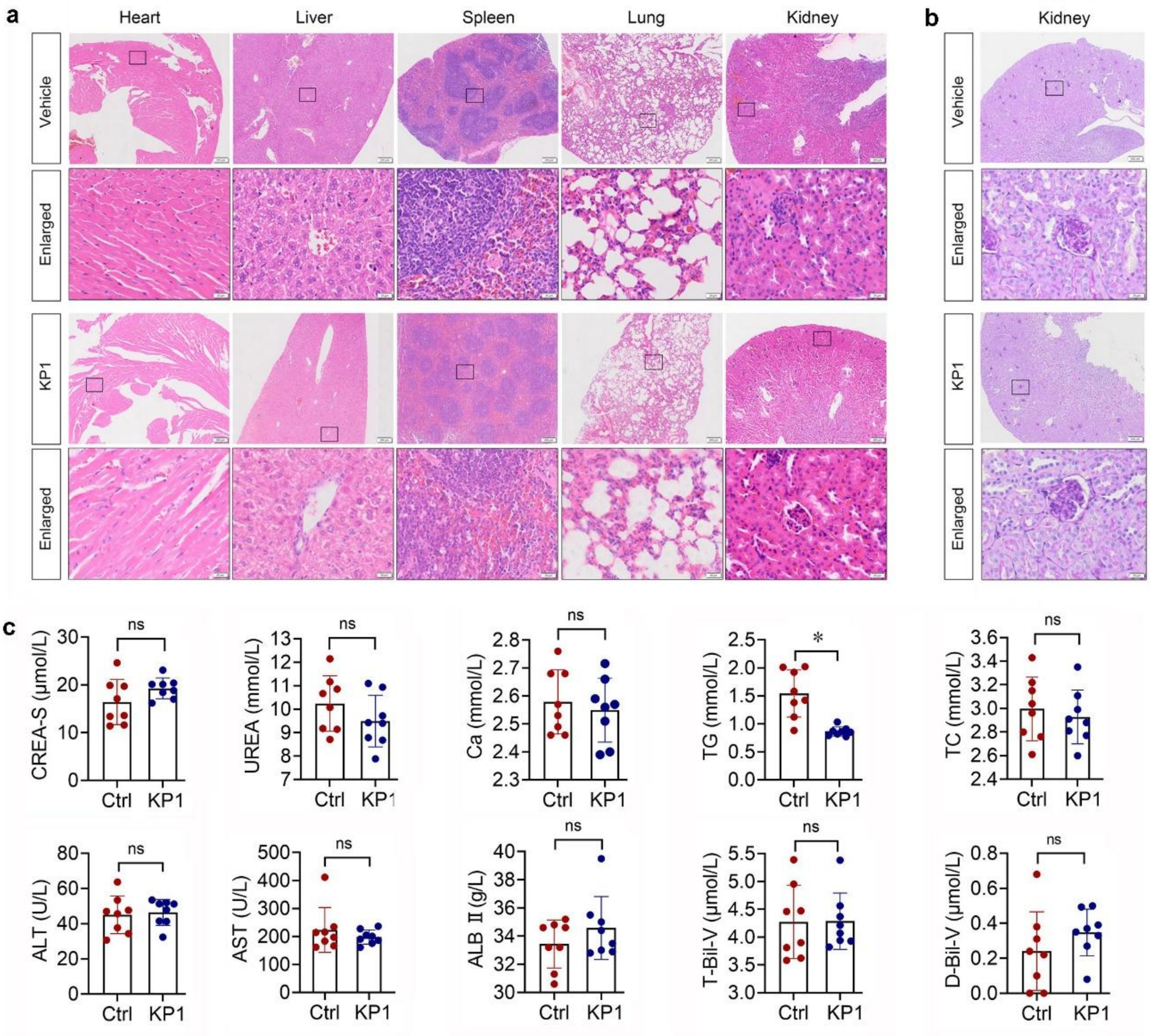

Fig. S2. KP1 displays no adverse side-effects in mice. (a) Representative HE staining of heart, liver, spleen, lung and kidney from mice treated with vehicle or KP1 (10 mg/kg/day) for 28 consecutive days, with enlarged views of the boxed areas. **(b)** Representative PAS staining of kidney. **(c)** Serum biochemical parameters including serum creatinine (CREA-S), blood urea nitrogen (UREA), calcium concentration (Ca), Triglyceride (TG), total cholesterol (TC), alanine aminotransferase (ALT), aspartate aminotransferase (AST), albumin 2 (ALB II), total bilirubin (T-Bil-V), direct bilirubin (D-Bil-V). Data are shown as mean ± SD with individual values plotted. $^{*}P < 0.05$. ns, no statistical difference.

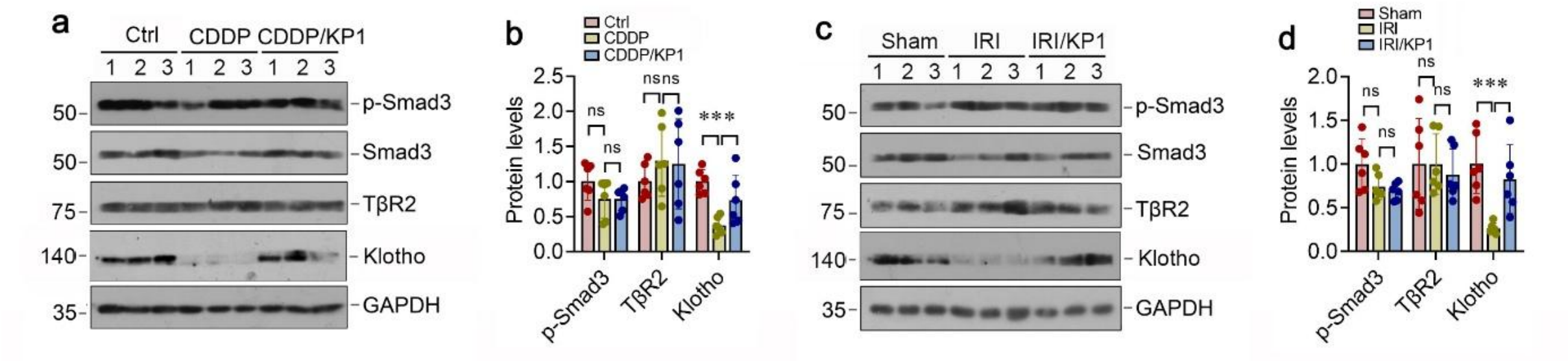

Fig. S3. KP1-mediated renoprotection after AKI is independent on TGF-β/Smad3 signaling but associated with restoration of Klotho protein. (a, b) Western blotting analysis for p-Smad3, TβR2 and Klotho protein levels in CDDP mice after KP1 treatment. **(c, d)** Western blotting analysis for p-Smad3, TβR2 and Klotho protein levels in IRI mice after KP1 treatment. $^{*}P < 0.05$; $^{**}P < 0.01$; ns, no statistical difference.

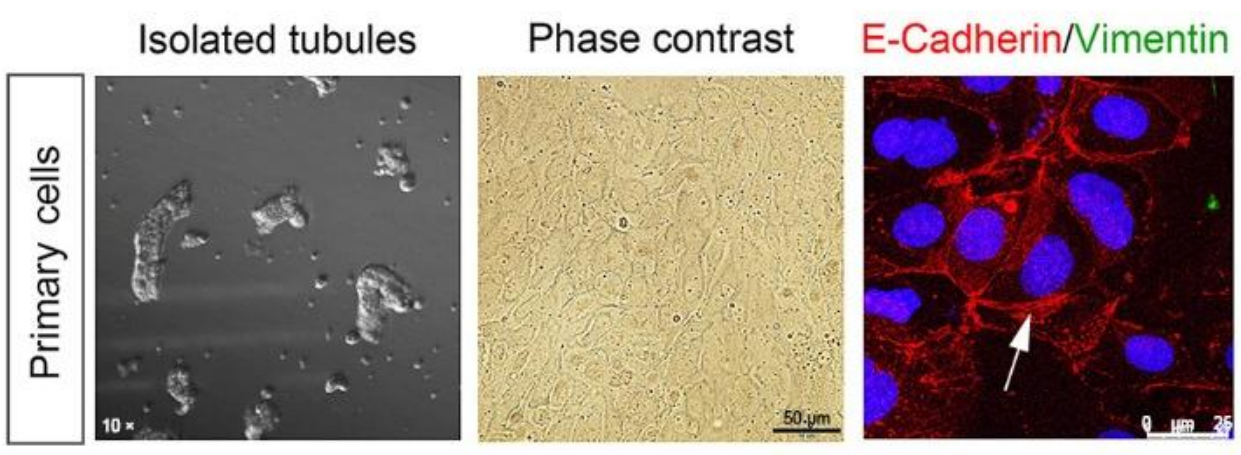

Fig. S4. Culture and characterization of mouse primary proximal tubular epithelial cells. Freshly isolated proximal tubules and primary tubular epithelial cells (phase contrast) are shown. Primary cells were characterized by immunostaining with specific antibodies against E-cadherin (red) and vimentin (green), respectively.

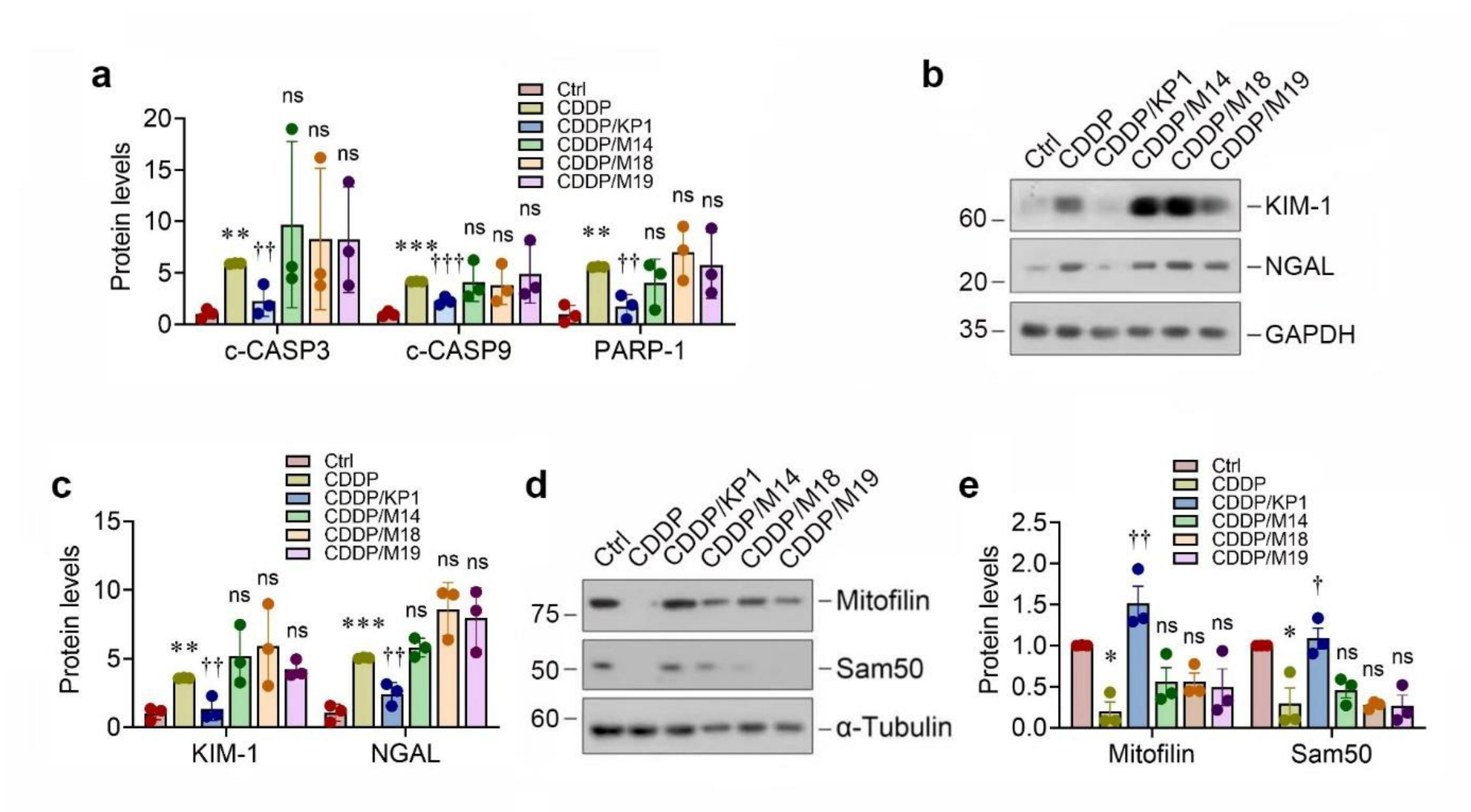

Fig. S5. KP1 mutants lose protection against CDDP-induced injury in HKC-8 cells. (a) Quantification of c-CASP3, c-CASP9 and PARP-1 protein levels in CDDP-stimulated HKC-8 cells in different groups as indicated. **(b, c)** Western blot analysis and quantitative data for KIM-1 and NGAL protein levels in HKC-8 cells after various treatments as indicated. **(d, e)** Western blot analysis and quantitative statistics for Mitofilin and Sam50 protein levels in HKC-8 cells after various treatment as indicated. $^{*}P < 0.05$, $^{**}P < 0.01$, $^{***}P < 0.001$ *vs* Ctrl; $^{\dagger}P < 0.05$, $^{\dagger\dagger}P < 0.01$, $^{\dagger\dagger\dagger}P < 0.001$ *vs* CDDP. ns, not significant.

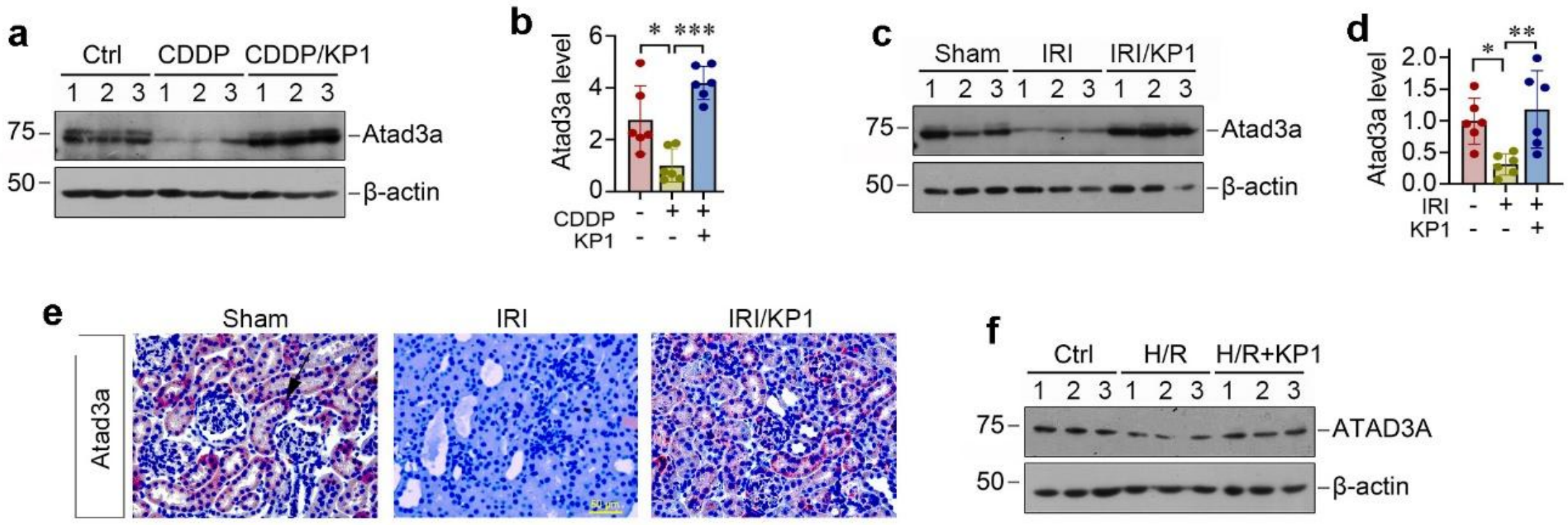

Fig. S6. KP1 restores Atad3a expression in AKI models *in vivo* and *in vitro*. (a, b) Western blotting analysis for Atad3a protein level in CDDP mice with KP1 treatment. **(c, d)** Western blot analysis for Atad3a protein level in IRI mice with KP1 treatment. **(e)** Representative immunostaining for Atad3a in IRI mice with KP1 treatment. Scale bar, 50 μm. **(f)** Representative Western blot showed that KP1 restored ATAD3A protein level in HKC-8 cells after various treatments as indicated. ns, not significant; $^{*}P < 0.05$; $^{**}P < 0.01$; $^{***}P < 0.001$.

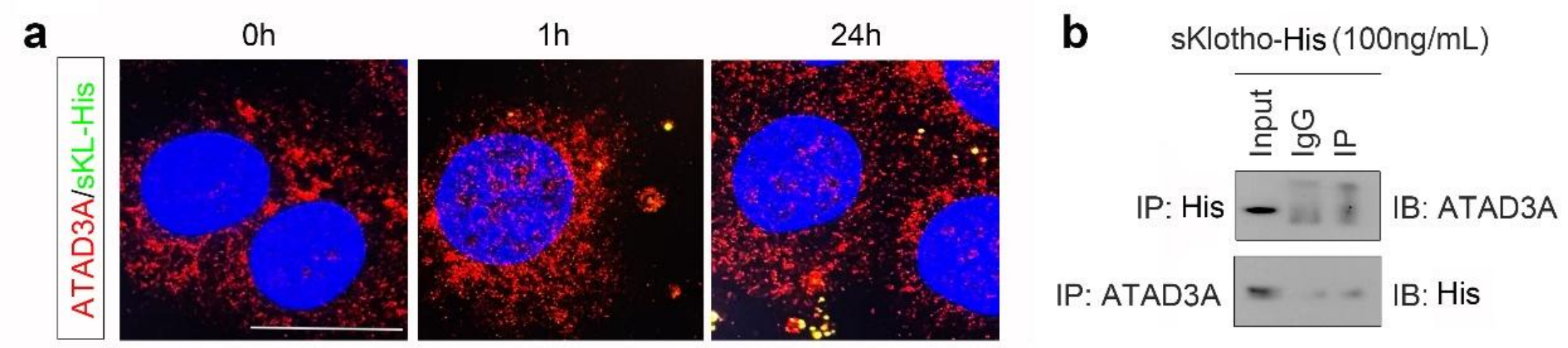

Fig. S7. His-tagged sKlotho neither enter to kidney cells nor interacts with ATAD3A. (a) Representative immunofluorescence micrographs of ATAD3A (red) and sKlotho-His (green) in HKC-8 cells. HKC-8 cells were treated with sKlotho-His for 0, 1, and 24 h. Scale bar, 50 μm. **(b)** Co-IP assays showed no detectable binding between sKlotho and ATAD3A. Cell lysates from HKC-8 cells were incubated with 100 ng/mL sKlotho-His, then immunoprecipitated (IP) with anti-His antibody at 4 ℃ overnight, followed by immunoblotting (IB) for ATAD3A. In the reciprocal experiment, mixtures were immunoprecipitated with anti-ATAD3A antibody, followed by IB for His.

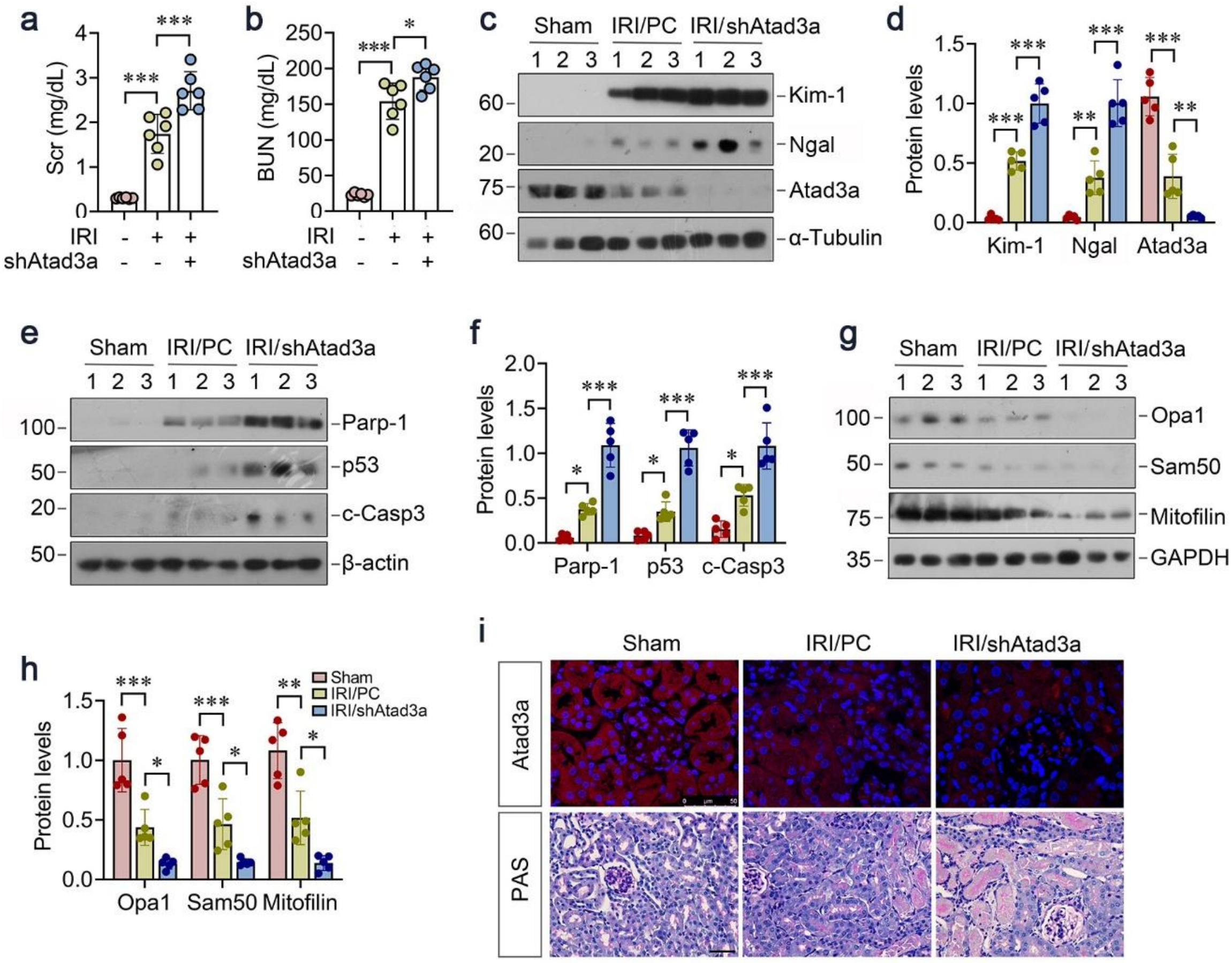

Fig. S8. Depletion of Atad3a aggravated IRI injury *in vivo.* (a, b) Graphic presentation showed Scr and BUN levels in IRI mice after knockdown of Atad3a. **(c)** Representative Western blot analyses of Kim-1, NGAL and Atad3a proteins in IRI mice after injection of sh-Atad3a plasmids. **(d)** Quantitative data of Kim-1, NGAL and Atad3a protein levels. **(e, f)** Western blotting analysis for Parp-1, p53 and c-Casp3 proteins in IRI mice after knockdown of Atad3a. **(g, h)** Western blotting analysis for Opa1, Sam50 and mitofilin proteins in IRI mice after knockdown of Atad3a. **(i)** Representative immunostaining for Atad3a and PAS staining in IRI mice after knockdown of Atad3a. Scar bar, 50 µm. $^{*}P < 0.05$; $^{**}P < 0.01$; $^{***}P < 0.001$.

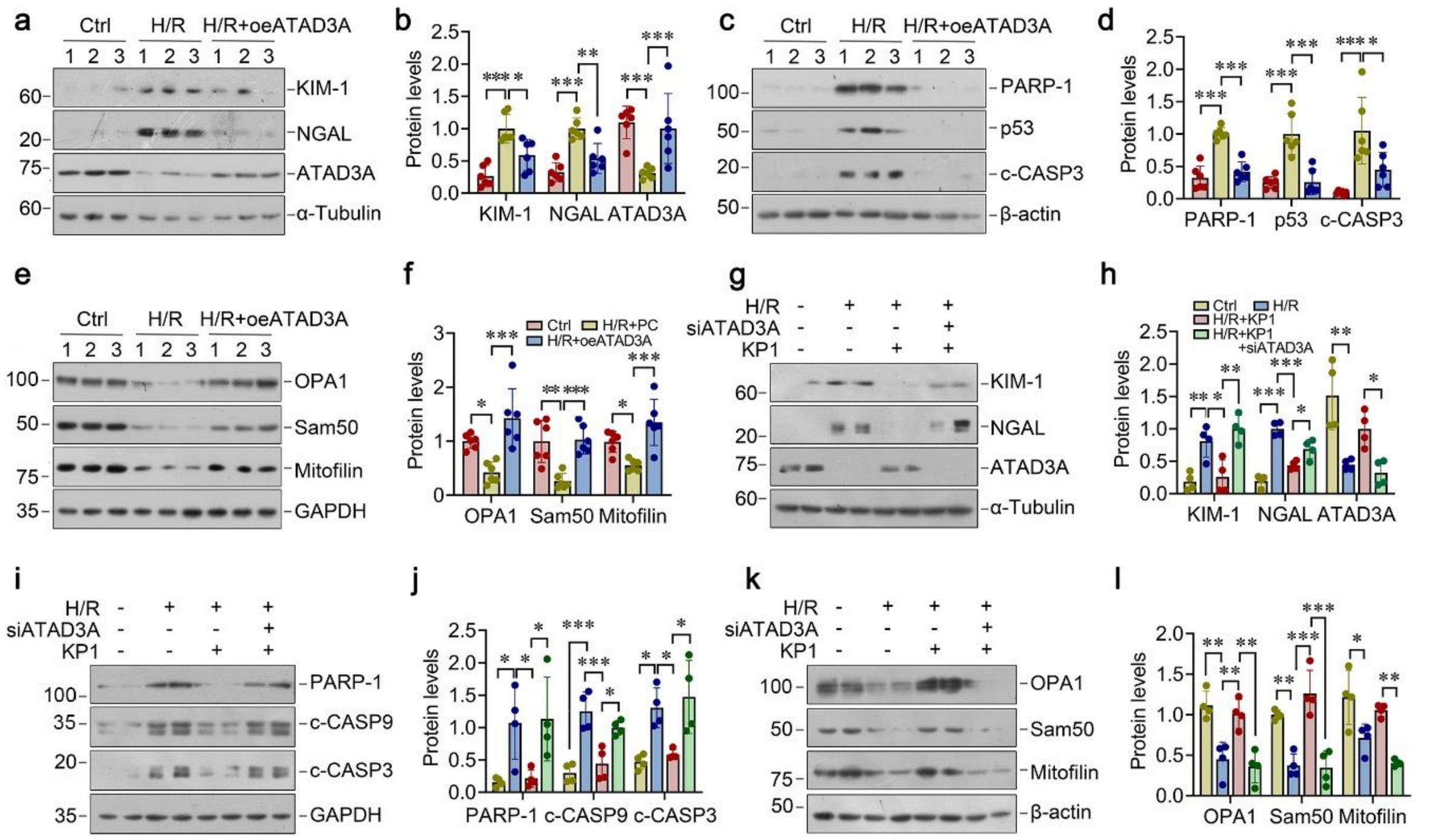

Fig. S9. ATAD3A alleviates H/R-induced cell injury and mitochondrial dysfunction *in vitro*. (a-d) Western blotting analysis for **(a, b)** KIM-1, NGAL and ATAD3A, **(c, d)** PARP-1, p53 and c-CASP3 proteins in different groups as indicated. **(e, f)** Western blotting analysis for OPA1, Sam50, mitofilin protein levels in different groups as indicated. **(g)** Representative Western blot of KIM-1, NGAL and ATAD3A proteins in different groups as indicated. **(h)** Quantitative data showed the levels of KIM-1, NGAL and ATAD3A proteins. **(i, j)** Western blotting analysis for PARP-1, c-CASP9 and c-CASP3 protein levels in different groups as indicated. **(k, l)** Western blotting analysis for OPA1, Sam50 and mitofilin protein levels in different groups as indicated. ns, not significant; $^{*}P < 0.05$; $^{**}P < 0.01$; $^{***}P < 0.001$.

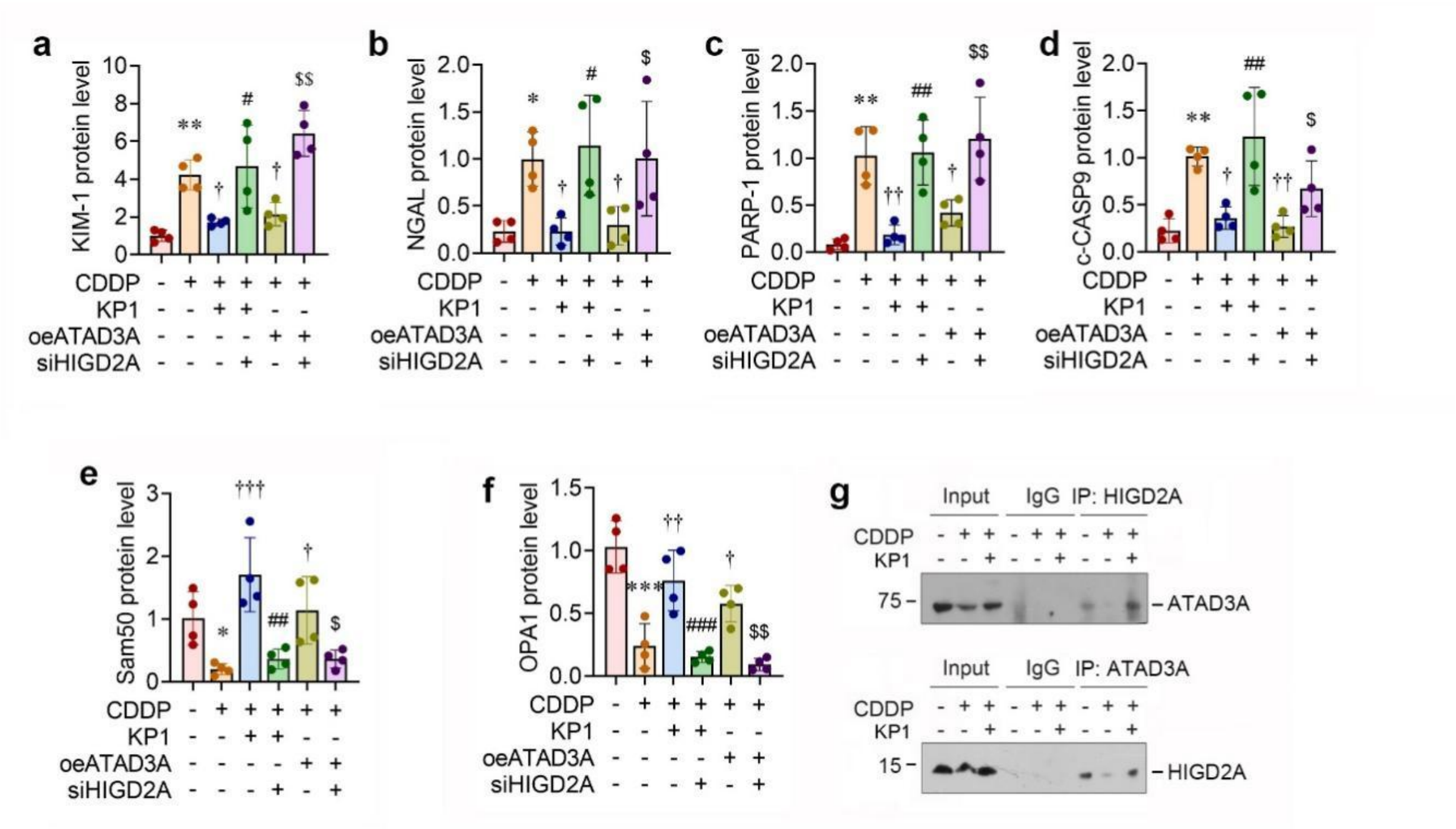

Fig. S10. KP1-mediated protection is dependent on mitochondrial ATAD3A and HIGD2A proteins. (a-f) Western blot quantitative data of KIM-1, NGAL, PARP-1, c-CASP9, Sam50 and OPA1 proteins in HKC-8 cells after various treatments as indicated. $^{*}P < 0.05$, $^{**}P < 0.01$, $^{***}P < 0.001$ *vs* control; $^{\dagger}P < 0.05$, $^{\dagger\dagger}P < 0.01$, $^{\dagger\dagger\dagger}P < 0.001$ *vs* CDDP; $^{\#}P < 0.05$, $^{\#\#}P < 0.01$, $^{\#\#\#}P < 0.001$ *vs* CDDP+KP1; $^{\$}P < 0.05$, $^{\$\$}P < 0.01$ *vs* CDDP+oeATAD3A. **(g)** KP1 restored ATAD3A/HIGD2A interaction after CDDP treatment. Cell lysates from different groups as indicated were immunoprecipitated (IP) with anti-ATAD3A antibody at 4 °C overnight, followed by immunoblotting (IB) for HIGD2A. In the reciprocal experiment, mixtures were immunoprecipitated with anti-HIGD2A antibody, followed by IB for ATAD3A.

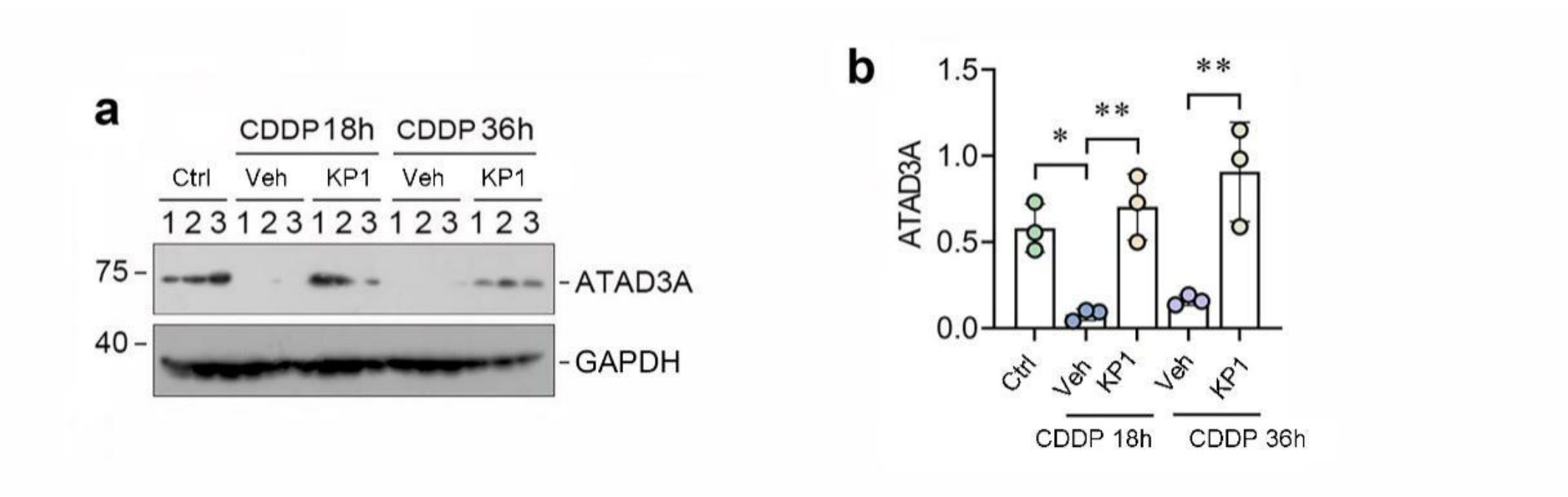

Fig. S11. KP1 restored ATAD3A protein expression at different time points after CDDP treatment. (a) Western blot detection of ATAD3A protein levels at 18 h and 36 h in different groups as indicated. **(b)** Quantification of ATAD3A protein expression. $^{*}P < 0.05$, $^{**}P < 0.01$.

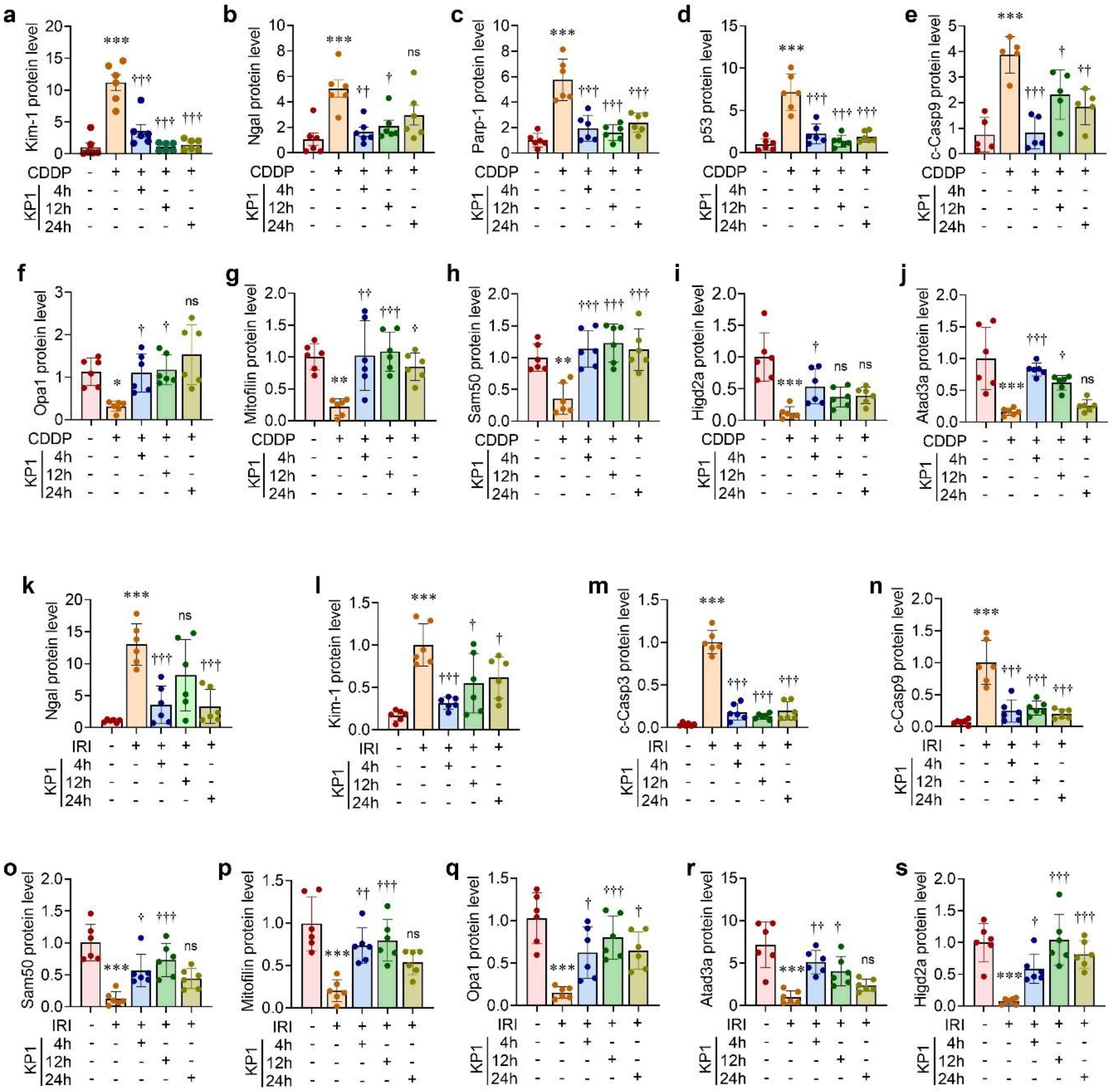

Fig S12. Delayed administration of KP1 still confers renal protection in an established AKI in mice. (a-j) Western blot quantitative data of renal Kim-1 **(a)**, Ngal **(b)**, Parp-1 **(c)**, p53 **(d)**, c-Casp9 **(e)**, Opa1 **(f)**, Mitofilin **(g)**, Sam50 **(h)**, Higd2a **(i)** and Atad3a **(j)** proteins in CDDP mice. KP1 was injected at different time points (4, 12, 24 h) after CDDP injection. **(k-s)** Western blot quantitative data of renal Ngal **(k)**, Kim-1 **(l)**, c-Casp3 **(m)**, c-Casp9 **(n)**, Sam50 **(o)**, Mitofilin **(p)**, Opa1**(q)**, Atad3a **(r)** and Higd2a **(s)** proteins in IRI mice. KP1 was injected at different time points (4, 12, 24 h) after IRI. ns, not significant; $^{*}P < 0.05$, $^{**}P < 0.01$, $^{***}P < 0.001$ *vs* Ctrl; $^{\dagger}P < 0.05$, $^{\dagger\dagger}P < 0.01$, $^{\dagger\dagger\dagger}P < 0.001$ *vs* CDDP or IRI.

VIDEO LEGENDS

Video S1. The label free live cell microscopy video of CDDP-stimulated HKC-8 cells. Scale bar, 20 μm. Arrow, mitochondria. Timeline, 0-36 h.

Video S2. The label free live cell microscopy video of CDDP-stimulated HKC-8 cells with KP1 treatment. Scale bar, 20 μm. Arrow, mitochondria. Timeline, 0-36 h.

Table S1. The sources of antibodies used in this study

Antibodies	Company	Cat. No	Location
**Primary antibodies**			
TIM 1	Abcam	ab316854	Waltham, MA, USA
Lipocalin-2 / NGAL	Abcam	ab63929	Waltham, MA, USA
NGAL	Santa Cruz Biotechnology	sc-518095	Dallas, TX, USA
PARP	Cell Signaling Technology	9532S	Danvers, MA, USA
p53	Cell Signaling Technology	2524S	Danvers, MA, USA
Cleaved Caspase-3 (Asp175)	Cell Signaling Technology	9664S	Danvers, MA, USA
OPA1	Abclonal	A9833	Wuhan, China
SAMM50/SAM50	Abcam	ab246987	Waltham, MA, USA
Mitofilin	Proteintech	10179-1-AP	Rosemont, IL, USA
FITC	NeoBiotechnologies	MSM1X-1277	Union City, CA, USA
ATAD3A	Novus Biologicals	NBP2-14881	Centennial, CO, USA
HIGD2A	Santa Cruz Biotechnology	sc-390505	Dallas, TX, USA
Cytochrome C	Abcam	ab13575	Waltham, MA, USA
Caspase-9	Cell Signaling Technology	9508S	Danvers, MA, USA
E-cadherin	Santa Cruz Biotechnology	sc-8426	Dallas, TX, USA
E-Cadherin	Cell Signaling Technology	14472S	Danvers, MA, USA
Vimentin	Abcam	ab92547	Waltham, MA, USA
TOM20/TOMM20	BOSTER	BM4366	Wuhan, China
GAPDH	Beijing Ray Antibody Biotech	RM2002	Beijing, China
α-tubulin	Beijing Ray Antibody Biotech	RM2007	Beijing, China
β-actin	Beijing Ray Antibody Biotech	RM2001	Beijing, China
DYKDDDDK (FLAG)	Sigma-Aldrich	MAB3118	St. Louis, MO, USA
Normal Rabbit IgG	R&D Systems	AB-105-C	Minneapolis, MN, USA
Normal Rabbit IgG	MBL	PM035	Beijing, China
Normal Mouse IgG	Sigma-Aldrich	NI03	St. Louis, MO, USA
Phospho-Smad3	ABclonal	AP0727	Wuhan, China
SMAD2/3	Cell Signaling Technology	8685S	Danvers, MA, USA
TGF beta Receptor 2/TGFBR2	Santa Cruz Biotechnology	sc-17791	Dallas, TX, USA
Klotho	R&D Systems	AF1819	Minneapolis, MN, USA
Phospho-NF-κB p65	Cell Signaling Technology	3033S	Danvers, MA, USA
NF-κB p65	Cell Signaling Technology	4764S	Danvers, MA, USA
**Secondary antibodies**			
Peroxidase Goat anti-Mouse	Jackson ImmunoResearch	115-035-003	West Grove, PA, USA
Peroxidase Rabbit anti-Goat	Jackson ImmunoResearch	305-035-003	West Grove, PA, USA

Peroxidase Goat anti-Rabbit	Jackson ImmunoResearch	111-035-003	West Grove, PA, USA
Biotin-SP Donkey anti-Rabbit	Jackson ImmunoResearch	711-065-152	West Grove, PA, USA
Biotin-SP Donkey anti-Mouse	Jackson ImmunoResearch	715-065-150	West Grove, PA, USA
Cy™3 Donkey anti-Mouse	Jackson ImmunoResearch	715-165-151	West Grove, PA, USA
Cy™3 Donkey anti-Rabbit	Jackson ImmunoResearch	711-165-152	West Grove, PA, USA
Cy™2 Mouse anti-Rabbit	Jackson ImmunoResearch	211-222-171	West Grove, PA, USA
Cy™5 Donkey anti-Rabbit	Jackson ImmunoResearch	711-175-152	West Grove, PA, USA

Table S2. The nucleotide sequences of the primers used for qPCR

Gene	Primer Sequences 5' to 3'	
	Forward	Reverse
ATAD3A (homo)	CGCCATAGCAACAAGGAACACC	ATGGCGTAGTCCATGCCTGAGT
Atad3a (mmu)	GGAGCTGAGGCATAAAAACGA	TCGAATCTGTTCCCGGATGAT
ACTB (homo)	GCAAAGACCTGTACGCCAACA	TGCATCCTGTCGGCAATG
Actb (mmu)	GGCTGTATTCCCCTCCATCG	CCAGTTGGTAACAATGCCATGT

Table S3. The nucleotide sequences of Oligo DNA/RNA used in this study

Oligo DNA/RNA		Sequences 5'to 3'
pGPU6/GFP/Neo-Atad3a (mouse)	S	CACCGACAGGACAGCACAGTAGTAATTCAAGAGATTACTACTGTGCTGTCCTGTCTTTTTTG
	A	GATCCAAAAAAGACAGGACAGCACAGTAGTAATCTCTTGAATTACTACTGTGCTGTCCTGTC
pGPU6/GFP/Neo-shNC	S	CACCGTTCTCCGAACGTGTCACGTCAAGAGATTACGTGACACGTTCGGAGAATTTTTTG
	A	GATCCAAAAAATTCTCCGAACGTGTCACGTAATCTCTTGACGTGACACGTTCGGAGAAC

Product Name	Company	Catalogue number
HIGD2A siRNA (h)	Santa Cruz Biotechnology	sc-91606
ATAD3A siRNA (h)	Santa Cruz Biotechnology	sc-88047
Control siRNA-A	Santa Cruz Biotechnology	sc-37007
